# “Everything in this world has been given to us from cows”, a qualitative study on farmers’ perceptions of keeping dairy cattle in Senegal and implications for disease control and healthcare delivery

**DOI:** 10.1371/journal.pone.0247644

**Published:** 2021-02-25

**Authors:** Laura Craighead, Jacqueline M. Cardwell, Bhagyalakshmi Chengat Prakashbabu, Elhadji Ba, Imadidden Musallam, Rianatou Bada Alambédji, Justin Ayih-Akakpo, Javier Guitian, Barbara Häsler

**Affiliations:** 1 Veterinary Epidemiology, Economics and Public Health Group, Department of Pathobiology and Population Sciences, Royal Veterinary College, Hertfordshire, United Kingdom; 2 Institut de Recherche Pour le Développement (IRD), Dakar, Senegal; 3 Ecole Inter-Etats des Sciences et Médecine Vétérinaires de Dakar, Dakar, Senegal; University of Illinois College of Veterinary Medicine, UNITED STATES

## Abstract

The dairy industry in Senegal is growing and evolving against a backdrop of rapid urbanisation and increasing consumer demand for dairy products. Consideration of appropriate cattle healthcare delivery and disease control in these evolving farming systems is of paramount importance given the risks posed by zoonotic pathogens and the economic consequences of disease for livestock keepers. Planning and implementation of disease control and healthcare delivery generally follows a top down approach. Often this does not take into account the views and perceptions of the farmers it impacts and who must behave in the expected way for successful outcomes to materialise. In this study, we asked 76 farmers to discuss their experience and opinions of farming milk producing cattle in 11 focus group discussions conducted in two peri-urban areas of Senegal. The objectives were to investigate farmers’ perceptions of the current conditions in farming, to understand how these might impact the future direction of this particular system and how this might affect the feasibility and appropriate methods of cattle healthcare delivery and disease control. The data collected were subjected to thematic analysis and four themes were identified; 1. Revered cattle, 2. The changing face of livestock keeping, 3. Powerlessness, 4. Optimism for the future. Farmers in our study had a deep affinity with their cattle, they respected the traditions surrounding cattle keeping at the same time as striving for advances within the system and their animal’s productivity. Within strong social groupings and hierarchical structures they recognised the inherent challenges they face but were hopeful and optimistic about growth and opportunity in the future of milk production. A holistic approach to embedding healthcare delivery and disease control within the broader context in which farmers operate may prove successful. This could involve consideration of funding channels for farmers, access to appropriate inputs and utilising the strong community spirit and social norms of farmers to initiate and facilitate change.

## Introduction

West Africa has one of the fastest population growths globally, the population having doubled over the last thirty years [1]. These demographic shifts occur in a landscape of rapid urbanisation in many countries, which is accompanied by evolving dietary preferences and demands as lifestyles change. It is commonplace that with such shifts an increase in demand for animal source foods including dairy produce is seen [2]. This in turn requires the dairy sector to evolve in order to meet demand and capitalise on the growing market. Growth of the dairy sector in Senegal seems to be prominent [3] with dairy cattle numbers increasing some 57% in the 10 year period from 2003–2013 according to FAOSTAT data [4].

Senegal is a coastal, francophone country in the West African region with a population of 15 million as of 2018 [1]. It is predominantly Muslim (94%) although many customs and behaviours throughout the country derive from traditional animism concepts [5]. Many ethnic groups cohabit amicably in all regions of Senegal with Wolof being the largest population (43% of total population) followed by Pulaar (24% of total population) and then Serer (15% of total population) [6]. While each ethnic group has defined histories and languages, Senegal has experienced ‘Wolofisation’ with people of different ethnicities speaking Wolof especially in urban areas [7]. Historically, Wolof people were settled farmers and artisans while Pulaar were nomadic herders. The Serer were known as landowners and practised mixed farming, often utilising Pulaar herders to manage their herds in transhumance [8]. In modern times, ethnic roles and employment are much less defined as people adapt their household income generation to opportunities and changing environments [8].

Demand for dairy products in Senegal is high and exceeds production, resulting in dependence on imports of milk powder [9]. Dependence on milk powder despite the increase in the number of dairy cattle suggests that cattle numbers are still insufficient or, more likely, cattle are only producing small amounts of milk per head. In Senegal it is estimated that local breed cattle produce an average of 300 litres/year [10]. The majority of milk available in large commercial retailers in most towns and cities is still produced and imported by large dairy companies such as Arla and Nestle. Locally-produced milk is available predominantly through informal channels. The government’s vision is to expand and modernise the in-country dairy sector to reduce the dependence on imports and to promote the dairy industry in line with the Senegalese National Plan (Plan Sénégal Emergent PSE), which aims to improve agriculture, livestock and food security by enhancing productivity and profitability. These plans will likely accelerate the intensification changes already seen in the dairy sector and could indeed worsen problems associated with cattle health and zoonotic disease unless managed appropriately. It has been suggested that important diseases such as brucellosis may be more prevalent in evolving and more intensified systems such as those that would be promoted for enhanced commercialisation of milk production [11].

In order to enhance local dairy production and to ensure public health through mitigation of food borne disease risks, cattle health and disease control must be prioritised in these emerging livestock systems. While it is vital to understand the epidemiological processes and disease risks which become prevalent in more intensive and commercialised systems, it is also paramount to understand the context of the system and the drivers and perceptions of the actors whose behaviour will dictate the success or failure of any programmes implemented. To understand the complexities involved in a setting where there are considerable influences from social, economic, environmental and biological drivers, a mixture of disciplines and methodologies are required. However, the majority of research within farming populations in Sub Saharan Africa on knowledge, attitudes and practices remain quantitative in nature [12–14]. In the past decade the value of social science approaches has been increasingly recognised in animal health and welfare research, as they allow investigating in depth social, economic, historical and cultural factors that shape the contexts within which disease occurs [15–22]. Using a social science lens, the boundaries of a study can be expanded to analyse wider dimensions such as economic, political and geographical factors that influence disease occurrence, transmission and measures for control [23].

With this in mind, the objectives of this qualitative study were to investigate Senegalese dairy cattle farmers’ perceptions of current conditions in farming, to understand how these might impact the future direction of this particular system and how this might affect the feasibility and appropriate methods of cattle healthcare delivery and disease control. This understanding would then allow evaluation of how these views and practices may align or differ from government’s intentions for the sector, with a focus on how cattle healthcare delivery and disease control may be integrated into evolving systems.

### Previous research on the perceptions and practices of farmers

An increasing body of research utilizes various social science methods and theories to investigate farmer’s perceptions, attitudes and behaviours, how these are constructed and how they change and develop over time [14, 18, 19, 23–29]. Many researchers utilise the lens of Bourdieu’s social capital [30] to explore the concept of being a good farmer and how this impacts individual and group behaviour [18, 20–22, 25–29]. Bourdieu suggests there are three primary forms of capital, these being: economic capital (material and financial property), social capital (networks of social connections and mutual obligations) and cultural capital (prestige, status in the community). Social relations are reinforced through the production and reproduction of these capitals, achieved through socialisation, education and varying degrees of access to the three capital types [25, 30]. Burton explored the importance of cultural capital in relation to crop farmer’s willingness to engage in environmental schemes in the UK [29]. He suggested such schemes do not enable farmers to demonstrate farming skill and the valued cultural symbols (cultural capital) of being a good farmer, this he argued led to lack of adoption by many farmers of such schemes. Other studies have found that other drivers such as economic capital (e.g. having a viable and profitable business) as well as evolving structural landscapes (legislation and subsidy systems) are paramount in changing symbols of good farming. Examples of shifting symbols of good farming are highlighted in a study of conversion to organic farming in the UK [25] and the sheep farming sector of New Zealand [27].

Other studies utilising the concept of the good farmer have focussed on an individual’s agency and the agency- structure relationship that exists in different farming fields. Naylor [20] utilises social identity theory to explore English farmers perceptions towards exotic disease control and suggests that individual and collective identities, together with associated values and norms, have an important influence on animal keepers’ attitudes and behaviours. When researching dairy farmers’ perspectives on antibiotic use on Swedish farms, Fischer et al concentrate on the notion that what farmers say and do is not only influenced by what other farmers think but also fundamentally steered by the political and economic context in which they live [18].

Whilst there are numerous studies in Europe and New Zealand utilising the good farmer framework, there is a notable lack of such work in Sub Saharan African farming systems where research tends to be predominantly single disease focused and quantitative in nature. Of note are two recent publications which adapt the good farmer framework to examine the perceptions and practices of Zambian sheep and goat traders [22] and the risk perceptions and management practices of meat inspectors and slaughter workers in Northern Tanzania [21].

## Materials and methods

### Target population

The primary interest was in local discourse around cattle keeping and milk production in peri-urban systems regardless of farm structure or size. The target population was farmers who keep milk-producing cattle in and around Fatick and Niakhar townships which are within the Fatick administrative region of Senegal. This region lies approximately 150km East of Dakar, the capital city. These areas are classed as peri-urban given their geographical location adjacent to large urban settlements. The region is known to have all three cattle keeping systems seen in Senegal, these being pastoral, agro pastoral and intensive [31].

The sample population was drawn from all farmers keeping one or more milk-producing cows in 11 pre-selected villages. Villages were randomly selected from a data frame compiled for a previous study [11] within the wider ZELS project [32]. The communities in which the study took place adhered to a hierarchical structure, consequently village elders were approached by local project staff to seek permission for the focus groups to take place, and they in turn indicated who to approach and encouraged people to participate in the discussions. Group size was limited to 10 participants or fewer (if 10 were not available at the time) and so recruitment was continued until enough participants in each village were obtained. From previous work in the project it had been established that men were predominantly responsible for the livestock keeping and decision making while women dealt with milk processing [11]. Male participants were recruited to take part in the focus groups for this study while female participants were recruited for focus groups running concurrently to collect data for a study investigating milk borne pathogens [33]. While male and female perspectives were sought on both studies it was decided that the topics were too broad to include in one discussion and due to social norms and power differentials between the sexes separate sex groups would be more appropriate.

### Schedule administration

A pre-designed schedule was piloted with two focus groups and amended accordingly to cover the topics of interest. Questions and prompts in the schedule covered the following topic areas: The relevance of dairy cows and farming, barriers faced in dairy farming, and the perception and utilisation of locally produced milk (see S1 Data and S1 File).

The facilitators who conducted the focus groups were trained by the first author in delivering the schedule and moderating discussions. Three facilitators were recruited locally who were fluent in the local languages (Wolof, Pulaar and Serer). Focus group discussions took place at participants’ houses or in village meeting spaces. Facilitators were not from a veterinary or farming background, all discussions were conducted in the local languages to encourage full participation. Facilitators moderated the discussions in pairs taking it in turn to do introductions and prompt questions. The consent form was read to all participants in their local language before written, individual consent was obtained. To aid discussion around sensitive matters (e.g. discussing numbers of cattle owned which is directly correlated with wealth) participants were encouraged to take part in proportional piling [34] as well as utilising chalk boards to indicate their cattle numbers and milk output on a scale in past years, the present and what they would like to obtain in the future. Ranking of income revenues as well as diseases was used as a discussion starting point, each item was written on an individual board which then could be moved around and ranked in order of importance by participants. Discussions were audio recorded on encrypted devices. An English translator was present and live-translated the discussions to the first author to allow direct input when necessary. When the discussion on each topic came to a natural end, the facilitator invited those who had not spoken or said very little if they had anything further to add before moving onto the next topic in the schedule.

Ethical approval was granted by the Ethics and Welfare Committee of the Royal Veterinary College (RVC) and the Ethics Committee at the Interstate School of Veterinary Science and Medicine of Dakar (EISMV) (URN SR2017-1054).

### Data analysis

The focus groups lasted between 1 hour 15 minutes to 2 hours and 40 minutes resulting in over 16 hours of recordings. These were transcribed verbatim and translated to English by professional translators with no animal science background. All names or identifiable data were removed prior to analysis.

Transcripts were uploaded onto NVivo 12 which was utilised to assist data management and digital coding of all data.

Thematic analysis was employed following the 6 steps outlined by Braun and Clarke [35]. These include:

- Familiarization with data by multiple readings and noting initial ideas
- Generating initial codes by noting interesting features in a systematic way and collating data relevant to each code
- Searching for themes by collating codes into potential themes
- Reviewing themes, checking if the themes work in relation to the coded extracts and entire data set
- Defining and naming themes, iterative analysis to refine the themes and generate clear definitions
- Producing the report, the final analysis stage of selected extracts and their relevance to the research question

Coding was carried out by the first author, codes were initially descriptive at a broader topic level before moving to more interpretive coding through an iterative process of revisiting text and refining codes. The analysis was broadly of a constructionist nature in that we did not focus on motivation or individual psychologies, but looked to theorize the sociocultural contexts, and structural conditions, that enable the individual accounts that were provided [35].

## Results

A total of 11 (7 in Fatick and 4 in Niakhar) focus groups were conducted with 76 farmers (44 from Fatick and 32 from Niakhar) who kept milk-producing cattle throughout May 2017. Although all three (pastoral, agro-pastoral and intensive) farming systems are reported to be present in the study area, the majority of participants identified as practising agro-pastoral farming with 3 participants in two focus groups being purely pastoral farmers. There was a mixture of Wolof, Pulaar and Serer participants, but the majority of groups were conducted predominantly in the Wolof language, with the exception of two groups that were conducted entirely in Serer and one that was conducted in Pulaar. All participants were male, and most had either no formal education or primary-level education only. Most participants were middle aged or older and married (Table 1).

**Table 1:** Socio-demographic data on the participants of focus groups in Niakhar and Fatick in May 2017.

Category	Subcategory	Participants (N = 76)
Age	18–35	9
	More than 35	67
Ethnicity	Wolof	39
	Pulaar	15
	Serer	22
Education	None	49
	Primary	22
	Secondary	5
	Superior	4
	Literacy training	1
	Mosque education	8

All group members participated actively in the discussions. While some groups were from the outset very animated in their discourse, others—particularly younger members of the groups—required some more time and encouragement to share their views.

### Themes

Four themes and associated sub themes were identified during analysis (Table 2). In the first theme ‘Revered cattle’, participants describe how having cows adds to their life in a positive way. The second theme ‘The changing face of livestock keeping’ demonstrates the influences that affect the evolving systems. In the ‘Powerlessness’ theme, feelings of the farmers with regards to the challenges they face as well as the drivers of these feelings are identified. In the final theme ‘Optimism for the future’, consideration of perceptions of individual as well as collective plans and hopes for the future are outlined.

**Table 2:** Interpretive themes developed through thematic analysis of focus groups data.

Main Themes	Sub Themes
Revered cattle	A blessed life
	Cultural identity
	Diversity of cattle use
	Wealth and prosperity
The changing face of livestock keeping	The distinction between traditional and modern farming
	Reliance on agro pastoralism
	Driving production and commercialisation of milk
	The holy grail of dairy cows
	A lack of financial flow
	A need to evolve
Powerlessness	Divine intervention
	A lack of trust in official bodies and organisations
	Fighting a losing battle
	Rose tinted glasses
	The need for external solutions and handouts
Optimism for the future	Seeing is believing
	Feeling empowered
	Visions and wishes for life

### Revered cattle

Areas identified under this theme pertain to the feeling that having cattle enhances a person’s life. Cattle were seen as a blessing which could be used in a variety of ways to bring wealth and prosperity, and which were fundamental to a sense of shared cultural identity.

#### A blessed life

There was a deep sense of reverence amongst the cattle keepers towards their herds and the idea that the cattle are gifted by their creator or left to them by their ancestors, a gift that is immeasurable in its relevance and importance to their lives. Having cattle was seen as a blessing, with participants referring with pity to those who have few or no cattle.

“*There are people who do not have cows*. *There are some who have few cows while they have a large family*. *These people cannot sell cows during hard times*, *the lean season*, *because they simply do not have it*. *There are some who do not even produce milk*. *These people go through difficult situations and they do not receive help*.” *(FG 1)*

This suggests that having cattle offers security, and a buffer for times of hardship or need, which not all in the community have.

Cattle owners not only identified the blessings of their animals on their lives through the elevated status or physical benefits they can bring but also on a more spiritual and emotional level to them as individuals. Talking with a great sense of pride and honour, one participant commented, *“Watching your herd gives great joy and this increases life expectancy” (FG 4)*.

#### Cultural identity

It was evident that livestock keeping was embedded within the cultural identity. Examples from Serer, Pular and Wolof groups illustrated how important cattle were in all significant cultural celebrations and ceremonies and how intrinsically cattle keeping was woven into their identity as belonging to a certain tribe. A strong sense of pride, belonging and commonality were conveyed when discussing how the knowledge and skills of cattle keeping have been passed down through the generations and should be passed down to the next generations:

*“Here*, *you must teach your son animal husbandry because it is our culture and this is what we know*. *Our parents taught us how to cultivate and breed*.” *(FG 11)*

The idea of commonality and a strong community spirit was evident with a lot of agreement that people all faced the same issues together led by their elders and that mutual support and helping others was paramount:

*“Some herds are made up of three cows while others have 10 cows*. *It is not my herd alone but for the community*. *That is why in hard times*, *each brings the little he or she has*. *If you know that you have cows in the herd*, *you will sustain whoever manages the herd because he cares for your cows at the same time as his own*. *This financial support is very normal*.” *(FG 2)*

The community spirit was very much led by the elders who were given much respect as witnessed during discussions, they were served by the younger generations who got seats, water and food for them. Often others referred to them for reassurance or agreement with statements made and it was clear that their approval was sought. The elders from all ethnic groups were local Islamic leaders or Marabouts. Seniority in general, regardless of religious status, is given much respect as in Senegalese culture it is believed that with age comes the privilege and power to be intermediaries between the earthen and spiritual worlds [5]. Within the focus groups therefore it was clear that even when the elders were not the most knowledgeable in the group on livestock matters their opinions trumped all others.

#### Diversity of cattle use

Farmers enthused over the diverse lists of uses of cattle as they discussed their dependence on them across different aspects of their lives. Uses included nutritional, financial, provision of fertiliser and bio gas, as a banking system, as a status symbol, providing employment to herders, and provision of hides, hooves and horns as raw materials, in crafts and in association with cultural traditions such as communication through blowing a cow’s horn and sacrifices during ceremonies.

Much importance was placed on the nutritional value of milk for their own home consumption. It was noted that *“A household that has a herd of cows never suffers hunger” (FG 3)*. Milk was consumed every day, especially in combination with millet or couscous and as a replacement for rice. There was also a notion of milk being superior to other staple food groups in taste and health benefits, especially for the promotion of growth in infants.

*“Fresh milk*, *curdled milk and butter which is at the surface are important to the growth of the baby*. *If you pour fresh milk or curdled milk over couscous or porridge and you give it to children*, *they will finish it even though they did not want to eat*.”*(FG 5)*

The concept of gifting milk to loved ones was discussed as a relevant use and shows that milk is viewed as a high quality product which is worthy of being a gift rather than just an everyday item.

#### Wealth and prosperity

Wealth and prosperity linked to cattle keeping was intrinsically woven through the discussion and conduct of all groups. Amongst the Serer participants in particular, it was clear that discussing numbers of cattle kept by individuals was not socially acceptable, as it is seen as a direct correlation to one’s wealth. This humility in some groups was taken very seriously while in others was treated more light heartedly with participants joking amongst themselves as to who would be ranked higher by size of herd. The view that people perceiving your herd as a sign of wealth was explicitly acknowledged as a bad thing on occasion:

*“if someone sees you graze a herd*, *even if he wants to help you*, *he will not because he will think in his head that the herd represents wealth*” *(FG 1)*.

Others however valued livestock banking as a particular cultural practice with great benefits of easy access to money in times of need such as when you require emergency healthcare yourself or if there is inadequate feed for the herd.

*“Having cows can help you meet up immediate needs*, *because having a cow for the Serer is comparable to having an agricultural bank*. *Instead of going [to the bank/others] to borrow money when you have a very urgent need*, *you go take a cow from the herd*. *You can get a cow from your herd and sell it for 100*,*000 FCFA or 200*,*000 FCFA*^*1*^ *($170-$340) in order to satisfy this need*.” *(FG 3)*

^1^1 USD = 585 FCFA as of June 2017

Participants time and again highlighted the high values that can be gained from the sale of individual cattle as a very positive thing.

*“The sale of a cow can exceed what is earned in [arable farming] for a season*. *The sale of a cow can still ensure the household daily expenses for a year*.” *(FG 7)*

Some participants alluded to this form of ad hoc funds being available to finance education for family members as life changing, allowing students to study all the way to university then in turn caring for their parents with the incomes from the resulting jobs.

### The changing face of livestock keeping

In this theme we investigate the drivers behind why the cattle systems are or are not evolving in certain ways. Participants considered there to be a distinction between traditional and modern farming practices with modern practices concentrating on the production and commercialisation of milk, requiring systems to evolve and utilise breeds with enhanced milk yields. The reliance on agro pastoralism is evident in a landscape where financial flows are often stunted, inhibiting the provision of paid for as opposed to home grown inputs.

#### The distinction between traditional and modern farming

A distinction between traditional and modern farming was evident with traditional farming being associated with larger herd sizes of local breed cattle which participants agreed had lower milk yields. This system focusses on growth of cattle numbers and the herd is where the assets are held. The farmer releases capital when required by selling an animal, essentially utilising the herd as a bank account. The accumulation and upgrading of smaller value animals is an entry point for those starting out, as explained by one participant: *“He did not have cows 5 years ago*. *He only had little goats*. *He sold the goats for a cow” (FG 1)*. Practising traditional farming was sometimes portrayed as a disadvantage compared to modern farming practices, especially when discussing milk yield. *“It is normal for him to have a low production because he practises traditional cattle breeding*.” *(FG 4)*

There was not a clear definition of modern farming although most farmers alluded to modernization or modern practices. The predominant features of modernization were the utilisation of artificial insemination and cross breeding to promote improved characteristics in cattle.

*“With the modernization*, *people are doing crossbreeding in order to increase milk production*. *Production is limited with our local breed*” *(FG 2)*

The concept of intensification and less use of transhumance in modern systems was apparent but not explicit in the descriptions or discussions around modern farming. In general, during discussions many farmers appeared to be striving towards practising modern farming as opposed to remaining in the traditional farming system.

#### Reliance on agro pastoralism

In the majority of focus groups, considerable importance was placed on the idea that arable and livestock farming go hand in hand and that both must be practised simultaneously as has been done for generations.

*“These activities complement each other*. *Animal husbandry complements agriculture [arable farming] and vice versa*. *Agriculture cannot succeed without animal husbandry*, *and animals cannot be well bred without agriculture*. “*(FG 4)*

Taking cattle out to fields to provide fertiliser is seen as a pivotal role of the herd in the traditional farming model and the benefit of having large numbers of cattle to fertilise the soil is seen in higher crop yields. Many farmers suggested that arable farming dominates in their livelihood and feel that the agro pastoralist model as it stands has no reason to change. However, others reported that they have seen big reductions in crop yield or that they have stopped arable practices altogether and see more profitability in animal husbandry activities due to land scarcity (participants often referred to agriculture as crop farming and farming as livestock keeping):

*“People change strategies as land becomes scarce*. *People will replace extensive agriculture with intensive farming*” *(FG 4)*.

#### Driving production and commercialisation of milk

The levels of commercialisation in milk varied amongst participants, ranging from those who did not sell milk at all but rather gave it to their families and neighbours with no monetary transactions, to those who earned a large majority of the income to meet household needs through milk sales:

“*We do not sell milk*. *We only offer it*.

*Offering milk is good*.” *(FG 9)*

*“Milk sales are my main source of income*, *then fattening*, *agriculture and finally cattle sales*.” *(FG 1)*.

Many farmers main priority was to provide milk for their family to consume and only they sold it if there was excess. They viewed it as a useful, occasional, additional income but did not plan household budgets with it as a constant feature.

*“Yes*, *it can earn us money*. *Nevertheless what we produce*, *we prefer to bring it home for our children before selling it*.” *(FG 6)*

The transient nature of profit from milk sales, either as a result of seasonality in production or due to a perceived generalised decrease in production of the recent years, was commonly mentioned.

*“Production often declines and sales do not generate enough income during these periods*. *If we do not have other options*, *we sell an ox at 500*,*000 FCFA ($855) for example to survive*, *especially during the dry season*. *Income from selling milk is enough during the rainy season*.” *(FG 1)*

Due to this fluctuating and at times unreliable income revenue from milk sales farmers often placed more importance on other streams of income such as arable farming. However, a desire to increase production in order to benefit from the commercialisation of milk was a recurring point of discussion especially where participants had seen or heard of others producing higher volumes of milk.

*“With the modernization*, *people are doing crossbreeding in order to increase milk production*. *Production is limited with our local breed*.” *(FG 2)*

Participants alluded to business people who were not from farming backgrounds becoming involved in dairy production with higher yielding crossbred cattle due to the perceived profitability associated with it. Although much focus was on crossbred cattle to increase profitability the idea of changing husbandry practices was also prominent in people’s thoughts:

*“So extensive livestock production may not develop*, *but intensive livestock farming has begun*. *Even us who have the common herds are engaging into intensive livestock production*. *So I think that in the upcoming years*, *animal husbandry is going to expand and if it does*, *trade will also grow since we will sell more milk*.” *(FG 2)*

#### The holy grail of dairy cows

While people were generally reluctant to talk about the number of cattle they owned there was no reserve when discussing those who had exotic breeds or utilised artificial insemination and people freely and openly discussed their desire to acquire such animals and techniques. Imported breeds were viewed very differently from local breeds and were idealised to some extent, with people commenting that they would be happy and problems would be solved were they to have such breeds. Those who did have crossbred cows discussed them proudly, reporting that they manage them differently from the local breed cattle:

*“Many farmers like me already have crossbred cows*. *When these animals will start producing*, *they will be able to give you 7 litres of milk in the morning and 7 litres in the evening*. *A local cow produces 2 litres in the morning and 2 litres in the evening*. *Crossbred cows are not mixed in the same herd with the others*. *I presently have four of them*. *We think that the production will increase in the future*.” *(FG 2)*

Much emphasis was placed on the need and desire for crossbred and exotic breed cattle to solve the difficulties faced by farmers in milk production. However, only one participant mentioned the difficulties having such animals would bring with it:

*“Moreover as people say*, *we need imported breeds to have much milk but the problem is it takes enough money to give them attention*. *When you look at them on picture*, *they look beautiful and well fed*. *However they will have difficulties living here in our warm climate at 40 degrees or 42 degrees in the shade*. *The inseminated dairy cows will begin to suffocate if you leave them in the bush at 10 a*.*m*” *(FG 2)*

#### A lack of financial flow

Much attention was given to the financial burden that cattle keeping places on the farmer. In particular the cost of feed was an area of major concern. Diminishing pastureland and lack of grass during the dry season meant that reliance on purchasing cattle feed had increased in recent times, which coincided with perceived unrealistic price increases in such feed. Participants discussed in detail the cost of feeding and its impact on profitability. Alongside this, some felt that interest rates on loans were prohibitively high while others said that loans were not accessible:

*“Let us assume I have 100 cows*. *I go to the bank to ask for a loan*. *I am refused the loan and the bank grants a loan to [an arable] farmer*. *I have noticed that [an arable] farmer always has guarantees*. *Yet I have never seen someone ask a breeder to secure his cows to be granted a loan*” *(FG 1)*.

Farmers felt frustration that inadequate nutrition was hindering consistent and reliable milk production. They felt that if they could firstly get the means to purchase high quality feed, production and hence profitability would improve allowing them to grow the enterprise and become not only self-sufficient in milk production but profitable.

#### A need to evolve

On occasion, participants suggested that farming practices need to evolve. Suggestions included adapting husbandry practices to optimise the number and timing of calvings, so that milk production peaks coincide with plentiful food supplies, and the need for good nutrition for both bulls and cows in order to have successful mating and pregnancies.

Participants frequently spoke of being victims of land conflict and feeling that the land that should be available to them was being taken away or invaded. In contrast, one participant discussed the responsibility of the farmer to promote sustainable land use and not to accumulate large numbers of cattle which could not be viably sustained. His thought process had stemmed from the interactions he had with the government officials and veterinarians:

*“The state or the government is often blamed [for land degradation] but that should not be the case*. *One day*, *we met the Water and Forests Officer for permission to cut down trees to feed the herd*, *but he clearly told us that this was impossible*. *Animal husbandry should be practised differently because it is a heavy responsibility to feed a large herd of many cattle*. *Trees should attract the rain [and therefore not be cut down]*. *A veterinarian once said to me this*: *“What you Serer call breeding is not cattle breeding*. *You accumulate a lot of oxen and you are unable to feed them*. *It is better to have a few and be able to feed them*”. *Farming the same field for three successive seasons does not prop up the existence of grass on this area” (FG 7)*.

Many envisaged large numbers of traditional cattle being replaced by smaller numbers of more productive cattle in the future. Feeding practices would also need to evolve. For example, having animals penned at home and bringing them feed rather than taking them out for grazing was a recurring discussion point.

### Powerlessness

The sense of farmers being powerless in their current situations is demonstrated, participants believe their lives to be mapped out and controlled by divine intervention giving them little control over their fate. Nostalgic feelings of better times gone by give the sense that during current times they are fighting a losing battle. Many believe that any positive change must come from external solutions and handouts but have little faith or trust in official bodies or organisations with which responsibility lies.

#### Divine intervention

Participants displayed a strong sense of faith. Their deep-rooted affinity for cattle in part stems from the feeling that cattle are gifted to them from their creator, explaining *“We found them here when we came into the world*. *We did not create them*.*” (FG 1*). A strong faith also drives the belief that the future success or failure of their livelihoods is in the hands of God:

*“Change can only come from God*. *If the Lord gives us much rain or excess water*, *then things will improve and there will necessarily be some positive changes*” *(FG 3)*.

Setting objectives or goals was to some extent redundant:

*“When it comes to livestock*, *it is not easy to set objectives*. *One can be in a good condition today and misfortune happens and you lose everything*. *There is no assurance*, *do you understand*? *You can have many cows today and something happens that kills all the cows*. *Only God knows tomorrow*. *Each person knows what he wants to achieve*.” *(FG 1)*

This sense of fatalism may act as a barrier to adopting changes aimed to increase productivity when the outcomes of life are deemed as being entirely in God’s hands.

#### A lack of trust in official bodies and organisations

There was an evident distrust of professional bodies linked to farming inputs and participants felt they were disadvantaged by substandard conditions. Some participants felt that the state-run breed-improvement programme lacked transparency and had not met its objectives:

*“I think a thousand heifers have been imported*. *They have been distributed*, *but we do not know to whom they have been given*. *They were meant for us but we did not see them*. *A breeder who has 50 to 60 cows cannot benefit*. *We do not know if they were given to the people of Dakar or Sangalkam; we do not understand*.” *(FG 1)*

Some farmers displayed a distrust of artificial insemination services as they felt that the product offered to them was of poor quality. There was also some distrust in the advice or skills of veterinarians, particularly private veterinarians as opposed to those working for the public sector. There was a perception that the veterinarians did not want to be out serving farmers so therefore were not reliable or accessible:

*“When [farmers] identify a disease*, *they go in search of drugs to cure the animal by themselves*. *The reason is that if they call the veterinarian*, *he will not come or do what you expect of him*. *When we need him*, *he looks for alternative ways not to attend to the needs of the population*.” *(FG 6)*

There was also a strong distrust of drugs purchased through markets or street vendors. Despite the knowledge that these drugs were often substandard and unreliable most bought them anyway as they were cheaper or because it was the only accessible point to purchase medications.

Some groups discussed a dairy factory local to them. Overall comments about the price paid for milk were negative, although it was acknowledged that the presence of the factory meant there was always demand for milk regardless of the quantity produced:

*“Here is the dairy factory that has been implanted here*. *This initiative is beneficial to them*, *but not to us*. *I sell milk at 600 FCFA a litre*, *but they take it at 350 FCFA*. *They collect milk from us*, *but they do not pay for it until the 5th or 10th of the month*.” *(FG 1)*

The factory was also a disappointment because it was perceived not to have delivered on its promise to reduce cattle feed costs and promote a profitable dairy industry in the area.

*“Of course*, *it decreases our income*, *because they pay at a low price*. *We sell milk at 600 FCFA a litre here at home*. *On the contrary*, *we were told that their implantation [establishment] here would decrease the price of cotton seed as well as the prices of cattle feed*.” *(FG 1)*

#### Fighting a losing battle

Often the mood was one of fighting a losing battle in terms of trying to maintain or improve productivity and profitability in cattle keeping. Lack of access to good quality inputs made participants feel powerless. One of the major limiting factors for farmers was access to grazing land. Two main reasons for this were universally identified as land conflict resulting from urbanisation and construction and lack of grass due to ongoing droughts. There was a sense of the farmer’s land being invaded by the expanding population and at times there seemed to be some rivalry between utilising land for crop cultivation and livestock grazing. Some hypothesised that livestock farming would be better placed to adapt to diminishing land availability than arable farming due to the fact that it can be intensified:

*“The village is expanding; arable land gradually becomes scarce whereas livestock breeding can now take place in a corner of the house*.” *(FG 2)*

Despite the inevitable challenge of the dry season, which many referred to as the lean season, most expressed a desire to be able to continue producing milk throughout the year. The ability to do this however was hindered by access to supplementary feeding materials, with cost often being prohibitive. Water shortage and poor quality salty water were also cited as major limiting factors which farmers had no control over.

Frustration at the lack of access to veterinary inputs was evident and many felt that, despite their best efforts to treat their cattle, they were helpless as they did not have the necessary skills, knowledge or access to professional expertise:

“Sometimes we may have an emergency with a cow, but we have no one to attend to it. In such case, we are forced to use our local practices to try to heal the animal.” (FG 4)

#### Rose tinted glasses

A sense of nostalgia for better times gone by and a feeling that things were easier in the past was evident. A prevailing factor was the perceived abundance of grazing land and plentiful grass of past times, which had enabled them to produce more milk from their cattle.

*“Grass is lacking*, *because the tops of all houses were made of herbs during harvests in the past*, *but that is no longer the case nowadays*. *The drought of these last years prevented the development of animal husbandry*. *I did not live in the other periods*, *but I have found these last years that lack of grass is an obstacle to the good practice of breeding*.” *(FG 7)*

Many spoke of cultivating land in the past but having stopped, largely due to recent drought conditions rather than by choice. Some explained that artificial insemination had been provided free of charge in the past, which, they felt had been of great benefit, but now that they had to pay for it, it was cost prohibitive.

#### The need for external solutions and handouts

Thus people proposed the idea that they could not do anything to improve their situation but that external bodies were required to solve the problems. Funding, subsidies and handouts from foreign partners and NGOs were frequently suggested as potential solutions to current challenges. Responsibility lay with external sources of power or influence to solve the problems faced by farmers, predominantly finance and access to improved breeds at minimal costs, were suggested as a necessity. Influence of the state or the rural council were viewed as important to change policy in regard to land scarcity and cattle feed prices as well as to enable access to good quality veterinary care by farmers. One participant who had taken part in the state-run breed improvement programme discussed the need for training in order to change husbandry practices, highlighting the attention that needs to be given to planning mating and nutrition requirements of a herd:

*“Also there is a need to master [optimum] pregnancy [management] in the milk production*, *because if unfortunately a [cow] that has to give birth this year is not mated*, *it will not produce milk*. *If it does not eat well too*, *it cannot be mated*. *Likewise*, *if the cow is pregnant and does not have enough to eat*, *it can have an abortion*. *That’s why it is necessary to get the training and the means in order to enable cows and bulls [to mate successfully]*. *This is why bulls must be well taken care of*, *even if it is necessary to tie them elsewhere and feed them alone*. *Heifers must have a good diet*.” *(FG 2)*

This focus group participant was the only one to raise explicitly the idea of self-help changes to promote productivity and profitability at the farm level while others concentrated on broader environmental and policy changes to affect change.

### Optimism for the future

The final theme investigates how farmers feel with regard to the future of farming in Senegal, the drivers of their beliefs are explored in the first two sub themes: Seeing is believing and Feeling empowered before the final sub theme Visions and wishes for life concludes what visions farmers have for the future of farming.

#### Seeing is believing

Despite the difficulties raised, there was an air of optimism about the future of cattle farming, stemming either from positive personal experience or from witnessing others’ successes:

*“However we thought that the importance of cattle breeding in our village would decrease these days*, *especially with the difficult conditions caused by the climate change that has caused lack of fodder*. *We thought that the livestock industry will decrease here*, *but it is the contrary*. *An increase in this activity has been noted because it is profitable*.” *(FG 3)*

Participants often evaluated other people’s experiences and were inspired to follow suit when they witnessed successful outcomes:

*“As we no longer have space for grazing*, *we can use other strategies as others successfully did*. *For example in Louga*, *cattle owners build pens in front of their houses in order to tie the animals and feed them on the spot*.”*(FG 3)*

*“You earlier mentioned someone who harvests many litres of milk per day*. *This happens because he owns crossbred cows due to the artificial insemination he has done*. *Therefore we will be able to produce much milk if we can get them too*. *We would really like to follow his example*.” *(FG 8)*

Despite the general feeling that things were easier or better in times gone by, many people acknowledged tangible positive changes over time. Reduced mortality of cattle, attributed to successful vaccination campaigns and animal health provision were seen as a major positive:

*“There is an improvement in the conditions compared with before because there was a high rate of livestock mortality due to diseases*. *This has clearly disappeared now*.” *(FG 1)*

*“These changes are due to the practice of cow insemination and the improvement of animal health thanks to the intervention of veterinarians and animal vaccination campaigns in the locality*. *There are a lot of diseases that have disappeared thanks to the efficiency of drugs and also the availability of cattle feed*.” *(FG 5)*

#### Feeling empowered

Despite the many expressions of feeling powerless, many farmers spoke with a sense of empowerment. Defined circumstances had led to these feelings of empowerment such as specific successful programmes that had been implemented and gave farmers optimism about the future:

*“However all warehouses are now full*, *thanks [to] cooperatives and BNDE (Banque Nationale pour le Developpment Economique)*. *That is why cattle feed is now available and cattle vaccination campaigns are efficient*. *These combined factors can boost the [livestock] activity*.” *(FG 3)*

*“Indeed*, *we are certain that there will be change because we now benefit from the support of the state with regard to the access to inseminated cows which produce much more milk*.” *(FG 5)*

One participant noted how certain breeders have accumulated their own ‘toolkits’ of medications on hand and expressed that having agency and the resources to treat their own cattle rather than be reliant on the veterinarian often made farmers feel empowered and how it enhanced the productivity of the business:

*“Every breeder has his vaccine [medicine] toolkit and he can directly care for his animals without the assistance of the veterinarian*. *This promotes the reproduction of species*.” *(FG 3)*

Conversely, others did not feel empowered by this situation, being unsure of the correct treatments and dosages to administer by themselves and not trusting the efficacy of drugs purchased through informal markets.

#### Visions and wishes for life

Overall most participants valued highly the production of milk and a general desire to continue with milk production in the future was evident despite the challenges faced. Many participants felt that their children now had more choices in life and might pursue schooling and other careers instead of following the farming tradition. There were conflicting opinions on this within households:

*“Their mothers also have their words to say*. *Often they do not want their children to get into our activity because the income earned from cattle breeding and agriculture is not permanent*.” *(FG 2)*

There was hope that cattle feed prices would reduce and access to veterinary intervention would improve in the future:

*“We would like to have competent veterinarians and benefit from a considerable drop in the price of cattle feed which is presently expensive*. *If all these conditions are met*, *we will be more motivated to produce*” *(FG 4)*

In all groups much emphasis was given to a vision of acquiring improved high yielding cattle in the future which would change their practices and improve profitability:

*“A hundred traditional cows will be replaced by 20 improved breed cows producing more milk*.”

*“We would like to produce the maximum possible*. *For example*, *we would like to produce 100 litres or more with 10 cows*.” *(FG 4)*

No participants mentioned leaving farming or pursuing other areas in their future instead all wanted to increase in cattle numbers or production of milk generally.

## Discussion

In this study, cattle keepers provided useful insight into the current perceptions they have around milk production and livestock keeping in two targeted areas involved in peri-urban farming in Senegal. A deep-rooted affinity with cattle and a strong sense of identity and pride meant that participants were happy to discuss the issues as they viewed them. The strong traditions and cultural identity associated with cattle keeping however do not seem to impede an ongoing transition in farming practices towards evolved systems focussing on higher productivity and profitability. While there is still a strong sense of community and commonality amongst farmers who are led and influenced by elders, a shift from community herds and shared decision making to more individually driven enterprises is evident.

Analysing the Senegalese farmers’ views on livestock farming through the lens of being a good farmer, the present study indicates that what it means to be a good farmer has changed with the growth and intensification of the dairy sector. In Senegal, previously a good farmer was judged as someone with a large herd size whereas now increasing value is given to individual cows with high productivity markers, individual metrics of milk yield are judged and the use of technologies such as artificial insemination and skill of cross breeding are valued as symbols of a good farmer. These symbols were often associated with what people termed modern farming, but a clear definition of modern farming was not explicit and a sliding scale of practices seems more appropriate than a binary categorisation between modern and traditional farming in this setting. In line with Naylor’s study of British farmers [20], our participants categorised themselves within groups, e.g. those practising only traditional methods and those employing artificial insemination. The biggest distinction of another group with which they did not identify were those ‘business’ people who entered intensive farming lured by its profitability but who they did not view as real farmers. Shortall [28] highlights the fact that while the cultural capital inherent in good farming leads to a degree of inertia, when farmers are challenged in some way (particularly if practices are no longer profitable), they will change their activities and renegotiate associated good farming standards [25]. In our study many farmers identified that the cultural capital gained by owning large herds holds less prestige in current times than the cultural capital of having fewer but much more productive animals, this in part is driven by the structural confines of reducing availability of grazing lands as well as the economic capital which is associated with milk sales. While Burton [29] argued that farmers willingness to adapt farming practice was reliant on their ability to retain cultural capital by means of displaying symbols of good farming, Sutherland argued that changing rules of the game and a farmers’ ability to retain or increase economic capital was more important [25]. Our participants believed that inherent difficulties could be eased if they could access appropriately fair financial loans and agreements in order to provide the necessary inputs to make their enterprises more reliably profitable. However, banking and finance institutions will not form agreements on livestock as collateral, given the vulnerable nature of the asset. Major structural drivers have proven to be of greatest influence on farmers behaviours and recognition of good farming symbols in both the UK and New Zealand where legislation and subsidy systems moulded farming practice [27, 29]. They included financial support for rural communities, free-trade arrangements, and productivity as good farming targets during the productivist era [24, 27]. In the post productivist era, symbols of high productivity have been devalued as the importance of environmentalism and animal welfare have gained prominence [27]. In Senegal, capital flows may be influenced by the country’s productivity agenda as well as international investment in livestock sectors through bodies such as the International Finance Corporation (IFC) and the European Bank for Reconstruction and Development (EBRD) [36]. Microfinancing alongside innovative farming techniques, access to vaccines and market development have proven successful in programmes such as those delivered by the NGO Brac [37]. Improving profitability of dairy enterprises by addressing access to and affordability of inputs, access to finance and insurance for catastrophic events through co-operatives and mutually beneficial farmers unions, and by establishing viable value chains, would facilitate a landscape that was amenable to disease control and surveillance as well as providing the necessary economic growth and development of self-sufficiency for which the government is striving.

It was evident that study participants were driven to action by seeing others achieve success. This is a widespread phenomenon in farming, described through the theory of symbolic interactionism where symbols of good farming are used to transfer information between farmers [29] and how people viewed as being within one’s own group are far more powerful than those viewed as outside the group [20]. There are countless examples of the use of peer support in human healthcare settings for health promotion focussing on diffusion of information and positive encouragement [38]. Natural lay helpers are commonly described and utilised in many settings as individuals to whom others naturally turn for advice, emotional counselling and tangible aid [39]. In our study setting there are very strong social networks and established hierarchical systems with obvious natural helpers in the elders and established farmers. This is a valuable asset that can be utilised for effective uptake of healthcare initiatives, incorporating these people in service delivery through targeted training and incentives to recruit or educate others would be a viable way to ensure appropriate penetration of services to all and potentially to facilitate a more successful uptake and utilisation of programmes. While the elders are not always the most experienced cattle keepers in the vicinity their influence and power is substantial so incorporating them is paramount.

The role of the veterinarian is highlighted often for the importance of a strong relationship between the veterinarian and farmer [20, 25–27]. Veterinarians can be seen as both within the farmer group and outside and therefore as an important channel for disseminating information and practices to farmers. In some systems studied, value is placed on the farmer’s ability to judge the stock by eye and be intuitive about the health of animals, knowing how to treat them independently and only calling a vet for extreme cases. Conversely in many intensified systems, a symbol of being a good farmer is regular and close contact with a veterinarian or other agricultural consultants [20, 25, 26, 28]. These views were echoed in our data, with some farmers taking pride in being able to autonomously treat their own livestock when the need arose while others craved more access to veterinary intervention and advice, either through a perception that professional help would be better or that they were not adequately experienced or trained themselves to treat sick animals.

### Policy implications

From the study findings, three distinct areas are important when considering the planning and provision of cattle healthcare. These being the differentiation in groups of farmers, e.g. traditional farmers, traditional farmers with modern practices, and new business farmers; the knowledge on shifts in social and cultural capital and the information of persons of influence. Intensive well financed farmers for example may respond to quality assurance schemes to comply with disease control, where they are afforded access to profitable markets for assured hazard free products. Farmers expanding their modern practices may be motivated by access to funding, technologies and training and entry points for both surveillance and healthcare delivery through such interventions would be advantageous. The farmers who identify themselves as traditional value being able to graze substantial herds and require affordable and accessible services even when cattle are on transhumance as well as access to adequate grazing lands.

References to successful vaccination programmes and people’s desire to access appropriate healthcare for their cattle illustrated general recognition of the positive impact of disease control and healthcare measures, suggesting that there would be uptake of accessible services. While the availability of fully qualified veterinarians is problematic in this system, access to other paraprofessionals may be more achievable. Local pharmacies and artificial insemination technicians are more readily available in the vicinity of the farmers and therefore training these people and employing them in an outreach system would be a viable avenue for delivery of routine healthcare services. Farmers expressed that it is the responsibility of influential powers such as state and government bodies and professionals in the veterinary field to make change and improvement in terms of veterinary provision and general legislation around markets and land. However, these groups are easily distrusted by the farmers who readily point out shortfalls in current and previous interventions and provisions. Building a stronger communication channel through local leaders or association representatives being included in iterative planning and assessment schedules may foster a greater trust as well as an avenue to make needs and concerns heard.

Etiquette surrounding the practice of not disclosing the number of cattle one has and the ad hoc selling of cattle when money is required make surveillance and disease monitoring more difficult to plan. Utilising the trusted natural helpers here also could ease this problem. These people are part of the community and have a strong grasp on individual circumstances in terms of cattle numbers and behaviours around selling and buying practices as well as transhumance activities. If this knowledge is sought and these people are engaged in the planning stages they can effectively communicate the needs of their local community.

### Methodological reflections

This study was limited to a particular geographical area with systems and farming conditions that differ from other areas of the country. As such results cannot be generalised to all Senegalese farmers. The aims of the study were to understand farmers’ perceptions and behaviours to appropriately contextualise the setting with a view to how this may affect the implementation of disease control and healthcare delivery. The first author of the study has a veterinary background and as such will naturally view things with a preconceived lens when interpreting farmers’ discussions. Free discussion amongst participants was encouraged but in reality the responses and direction of conversation were potentially influenced by the framing of the questions and the order of prompts used by facilitators, who became familiar with topics and responses as they carried out more groups and at times began to offer more specific prompts than had originally been included. It was originally envisaged that although the prompts were written by the first author, the execution was by facilitators with no veterinary or animal science training so bias in questioning would be minimised to some extent. In future work more explicit training on not allowing influence of previous groups’ answers to direct facilitators in subsequent groups should be incorporated and monitored. This particular limitation is one which this represents an issue commonly faced by researchers when considering the questions asked and what responses they either open up or close down [40]. Despite setting up the groups to make participants feel at ease as far as possible, the presence of the first author (an outsider who required live translation throughout the groups) cannot be overlooked for its impact on participants. This perceived power dynamic may have influenced the many references to ‘researchers’ and ‘westerners’ offering assistance, which may reflect a perception that NGOs and western organisations have previously offered free treatments or livestock gifting programmes, for example. The perceived power dynamic from an outsider being present would certainly influence the way participants talked about what they needed, believing that an outsider may have an impact on securing change at a higher level. Frequently when asked if they had any questions for the research team, participants asked what help we could provide them and if we could offer financial assistance or artificial insemination services. They may have believed that the purpose of the study was to investigate avenues for charity and felt they had to sell their cause. Following the focus groups and other wider project activities, feedback sessions were organised to discuss results and outcomes with the participants to allow them to ask further questions and discuss the next stages in terms of disease control in their areas. This will hopefully help alleviate misunderstanding in the future about the objectives and necessity for research work.

Other than separating male and female participants and encouraging quieter participants, no other consideration of power dynamics within groups was given [15]. For instance younger members of the groups were more likely to be quieter and less willing to voice their opinions or disagree with elders. This follows the hierarchical and respectful nature witnessed. It is feasible that the younger generations (who indeed will have most influence over future directions of farming) have differing views and aspirations and if they had been separated from older generations may have shared different insights.

Data gathering through focus groups is a valuable tool that, particiapnts are often more at ease in a group asthey do not feel they are responsible to give all-encompassing or correct answers as they might feel pressured to do in a one-to-one interview. This methodalso provides rich data when people elaborate or disagree with others’ ideas. In our circumstance it generated discussion on what the wider farming community would do, as well as capturing reflections of individual farmers. Overall, a qualitative approach allows a deeper understanding of people’s perceptions and beliefs and how these influence behaviours and routines, which is important when considering interventions such as disease control, which rely heavily on individual and group behaviour for success.

## Conclusion

This study highlights the importance and reverence that is given to cattle amongst Senegalese farmers and illustrates the relevance of traditional beliefs and customs when it comes to cattle keeping despite the evidence of a shift driven in part by commercialisation of milk products. There are many frustrations around the challenges faced by farmers, which are currently hindering the development of the sector. However, despite these frustrations farmers are optimistic and hopeful about continuing and growing their milk production in years to come.

In this setting there is a gradually altering balance between traditional and modern farming practices, this is likely to result in a very heterogeneous landscape of dairy farms. Therefore, interventions and activities regarding disease control may need to be targeted appropriately to meet this heterogeneity. There are many complexities of this system such as fluctuating cattle numbers in a given area and none traceable animals which will pose a challenge for appropriate and adequate healthcare delivery. However, there are also elements such as the social structures and norms which, if understood and considered carefully during planning stages, will aid the appropriate dissemination and uptake of initiatives and programmes.

## Supporting information

S1 Data (ZIP)

S1 File Senegal focus groups-male farmers. (PDF)
