# Supplementary material for: “Everything in this world has been given to us from cows”, a qualitative study on farmers’ perceptions of keeping dairy cattle in Senegal and implications for disease control and healthcare delivery
Source: PLoS One. 2021 Feb 25;16(2):e0247644. doi: 10.1371/journal.pone.0247644 (PMC7906343; doi:10.1371/journal.pone.0247644)
Supplement: S1 Data — (ZIP) [file pone.0247644.s001.zip › Data/17501 FP1 English final.docx]

**File** **17501** **FP1**

**INTRODUCTION IN ENGLISH (00-03 seconds)**

**Introductions have already been made.**

**Have we taken pictures of the decor?**

**Pictures?** **We will take them later.** **We will later take pictures of those who participated** **in** **the discussion. When she will go back, she will tell her administration  the time she came to Senegal,** **this person**  **helped her,** **that one was a shepherd, the other person interpreted her and so on.**

Tell her that   when she returns to England and gets appointed as a minister or a senior official, her position should not make her forget that she had promised to help Senegal . Let her do everything not to forget it.

52

You just have to write 1952!

No, he says he is 52 years old.

Ok.

That one is 61 years old.

**What we would like to discuss today is what can make your work improve.** **You breeders and you working in the milk sector,** **your opinions and that of others are of great** **interest to** **us** **because each person needs to competent in his** **field.** **You** **already have some experience in the field of livestock breeding and milk production.** **Let** **everyone give his/her opinion that can be profitable to his neighbor as well as to others.** **These** **are professionals in the field. They will use what you will tell them to write a PhD thesis** **for the** **interest** **of the whole world,** **because Whites naturally love sharing their knowledge.** **That is why we ask you to share your experiences.** **Everything we say here** **will remain secret** **and will not go** **to** **Niakhar or** **Ndiaye**-Nd**iaye.** **All** **that will be said will stay** **here.** **It** **will be a secret among us who participate** **in** **the discussion.** **Nevertheless we will keep a copy of the discussion, because** **this lady will write in the** **interests** **of the whole world.** **She urges** **everyone** **to** **partake** **in** **the discussion.** **Besides,** **she will record the conversation** **and** **afterwards she will take memory pictures.** **She** **also** **said** **she will assign each of you a number in place of your names. So when transcribing, all your names** **will be** **erased**. **Afterwards the data will be stored** **for** **whosoever in the public wants to get access to.**

**Now, the** **first** **question requires** **everyone to say whatever he knows about ...**

**Ah, good. We can proceed with the** **first** **question.**

Do a little faster, Manou. We are waiting for you.

**Sign here.**

**Basse,** **go on!**

**How old are you?**

52

1952

I am of the same age with that one.

He was born in 1952.

**Are you** **educated?**

I attended the Koranic school.

**Now we want to talk about the importance of milk in the first place.** **We want to ask you**   **if selling milk is your main source of** **income**. **Does what** **you spend come from the profits from the sale of milk?**  **Let us start with the cows first.**

Does everyone have the right to answer or not?

Everyone must tell the number of cows he has.

We had already answered this question before. We were already investigated on that.

**Ok,** **I do** **not** **have** **this questionnaire; that is** **why.**

**Now, let us proceed individually.**

-Group discussion

- That is it. That is what I told you.

**What is the value of milk in relation** **to** **your income?** **Everyone can give his opinion.**

I think that all our wealth depends on our cows, and it is the cow that gives us milk . You cannot get milk without possessing a cow. Cows represent everything for us. We found them here when we came into the world. We did not create them. Their creator, we do not even know him because we have never seen him.

**Have you been brought up** **in the midst of cows?**

We found cows here. We grew in their company. It is a legacy from the ancestors. Everything in this world has been given to us from cows, even  milk. Our daily expenses, our buildings and even our plane ticket to Mecca come from the cows. The sale of milk cannot alone buy a ticket to Mecca. If you sell cattle, you can have enough money for that. The sale of milk allows us to meet our expenses for food. From time in time it also helps us to buy feed for livestock. Currently, each cow consumes an average of 1,000 Fcfa in food daily (livestock feed, peanut cake, cotton seed and so on) for each dairy cow. These expenses are still part of the obligations. Livestock breeding in Fatick area is a problem due to the quality of the water which is salty. There is less and less grazing area due at urbanization. Carters principally dig the earth to collect sand for construction. Unfortunately, this prevents grass from growing as well.

**So selling milk helps you to meet** **the expenditure related** **the food of the household and cow feeding.**

Provided there is sufficient production. Presently the production is not sufficient.

**I** **understand.**

Presently there is not enough grass . Cows do not produce more than one litre or less. In the rainy season, the sale of milk can meet these needs. Now the revenue from the sale of milk is exclusively reserved  to  livestock feed and sometimes we are even obliged to complete it.

**Now as the sale of milk cannot meet** **these expenses**, **where does the money come from to** **supplement** **these expenses?**

From cows. We sell an ox from time to time . The revenues will be used for expenses. We sometimes sell a sheep.

**What the gentleman just said is** **very** **important. I** **understand** **perfectly because I am in the milieu like you now. Does someone else have** **some information** **to** **add?**

To agree with my colleague , I will add that it is not just that in the field of extensive breeding that we evolve . We also do cattle fattening apart from selling milk. Although the market is sometimes tough, especially in this period, we would much appreciate if we could have access to funding at low interest rates. Cattle fattening requires substantial financial resources. Livestock feed affects the value of the product. Loan interest rates are high.

**Thank you**. I**s there another contribution?**

Yes. If we could help during the lean season , we would be able to provide refundable livestock feed during the rainy season .

**But** **aside from the proceeds from the sale of milk, what is your alternative source of income?**

In addition to milk, during the breeding season we sell cows to provide to family and livestock feeding . That is what we do.

**So is the sale of animals** **your source of income?**

Indeed, the sale of milk cannot meet our annual needs. It just covers some expenses.

**Do you presently get any assistance?**

At this moment ?

**Yes, from your family or loved ones?**

No.

You know the breeder is different from other workers because if someone sees you graze a herd, even if he wants to help you, he will not because he will think in his head that the herd represents wealth. He will deduce that you should not need help. I myself have a son who receives more than 400,000 Fcfa a month. Despite everything, he does not support me. He only looks after his family and his business. On the contrary, I support him because among us the Fulani when your son gets married, the father father offers an ox . Although he is a civil servant, that is the custom among us.

**Is there another contribution?**

There are people who do not have cows. There are some who have few cows while they have a large family .   These people cannot sell cows during hard times,the lean season (rainy season with less food?) because they simply do not have it.   There are some who do not even produce milk.   These people go through difficult situations and they do not receive help.

**Now where does their income come from?**

They put everything in the hands of God . They cannot benefit from loans because they do not have collateral.

**In short, we would like to know the impact of the sale of milk on** **your financial activities** **as well as if you have other sources of income.** **When analyzing** **your answers,** **you told us that the livestock sales constitute** **your other source of income.** **Therefore your wealth comes from** **livestock.** **So we will move on** **to** **another** **question.** **By order of importance, what are your sources of income?**

I have previously answered you that selling milk often helps us meet our needs. Production often declines and sales do not generate enough income during these periods. If we do not have other options, we sell an ox at 500,000 Fcfa for example to survive, especially during the dry season . Income from selling milk is enough during the rainy season.

**If there is milk, it is enough to meet your needs. Do you turn to other things in case there is no milk?**

In case it is not in “Djoloff”. You can survive here if you have milk, but you cannot if it is in Djollof. You must sell a lot in order to buy rice, oil and food. Cows balance the rest.

**Do you cultivate?**

Yes, we used to cultivate in the past. However we no more farm because of birds that destroy crops .

I grew millet during the last rainy season, but my production was insufficient.

In Djoloff, people grow watermelon.

Here in Fatick, I used to farm, but  I no more have land to cultivate nowadays.

Now we are only breeding and selling for the most part. Animal husbandry and trade are our main activities.

**I think it is okay. What they outlined is what we have here.**

**Now what is your** **main source of income?** **For** **example, if we** **take** **these** **different** **piles of** **pebbles, what does the milk sales, livestock sales, fattening** **or agriculture represent in relation to the differences in weight** **of piles of** **stones?**

**That is it, “Téphanqué” that means trade.**

**Idi, someone can sell cattle without being a merchant. This is not the same with someone involved in commercial activities.**

Here I will put trade or the cattle trade of cows, sheep and goats.

**Yes, it is not the same with someone who sells an animal to meet his needs.**

Here I put goats and cows.

**We said that you should indicate your income earned from selling milk, your income earned from selling cows, and the income you get from selling at** **Diakhaw market.**

**I** **saw him trading** **in** **Diakhaw.**

All of us will position them on milk.

**Yes, everyone will place them.**

As for me, I trade the most.

**So I put 1, 2, 3, 4, 5, ok 5. It is well.**

**What follows?**

It is necessary to put 6 for cows.

**What is next?**

The sale of milk comes next.

**So I put 4.**

**What comes next again?**

The next is to take my flock and sell it.

**This is the following.**

You add 1 everywhere and you continue.

**These three?**

As for me, I want milk but I do not have cows. So, do not ask me.

He said he does not cultivate.

Yet I grow plants.

**Okay, wait until we come. Take your turn.**

**Mr.** **BA**   : In order of importance fattening, then milk sales and finally livestock sales. I would have liked to cultivate, but I have no land.

Out of the income you earn from selling milk, the income you earn from selling cows and the income earn from the products you sell at the market, what helps you the most in household expenses?

**Mr.** **Faye**   : Milk sales are my main source of income, then fattening, agriculture and finally cattle sales.

**(*Section* in English) 32 min – 34 min 35 sec**

**What** **kinds of crops** **do you plant?**

Millet.

**Do you grow** **peanuts?**

No. There is only millet that later I mix with milk.

**She does not understand the French**, **the Fulani or the Wolof languages. She only understands English. That is** **why** **we translate for** **her to better** **understand** **your** **problems.**

**Do you cultivate?**

Yes, I cultivate.

**What is your main source of income?** **Make** **your choice** **among the piles of pebbles.**

You know, I combine agriculture with animal husbandry. I f I could, my animals would not migrate up to Tambacounda because it is far away and we walk on foot up to Tambacounda. I wish I could have lands in the immediate environment to have fodder to feed my animals.

**He wishes he could have land for** **fodder, because** **he travels more than 500** **kilometers to take cattle for transhumance.**

**Now what is your main source of income?**

There is the sale of milk in the first place,   then fattening , farming and cattle sales. You see this hay the cows are eating,  we fetch it at five kilometers from here. If the trip is added to, it makes 10 kilometers in total . You know it is difficult . Whites have techniques to grow fresh grass in few weeks. If we could have access to these techniques, we will gain a lot. We live terrible adventures during transhumance . One day I almost died of cold in the rain; my children and I were all wet. If we could be provided tents, we would avoid these kinds of inconveniences in our journeys. Unfortunately breeders are respected  here; that is shocking.

**Yes, I** **understand** **he is expressing his grievances, but** **these** **are researchers.** **Maybe** **they can put you in touch with NGOs that can act on these** **problems.**

**Do you** **think these** **problems** **will disappear** **one day?**

Yes. There is an improvement in the conditions than before because there was a high rate of livestock mortality due to diseases. This has clearly disappeared now.

**Do you plan** **to increase your breeding activity or** **are you going to** **decrease this activity?**

Never in my life . We only have livestock breeding. If I had the means, I would raise more than 2,000 heads .

Unfortunately our activity will disappear because our sons are all going to school nowadays.

**That is** **right.** **We will come back to this issue in a short** **while.**

Nevertheless, BASSE or you should inform us of your arrival so that we can prepare accordingly for you to have reliable data, should they?

**That's** **right.** **Nevertheless,** **the data we take** **from you will be reliable as well.**

I'm sorry to interrupt you. I just wanted to say that our whole life depends on cows. Now cattle have problems because you can see a physically  healthy cow at the beginning of rain, but it dies after eating fresh grass because of plastic waste. We are fed up.

**Here** **where** **you** **are, how many cows does the person who** **has cows the most have?** **Consider all the Fulani who are here.**

Concerning the person who has the highest number of cows, I dare not say that such a person as such other person is the one. However I know that ...

**No,** **do** **not** **give** **names. Rather for such a person who has the highest number of cows, how many cow**s **does he have?**

Where? Where?

**Where** **you are sitting there, in the entire village.**

That is to say, he is asking how many cows the person who possesses the most cows in our village has.

Serer or Pulaar?

All of us.

Pa Demba Calo.

No. Search for them in the notebook. You need to look there.

**Idi,** **have** **you** **read the questionnaire completely?**

Yes.

**Can you not map it?** **Map it** **… No,** **no,** **no ...You take the slate… Take the slate… The slate can present the scheme.**

Faye,  you have to ask the veterinarian. We cannot talk about our cows individually.

**No, I do not ask for a name.**

No, I know you do not ask for a name, but he knows. Whoever among us who speaks will not tell the truth and God wants the truth, so if you go to the veterinarian, he will tell you.

We are sitting here. If it is the truth you want, Demba is here. Demba is our elder brother.

You know in the beginning El Hadj Demba , we said we will not talk about anybody's name here.

**That is it.**

**Next time if it happens you draw a line like that, for example, we will say that the one who has fewer cows is the one who is down and the one who has more cows is the one at the top.** If we say that the most largest herd is 100 cows and **someone**another one that has a cow, and if it was a path where **101** cows are passing through there are 101 cows, where do you **get** positioned yourself between the two.**Hope you understand what I want to tell you.**

**Yes, I have understood.**

**Have you come in the middle or not? And if you divide them into four parts, then you will know if El Hadj Demba is effectively in the middle or at the top.**

**You know if he is here, he will say that he is nowhere . And know that no one will tell you he has so many cows, be it a Serer or another breeder.**

**That's why I told him to tell me approximately, that is just why. That is to say, if we know someone who has fewer cows and someone who has more cows, each person  will know where he is positioned on the line. It is not meant for us because we all know that no one should claim it. Nonetheless these people want to get that information for their calculation. It is done the way BASSE did it.**

**That is why Idi told him to go to the veterinarian in order to know who has the highest number of cows. That is not the question. Rather for you who are here, tell us so that we may know.**

**So El Hadj Demba, who is the person who has the least number of cows?**

Ha no. It is me, me.

**No, but you know we do not give names.**

This, I am the one who just has a cow.

**Then wait. I have taken from 1 to 100 cows. Now where are you positioned in relation to this interval?**

Draw a line. I would give everyone to do the same thing.

Place me 5.

So I put 5.

The next… draw a line.

No. Let him do it. If he draws 1, it is 1.

**Let him do it.**

I place it here.

**Give to the other one.** **He is the only one left.**

I have 3.

Great. That is it.

**Had it been students, you would have known who the first, the** **second** **and the** **third are.** **Even here you know who the first one is too.** **You must look at** **our faces** **and you will know.**

**Has someone not done it yet**   **?**

If everyone has ticked, we should be six people.

**So here is the other question: what objectives do you expect to achieve in the next** **5** **years** **?**

When it comes to livestock, it is not easy to set objectives. One can be in good condition today and a misfortune happens and you lose everything. There is no assurance. Do you understand? You can have many cows today and something happens that kills all the cows.

We are fed up of two things  : it does not rain and plastic bags. We are tired of two things.

This season is very bad for a pregnant cow because it can put to birth and die with the calf.

**The question asks you to look at the cows you had the past** **5 years, the ones you have today and those you dream to have tomorrow**.  **Only God knows tomorrow. Each person knows what he wants to achieve.**

They have increase from the past 5 years till today.

However he lies, he has more than that.

Well, let me give this example. He was there. He is presently here.  He had been there for the past 5 years. So he has decreased from where he was. He was at this level the past 5 years too, but he has declined too.

**Now, tick** **for the past five years** **whether it has** **decreased** **or** **increased.**

So he was there 5 years ago, but he is here today.

He did not have cows 5 years ago. He only had little goats. He sold the goats for a cow.

**Now only milk production is left.** **I'm going to ask where milk produces more**   **and where it produces less. I mean the milk you get from cows**

Cows get more milk during the rainy season. You see, there was no milk last month and there will be nothing until rain comes.

In fact he is asking the quantity of milk that you daily produce in the peak season. In case there is really milk, how much does a cow produce?

When there is, each cow can produce two litres in the morning and two litres in the evening. Our cows do not produce much milk.

It depends on the cows . Some may produce 1 litre, 5 litres or 3 litres  during the rainy season.

That is to say, milking is stronger in the month of September and milking is low in the months of May, June and July.

**At this time, how many litres of milk do** **cows produce** **?**

The cow that used to produce 2 litres now gives 1 litre. The one that milked 1 litre gives half a litre.

**At first**, **this phenomenon did not exist. What caused it** **?**

The reason is there is no more food for the cattle. This change has been brought by the absence of rain.

**Do you think this phenomenon can change in the future and come back to normal as before** **?**

In my opinion, it will not change . To increase milk production , it is necessary that this new race of breeds be distributed to each farmer. At least two cows are needed to increase milk production. This cannot happen if we only depend on our local breeds. There is presently no more forest. Grazing lands are scarce and people have invaded the land.

**Will you allow your children to get involved in animal husbandry and agriculture?**

Yes, if you wish to cultivate, you will. If you want to breed animals as well, you will. You can combine animal husbandry and farming. Concerning agriculture, we no more cultivate due to lack of rain; if you grow a plant, it will not reach maturity.

**He is asking if you are going to encourage your children to cultivate and breed.**

Some will cultivate, others will breed animals. Some will drive carts and others will learn to drive motorcycles. They work anyway.

**Now which difficulties do you encounter** **?**

What people did here is a problem. Here is the dairy factory that has been implanted here. This initiative is beneficial to them, but not to us. I sell milk at 600 Fcfa a litre, but they take it at 350 Fcfa . They collect milk from our women, but they do not pay for it till the 5^th^ or 10^th^ of the month. I had once even stressed it in a meeting here.

**Well, can the dairy factory reduce your income?**

Of course, it decreases our income, because they pay at a low price.

**Then what?** **Is there another problem too?**

We sell milk at 600 Fcfa a litre here at home. On the contrary, we were told that their implantation here would decrease the price of cotton seed as well as the prices of cattle feed.

**What other problems do you have?**

There is not much food and it is expensive. In addition, we do not have a machine to lift our cows; we do it ourselves.

**Now, what problems do you have with respect to milk?**

The problem is that we no more have money to buy cotton seeds and “rakal” on the market. These foods are the best. We are also affected by water shortage. This problem has been mentioned several times during meetings held here.

I have a question: Let us assume I have 100 cows. I go to the bank to ask for a loan. I am refused the loan and the bank grants a loan to a farmer. I have noticed that a farmer always has guarantees. Yet I have never seen someone ask a breeder to secure his cows to be granted a loan.

**This is the problem with banks**. **Here we are concerned with problems related to milk production.**

There are problems with animal feeding and water shortage. Water is our main trouble.

Idi, takes a slate and write “animal feeding”. They will understand.

So the problems are linked to animal feeding, access to water, access to credit and the dairy factory. They did not keep their promise and their price is too low.

**You** **mentioned all the problems** **.** **Hence what can resolve these problems?**

Only those who come to advise us here and God can help. We think of people like those who have come from England. We also need banks that can fund us. We need partners and the problem will be solved.

**What are the diseases that mostly affect cows?**

There is a disease in the belly of cows : plastic bags . You cannot see them and before you know it, the cow dies. It is too serious.

**Plastic bags are not a disease.**

There is “sontose” and the three-day fever disease. There are two diseases that are the most dangerous: pasteurellosis and cattle dermatitis. There is more serious disease called rabies.

**Now tell us which one is the most dangerous disease.**

There are pasteurellosis and skin dermatitis. The next one is “handé”. I do not know the word in French.

There is also “sontos” who follows after the “handé” . “sontos”  is when the cow loses weight . Then we must call the veterinarian for she knows the names of the diseases in French.

If the cow gives birth and refuses to breastfeed, we really need this treatment. There are thieves too.

**Hello** **veterinarian.** **How is “handé” called** **in** **French?** **Write** **it in Pulaar. I will translate.**

**It refers to respiratory infections.**

**Only a surgery can treat it.**

Now she says we must classify the diseases in pairs in a table. Then they will be able to compare.

We must pair tripanosomiasis  and diarrhea here.

For all cows that suffer from fever, they systematically talk of “sibourou”. What you must do is to say that if you observe this state after three days and if the animal does this or that, thus we know it is the three-day fever sickness.

***Explanations in English* (1h 42 min – 1h 42 min 10 sec)**

**That is, we compare pasteurellosis and cattle dermatitis as well as  pasteurellosis and throat infection. Pasteurellosis is the most serious disease.**

**It will take some time.**

**Manou , Manou , Manou, come after….. ok.**

**Can I delete this?**

**No. To explain that, maybe you should understand the figure alone.**

**They will be alone.**

**If you do not follow and you do not understand, you will have problems if you are alone. Therefore if someone does something, you have to compare it it. André, I am expressing my understanding of it. For example, if you put the column, then you go to the maximum. If you are told that pasteurellosis is the number 1 disease, then you start from the bottom to the top.**

**No, that is not the case. In fact she says that we should compare disease 1 and disease 2, disease 1 and disease 3.**

**It takes a little while.**

**All diseases?**

**You take line by line and you compare.**

**It's not easy, huh.**

**Is that?**

**Yes.**

**You take the various diseases and you compare them.**

**She says she wants the matrix.**

**It allows you to see the disease that comes up most often .**

**Look at the table to see how it goes.**

**Do not compare what is crossed. Good.**

**It is easy to understand, but it takes time.**

**We compare diseases in pairs.**

**Yes, that is it.**

**Do not compare what has been barred.**

**What do we place here in that table, numbers?**

**We will pray first.**

**For me, I understand that it is when we total a number of things. I think it is a table on which the five participants must answer.**

**No, no.**

**It is the group that decides, not individuals. The majority must necessarily be considered.**

**Now among the diseases that you mentioned, what did she say is the most serious between pasteurellosis and** dermatitis**?**

They are all serious.

No, one must be more serious.

 “Handé” is the most serious because it kills.

**Does pasteurellosis** **not kill?**

Both kill.

**You told me that** **pasteurellosis** **is the most serious.**

Nothing is more serious than skin dermatitis. It is the worst.

**Now we will consider** **pasteurellosis** **to be compared with other diseases first. Thereafter we move to the next one.**

Do you ask about the severity of the disease or about how extended it is?

**It is how frequent the disease is.**

The gravity and regularity are two things.

**Does it mean if you are told today that one of these three diseases must forcefully affect your cow, which one will you choose?**

Regardless of the disease that affects my cow, this is not good.

Pasteurellosis can infect the cow and be cured. “Furtoses” can contaminate it and be cured. However when the cow is infected with “handé”, it  dies.

**So is** **“handé”**  **more dangerous than** **pasteurellosis?**

“Handé”  is never cured.

**Now which one is the most dangerous between pasteurellosis and “sontos”** **?**

Pasteurellosis  is the most serious.

**Now what is the most serious between** **pasteurellosis** **and an aggressive cow (Mad cow?)?**

The(Mad cow?) aggressive cow is the most serious.

**What is the most serious between** **pasteurellosis** **and skin dermatitis?**

Pasteurellosis.

**And between pasteurellosis and “thiare”?**

“Thiare” no more exist nowadays.

**So** **pasteurellosis** **is the most severe.**

**The disease that mostly comes back will eventually be the most serious and the most dangerous.**

**What is the most severe between “sontos” and cattle dermatitis?**

It is the dermatitis. “Sontos” can be cured.

**You know** **diseases are not the same.** **There are two types of cattle dermatitis.** **There is something called** **lumpy skin disease (LSD)**  **and another one called**  **dermatophilosis or dermatophytosis. They are not alike.**

**Here, just put** **DMC which is much easier and represents the same thing.**

**Do you vaccinate them following the** **State Programme?**

Of course, they have been vaccinated.

The State Programme focuses on two diseases:  the cattle heart disease and rabies. They are vaccinated against these. In case the cow presents symptoms of other diseases, you have to inform the veterinarian for another vaccine. The government has only given these two types of vaccines for free this year.

What does DMC mean?

It stands for “dermatose modulaire contagieuse” translated in English as Lumpy Skin Disease (LSD).

The DMC (LSD) is more serious than rabies because it is in the belly.

**What is the most severe between throat infection and “sontos”?**

“Sontos” is not as serious as it seems. Throat infection kills quickly.

Even when a cow is contaminated by “handé” , it has no more milk. Unfortunately there is not a vaccine against it yet.

Plastic bags constitute a very serious issue, because when we do not see when the animal eats it. No one can know.

**Do you have many cases of mad cows here?**

Yes, there are.

Listen to me here. Tell her that the first most severe disease is mad cow; thereafter comes “handé”.

“Sontos” is not as serious. This disease is caused by a fly. It heals quickly.

**Rabies has not been** **added to** **the list.**

There is rabies.

No, but on this side.

**Yet rabies is the most dangerous.** **So can we correct the table?**

Rabies is the most dangerous. When compared with other diseases, it is the most serious.

**We will take a picture of it and you will correct that after.** **We will do on another sheet and it will be better.**

Ha, have you not finished the questionnaire?

It is too long.

You did not include plastic bags.

**But plastic bags are not a disease.**

They are a disease.

Gomis, people are getting tired. we have to go back.

Let us finish with the last page; it is the last question.

**This is what is left:** **How do you care for your sick cows?**

From time to time, there are people who look after their cows themselves. They will buy their drugs and do the injections themselves. If the disease is serious, they go to the veterinarian.

**Now where do you take these drugs to cure** **cows?**

We buy these drugs.

**Where do you purchase them?**

In pharmacies. There are pharmacies for cows . If you explain them the disease, they sell you the appropriate product.

**Do you vaccinate your cows?**

Yes.

**Which vaccines?**

Paterlose and tera.

**Are these vaccines good for the cattle?**

Yes, they are good.

Do you want to go?

**Hence, do people drinking milk think of cow diseases when they buy milk?** **Do they not ask you about?**

No, they buy and leave.

We always have customers.

They have no requirements, they trust us.

**We thank you very much.**

**END OF THE TRANSCRIPT**
