# Supplementary material for: “Everything in this world has been given to us from cows”, a qualitative study on farmers’ perceptions of keeping dairy cattle in Senegal and implications for disease control and healthcare delivery
Source: PLoS One. 2021 Feb 25;16(2):e0247644. doi: 10.1371/journal.pone.0247644 (PMC7906343; doi:10.1371/journal.pone.0247644)
Supplement: S1 Data — (ZIP) [file pone.0247644.s001.zip › Data/18501 FN1 English_final.docx]

**18501** **_** **FN1**

Introduction in English (FACTICK focus group with men of 18 years old and above at Diender village)

**Question 1** **: We are going talk about the importance of milk production** **in relation to** **other** **products,** **nowadays and** **in the future**. **How important is** **milk** **production** **to you foremost** **and to your family?**

**Abou:** I can say that milk production is actually important. It generates some income which allows us to pay some of our workers. It also intervenes in feeding since most often in the night when you have milk, you can eat millet and milk. So it goes into the family diet. It stands as another source of income as it provides money to satisfy other needs. This is really how milk production is important, although it is not enough because we do not have milk throughout the year. Nevertheless, when milk is available, it really used for consumption and sales as a source of income.

**Question 2**   **:** **Apart from milk production,** **are** **there also** **other sources of income in the livestock sector?**

Apart from milk production in animal husbandry, people also sell fattening for animals, even if the feeder is not modernized. From time to time, the farmer sells a bull or brings a bull into family ceremonies; this is farming too.

In family ceremonies, it is farming too.

Agriculture is another source of income.

Animal husbandry also helps us to enrich our soil in order to increase our production. In the neighborhood, those who have cattle are the ones to have most of millet during harvest.

**David**   **:** As he said before, milk is only for a period. During the rainy season many people have milk, which is not the case during the dry season; thus you make up here what you loses the other way.   It also depends on the rain, which is what I experienced. There is not much grass. When the rainy season ends, the flock has no more grass and the farmer spends to feed the herd. Thus it can hardly be expected to be profitable. To me, milk production is important because we make profits. Some people get more than 10 litres of milk in the rainy season, but they are forced to buy livestock feed (“ripasse”) at the end of the rainy season if they wish to continue milking.  I have noticed it. Selling milk also enables us to take good care of our animals because we use its profits we take to buy animal feeding. Indeed it brings us food for ourselves and for our cattle.

**Thank you very much once again for your participation.**

**Another participant**   **:** as everyone said, we all live in a family here and the activities are the same. We have the same experience because we all experience whatsoever any of us goes through with his herd. Therefore there is only one person who can speak for everyone. Well, as it is a debate, each must give his opinion but all the ideas are the same. As we said, we face the same difficulties. As my friend said, selling millet and milk helps us to satisfy our needs until the next rainy season. Yet we are tired of the problem of animal feeding. To feed the cattle, you will simply use herbs if you don’t have enough money. Rather in case we have money, we can easily get access to animal feeding and we would be satisfied.

**Question 3**   **:** **Apart from** **the milk and agriculture, do you benefit from some support such as a child living outside the home who would help you in difficult moments?**

**Respondent**: Indeed concerning what you say, they not only provide financial assistance but the one who comes on weekends will go in the fields or to graze the herd, because agriculture and animal husbandry are all we have.

**Do those living abroad financially help you?**

Actually, they do. It reduces our suffering and helps us to increase our livestock and agricultural production. What I would like to add about how flocks are perceived by our society is as follows:  some flocks are made up of three cows while others have 10 cows. It is not my flock alone but for the community. That is why in hard times (except during wintering/rainy season), each brings the little he or she has. If you know that you have of cows in the flock, you will sustain whoever manages the herd because he cares of your cows at the same time as its own. This financial support is very normal.

**Now here is the second question as everything is centered on agriculture and livestock breeding.** **Now if I** **pick ten pebbles, can you tell me between these two activities the percentage of the most common one?**

Hold on first. We also practice commerce here.

**Now except these three activities, are there other activities that are practised here?**

There is fishing. There are fishermen in Ndiaye-Ndiaye.

**Among these activities, I would like to know the most common ones order of importance.**

**What is the most financially profitable activity among these four?**

The most practiced activity is agriculture. Through for example the sale of millet or peanuts, this activity helps us to buy animals. Animal husbandry comes in second position.

**We would like to know how profitable they are.**

Even then, agriculture is very common, followed by livestock breeding. So place eight pebbles for agriculture, four pebbles for animal husbandry, one for trade and one for fishing; place the remaining ones here.

**Do you think there may be changes in the coming years as today is dominated agriculture followed by animal husbandry, fishing and trade?**

I can answer you it is agriculture that we take most to heat because it is agriculture that can develop the country and we do not think to exchange it with anything else. To me, if we do not move the agriculture industry forward, it will not be anywhere else.

If I understand correctly, you want to know if the other sectors may be more developed than agriculture. Certainly; if you consider animal husbandry, almost everyone is involved it. Nowadays some breed cows, other sheep. With the modernization, people are doing crossbreeding in order to increase milk production. Production is limited with our local breed. I have seen tailors and mechanics engage into crossbred races livestock. Yet farming was only meant for the farmers for the two activities go hand in hand. Over the years, I think that the livestock industry may expand compared to agriculture. I have not said that they will be at the same level but its growth will be faster than that of agriculture. Agriculture requires that the soil must also be preserved whereas it has been noticed there are less fertile lands. I do not say we will do extensive farming, rather intensive livestock breeding. For instance, I know this person has no cow, but I'm afraid that in the next few years he will look for a crossbred race. It is the case with so many other people in the village. So extensive livestock production may not develop, but intensive livestock farming has begun. Even us who have the common flocks are engaging into intensive livestock production. So I think that in the upcoming years, animal husbandry is going to expand and if it does, trade will also grow since we will sell more milk.

To me, agriculture will sooner or later be dominated by the animal husbandry, because the village is growing day by day and agricultural fields are less available.

So if I understand correctly, in the next five years the animal husbandry will take over agriculture due to land pressure. The village is expanding; arable land gradually becomes scarce whereas livestock breeding can now take place in a corner of the house.

**What is the size of the largest herd in the neighborhood?** **Since people are not used to reveal the size or the number of cows they possess, let us make a range from 1 to 10, 10 to 20 and so on. Mark a cross (x) in the corresponding column.**

**We will not do as we did yesterday.**

**Now, we will draw a table.** **So,** **you put** **100 there for example and you put 1 here.** **Now the table is there.** **Do you know what we will** **do?** **You write** **1, 25,** **50 and 75 here. That is it.**

**It is not necessarily fixed; we could also have put from 1 to 10. What we do there is just because I know that no one will say** **the number of cows** **he has.**

**So the table is there. It is essential that each of you draws a line** **where he thinks to be, that is, between 1 and** **25,** **between** **1 and 50 or between 1 and 75 or 100.**

**So do I place a cross or well?**

**Yes, you draw a line.**

**So a line, how?**

**You draw a line like that.** **There where you are.**

**Now over there, you are going to indicate where you are, concerning your number of cows.**

**Hi. It is too much between those two.**

**Abou, place the line. You have more.** **Abou, you place it.**

*Section in English* (25 min 17s - 25 min 23s)

**In fact, it is just to progress somehow.** **It is because of the following question; that is why.**

**So we are here.** **Does everyone recognize his line?** **Okay.**

**Now, here is the next question: where do you think to be in the next 5 years?**

**No,** **you need to** **know** **first at which level you were 5 years ago.**

**Who owns this?**

Me.

**Well, you know that here is where you are now. Where were you five years ago?**

You know I told you that someone can have 10, 15 or even more cows in a herd. In the next five years, a herd may include 100 heads. If a shareholder has 70 cows and decides to withdraw his cows, the flock will diminish. Therefore it will be difficult to know whether the herd increases or reduces because the herd does not belong to a single person.

 So that's a question that - I could even be there, as I could even be there, but you must compare it in another option. You know at the beginning I told you that concerning the flocks, this one brings three, the other brings four, the other also brings some, but there is a herd manager. You must tell him that.

**Ok, I'll tell her.**

*Section in English* (26 min 45s)

**Hence, that means that the flock is supposed to have been more than five years later. However** **if** **a shareholder removes his cows, you know that the flock will go down. Then I would like to understand whether the herd increases or decreases in this case.**

**Well, how do you evaluate your herd five years ago compared to what you possess nowadays?**

**I can say that the flock was pretty much this five years ago.**

**That is it.** **That is what we want to know.** **If it turns out that it is a group for example in which you know you had five cows, then your five cows were all of a sudden among ……… (Clumsy statement as it is not finished)**

**Oh, if that is the case, then it increases.**

**Yes, that is what you had five years ago. How many do you have today and to which number do you wish your cows to increase in the future?**

**Then, it can be here.**

**This is five years ago**. **Were you there?**

Yes.

**Did they belong to you, David?**

Yes.

**Where were you five years ago?**

No, I was here.

**Okay. Samba, do you recognize your line?** **Where were you five years ago?**

Well, my flock was more than that at the beginning. However, some people came to take their cows back; they were in my custody. Consequently, my cows have decreased.

**But did you know how many cows you had?**

Yes, I did.

**Now, how is it compared to then?**

It has decreased.

**If it has decreased, then draw your line below.**

**Give it to another person.**

**Idi, the question asks to know** **where you were five years ago, where you are today and where you would like to be in the future.**

**In fact, we did not do it yesterday. Rather we did these other two.**

No, we did it.

**We did all the three then.** **Okay.**

**Where were you five years ago?**

I was here.

**Did they belong to you?**

**Okay.**

**Where do you expect to be in the next five years?** **Where do you think you will reach?**

**Put everything, put everything.**

**It is necessary to put the maximum. One must increase.**

**Yes,** **yes, that is it.**

**After, you do the same again.** **It is well.**

**Erase that.**

**Now, it is milk.**

**Write** **milk.**

**How much milk do you produce per day at the moment?**

**If you say the year, you need to indicate the season there.**

You know, milk production depends on the season of the year too.

**Now,** **we will do** **every** **15 days or every week.** **It is the same procedure.**

**How much milk do you produce every week?**

It is not obvious now. You know it as much.

**Put the three points.**

**For instance, write 2, 4, 6, 8 and 10.**

**You could be at 5, 10, 15 or 20 per week. That is the milk a week.** **It is well.**

**It is zero there.** **So, you produce 8 liters or 9 liters every week** **from Monday to Sunday. Then this is between 5 and 10.**

**Now, to those who have cows and milk, whatever you indicate will be good.**

**Per week.**

I do not have milk.

**That is good.**

**Abou** **Thiam, come here. You will fill in yours.** **You have plenty of it.**

**You must fill in.**

**Let us still fill in for five years ago.**

**For today and in** **the future.**

**Do it. Here is** **the pen.**

**NB**   **(Concerning the questions on** **the number of cattle and the number of liters of milk** **produced in the past, nowadays and possibly in the future, they put figures in the sheets distributed to participants.  Thus, refer to the sheets).** **From 22mn 16s to 43mn 43s**.

What I was producing? So I was there and I was producing more.

**Where were you?** **Were you producing more or you were producing less?**

I was producing less.

**Now, let us look at the high season, that is, after the rain.**

**Wait, let me take a picture of this first.**

**Take that one at the same time.** **Take it, let us change color.**

During the rainy season, what I produce a week can reach here.

Per week, he can have 20 litres.

**That is good.**

**What caused changes?**

**What caused changes in milk production in the past?**

It depends on how oxen and cows mate. The problem is not cow feeding, but the number of cows that are pregnant.  The more cows deliver calves, the more milk they will produce. The type of cow matters because there are productive cows and less productive ones.

Coming back to what Samba just said, the cycle is also to be considered. If the cow gets pregnant too soon, you can expect to have much milk in the rainy season. Calves suckle for 2 months before milking starts. Nursing cows may produce much milk in May, June and July because there is a lot of grass at that time. When there is no more grass, cows that continue to breastfeed in September and October no more have enough milk, including those that put to birth that period.

As said before, the climate causes change in production. In the rainy season, there is a lot of grass which corresponds to good milk production. In the dry season, there is not much grass which corresponds to a shortage of milk production.

**What changes have you implemented to have good milk production and why?**

The race has been improved through insemination. The program concerns dairy breeds. We currently work in a program with the State and the private sector as well as some individuals. In our department this year, approximately 80 to 90 cows have already been inseminated. If this is successful, we will improve the dairy breed. Many farmers like me already have crossbred cows. When these animals will start producing, they will be able to give you 7 litres of milk in the morning and 7 litres in the evening. A local cow produces 2 litres in the morning and 2 litres in the evening. Crossbred cows are not mixed in the same herd with the others. I presently have four of them. We think that the production will increase in the future.

**Question**   **: Will you encourage** **your children** **to practice animal husbandry and agriculture?**

This is obvious. Our children are already involved. Even if they come for the week - weekends or holidays, they are in the field during the rainy season, they are with the herd in the dry season. They practise animal husbandry and agriculture. This is the case until our children have their own work, but it does not prevent them from coming here to do farming and livestock breeding. All the time, agricultural activity has been associated with animal husbandry. Most people currently have their herds in the bush. Few are those who have cattle at home. Be it 20 or 30 animals, they are lead into the fields because agriculture without inputs cannot work. That is why herds are taken into the fields to produce organic fertilizer. The races that are inseminated to increase milk production are the brown Zelter or Maubelane that produce more milk. I think a thousand heifers have been imported. They have been distributed, but we do not know to whom they have been given. They were meant for, us but we did not see them. A breeder who has 50 to 60 cows cannot benefit. We do not know if they were given to the people of Dakar or Sangalkam; we do not understand. Pakistani breeding stock and Buzzera breeding stock from Brazil were also sent. The thousand pregnant heifers were intended for breeders, provided they had written an application. They were six months pregnant. They were sent for breeders to increase their milk production.

**Question**: **What obstacles and difficulties do you face in milk production?**

The main obstacles mainly concern feeding. For example, a cow must eat well in the dry season in order to produce milk. The second problem relates to the availability of dairy cows. Also there is a need to master breed pregnancy in the milk production, because if unfortunately a breed that has to give birth this year is not mated, it will not produce milk. If it does not eat well too, it cannot be mated. Likewise if the cow is pregnant and does not have enough to eat, it can have an abortion. That's why it is necessary to get the training and the means in order to enable cows and bulls that must mate to mate. This is why bulls must be well taken care for, even if it is necessary to tie them elsewhere and feed them alone. Heifers must have a good diet.

Here, we regret that a cow puts to birth in the 2018 rainy season for example and should wait till 2020 rainy season to give birth once more. Whereas there are cows that can give birth every year if they are in good condition. Even a man who does not eat well does not think of a woman. Animal feed is available but it is very expensive. A 50 kilo bag of “Rakal” costs 10,000 Fcfa and the bag lasts only 4 days. The kilo of cotton you see there costs 150 Fcfa. Ten years ago, the kilo would cost 60 Fcfa whereas it costs 150 Fcfa or more today. That is the core of the problem, that's what has hampered the sector. Everyone would like to engage into animal husbandry, both farmers and non-farmers. Even those in Dakar love cattle more than us because they are willing to buy a bull at 700,000 Fcfa and a sheep at 500,000 Fcfa. It is nice to have dairy cows and milk them, but it is very expensive. I just spent 42,000 Fcfa on animal feed which will only last ten days. You realize we are too indebted. In the rainy season, we manage to get 10 liters or 13 liters of milk a day with the little grass available. Moreover as people say, we need imported breeds to have much milk but the problem is it takes enough money to give them attention. When you look at them on picture, they look beautiful and well fed. However they will have difficulties living here in our warm climate at 40 degrees or 42 degrees in the shade. The inseminated dairy cows will begin to suffocate if you leave them in the bush at 10 a.m.; so F2 and F3 cannot live here.

**And if we were to classify, what is the major obstacle?**

The first obstacle is the food given is expensive. Thereafter there are dairy cows. Besides, the state also must subsidize livestock producers.

**What are the diseases that most affect the herds and which prevent them from producing more milk?**

We profane we are not veterinarians we have not been to school so we cannot know but the most common are:  trypanosomiasis, pasteurellosis  and anthrax for cows aged from 2 to 3 years old. There is also foot-and-mouth disease. However some deadly swelling of lymph nodes has been discovered: the three-day sickness (or bovine ephemeral fever). The most dangerous disease is pasteurellosis which decimate the herd. Thereafter comes “sonto”, foot- and- mouth disease, anthrax, the three-day sickness and then ganglia.

**Are there diseases that can be transmitted to humans?**

I have not yet observed it.

**What do you do to** **protect** **cows from diseases?**

They are only vaccinated.

**Now if comparison needs to be, which disease is the most** **dangerous?**

It is the pasteurellosis which is the most dangerous of all these diseases, followed by dermatitis. The foot-and-mouth disease comes next. Ganglia and trypanosomiasis  follow.

**How do you manage the health of herds?**

State vaccination programs provide vaccines against pasteurellosis;  the breeder vaccinates cows every three months or six months. For trypanosomiasis, “cevadine” is bought and the veterinarian gives it to the flock on a regular basis. It depends on the breeder because there are others who do it very rarely.

**Do** **you frequently** **fight against parasites using** ‘**igomettre** ‘ **or is it rare?**

It depends on the means of the breeder and the accessibility of veterinarians to advise them.

**Do you do self-medication**   **?**

It happens that we do it because there are not enough veterinarians for those that are sick. We are often obliged to treat them ourselves.

Nevertheless most people are not intellectual like him. So they prefer to wait until the veterinarian comes, even if t is after three to four days. Sometimes they even pay 1500 Fcfa or 3000 Fcfa for his transportation. From time to time, we are obliged to call the veterinarian on phone to explain the symptoms so that he gives us instructions so that we can treat them.

**Do you have consumers who come to buy milk at home every day?**

Of course, each person has his/her own customers here. If it happens I no more have milk, I can recommend the customer to a friend. There is a dairy factory that buys milk too, but you must sign an agreement with the company prior to this.

**However do consumers have requirements on the quality of the milk?**

We use to filter the milk and put it in bottles before selling it. So we most often do not receive any remarks. Rather, with regard to the inner and outer value of our products, customers come to compliment us that we have good and clean milk.

**Do you heat the milk before selling it?**

No, we do not heat it before selling it.

**Why?**

We do not have time to heat milk and it is not even a tradition for us.

**Do you have people who** **come to** **complain that your milk made him/her sick the previous day?**

No.

**No?** **Can it not make a person sick?**

No, this is not what I said but for sure, no one has ever come to complain that the milk has made him/her sick.

Nevertheless a customer told me once that he had tried to turn the milk into curd but he failed. It may be due to the container he used to transform it because no one else has ever made this remark so far. Sometimes it depends on the season too: it takes much time to transform the milk in the rainy season than in the dry season. Besides, the percentage of water in the milk is not the same in the rainy season and in the dry season.

**Do you think that if a person eats the meat of a sick cow, he/she** **will get sick too?**

Of course, we know that any sick thing must not be consume, be it a cow or something else. Moreover since we do not sell meat, the veterinary service is in charge of controlling. Hence we cannot say much about it.

**Do you** **think** **that a sick animal can transmit the disease to you?**

We know that a person who transmits his illness to a person. We have lived with the flocks but we have never known that a cow can transmit us his illness. It is possible. There are people whose animal can get sick, yet he sells it to the butcher and the butcher makes use of it; we do not do it. Without informing veterinarians, butchers can take sick animals, kill them and sell their meat. This is seen here in Senegal. As a breeder if you have a sick animal, you must not sell it. This happened to me last year and someone came to me to buy it. I refused for I was certain that the animal was about to die.

**How do you know that the milk is good?**

We do not have the means to know. Maybe the dairy factory can know.

**So we thank you for your attention and all the time you spent with us. We are listening to you in case you have any question.**

**END OF THE TRANSCRIPT**
