# Supplementary material for: “Everything in this world has been given to us from cows”, a qualitative study on farmers’ perceptions of keeping dairy cattle in Senegal and implications for disease control and healthcare delivery
Source: PLoS One. 2021 Feb 25;16(2):e0247644. doi: 10.1371/journal.pone.0247644 (PMC7906343; doi:10.1371/journal.pone.0247644)
Supplement: S1 Data — (ZIP) [file pone.0247644.s001.zip › Data/18502 FND2 English final.docx]

**18502 FND2**

**Mrs. Ndour: Okay, Ndiaye.**

**Ndiaye: Okay, all right. So the first thing that we will ask you today concerns the cow.  When we talk** **about** **cow** **breeding,** **of what usefulness is it to you** **and** **your households?**

Go on.

 No, you start.

 Cow breeding is of limitless use for the Serer. Uh! To us Serer, cow breeding complements our life first, because we cultivate and we practice agriculture and you will have no harvest if you want to grow plants on a land that is not fertilized. Thus we need cow dung on our fields to work as inputs as we grow peanuts, millet, corn, cowpeas, sorghum, etc.  After agriculture comes milk. Concerning milk, a household that has a herd of cows never suffers hunger. It is of great importance and usefulness for a household to feed on milk couscous and curdled milk. Then, we Serer do baptize. As you see, we had a wedding ceremony today and we slaughtered a large ox here. We celebrate baptisms, funerals and weddings. For such events, it is beneficial to slaughter an ox in case you possess a herd of cows. Next, having cows can help you meet up immediate needs, because having a cow for the Serer is comparable to having an agricultural bank. Instead of going to Dibocor to borrow money when you have a very urgent need, you go take a cow in the flock. You can get a cow from your herd and sell it to 100,000 FCFA or 200,000 FCFA in order to satisfy this need. Consequently, all this makes me say that a cow is of paramount importance among the Serer. Well, I end here and anyone can add extra information in case there is something to add.

You said it all.

**Ibrahima Ndiaye, do you have anything to add?**

**Ibrahima Ndiaye:**I am also involved in these two activities. However we thought that the importance of cattle breeding in our village would decrease these days, especially with the difficult conditions caused by the climate change that has caused lack of fodder. We thought that the livestock industry will decrease here, but it is the contrary. It has been noted an increase in this activity because it is profitable. He just said it and I would really like to insist too much on it. Apart from that, I think you have not mentioned cow dung.

Yes! All this! Its energy.

**Ibrahima Ndiaye:**Yes, it can increase energy.  You see, I have watched that cow dung can help to achieve gas self-sufficiency. I watched it, but I do not know if you mentioned it or not.

Cow dung is of great importance, but it is only used for soil fertilization here.

No, we use cow dung.

**Ibrahima Ndiaye:** Cow dung is used for energy in general. This also answers the question on the usefulness of cow breeding. Therefore, cow dung is of use. I do not know if the horns… The horns are also used in arts.

**In which field?**

**Ibrahima Ndiaye:** In arts. Artists make use of them for art works. That is also good.

There is also cowhide.

**Ibrahima Ndiaye:** Cowhide is valuable, as well as everything on the animal in general.

That is it!

**Ibrahima Ndiaye:**Cow breeding is also a source of employment.

**Ndiaye, why a source of employment?**

**Ibrahima Ndiaye:** It is a source of employment because nowadays, that man for an example employs 3 people whom he pays on a monthly basis.

**Yes, yes.**

**Ibrahima Ndiaye:** They earn this money to feed their households. That is also the contribution of cattle breeding. It is an example as many others.

**What can you add, Sir?**

Our parents made use of cattle breeding to finance our studies. A lot of people have furthered their education till the university that their parents paid for through this activity. Thus, cow breeding is of great importance.

**Ndiaye: What are your other sources of income apart from the dairy cow or the cow in general? The reason is cow dung comes from both dairy cows and oxen.**

I have not understood the question. I have not heard the question.

Me too, I also have not well understood. Please, do resume the question.

**Ndiaye: Do you have other sources of income which are not related to cow breeding?**

We grow plants.

**Ndiaye: Are you involved in other activities apart from breeding?**

Agriculture! Do not go far.

Ha!

We should mention that all of us receive a retirement pension.

Fisheries.

We all receive retirement pension. We do not do agriculture. Hein, that one, all these people receive their pensions as former civil servants. Besides, the context is favorable to us.

**Ndiaye: Yes.**

To practise agriculture.

**Ndiaye: All right.**

And to practise fishing too.

**Ndiaye: All right.**

**Ndiaye: Apart from animal husbandry, agriculture and fisheries as well as the retirement pension you receive, do you receive support from your children?**

We all do.

**Ndiaye: That is it.**

Yes, we all do.

**Ndiaye: All right.**

We educated our children and took well care of their studies. Nowadays, we thank the Good Lord because many of them enjoy very good economic conditions. They financially support us and this is a great relief to us too. Among them, there are teachers and people who occupy various positions in the society.

Let me use cattle feed to better illustrate what cattle breeding represents for me or for you or for another. For example, I will take wheat hay for me: a bag of wheat hay costs 10,000 FCFA and lasts 4 days; a bag of cotton hay costs 10,000 FCFA and lasts 5 to 6 days maximum, and a bag of soy meal which costs 10 000F lasts for 6 days minimum. After estimation, cattle feeding are almost 40,000 FCFA every 5 days. When looking at these expenditures, we are forced to have other sources of income and activities in order to feed the cattle.

**Ndiaye: Here I would like to look at all these activities you carry out.**

**Among these activities you carry out here, can you list the priority activity and the ones that follow in order of importance? You mentioned here cattle breeding, agriculture, fishing, retirement pension and the support from children who are in other sectors of activity. I would like to note here that these activities should be listed in order of priority, based on the level of income that each provides to the family.**

You need to put children first, support from children.

**Ndiaye: Classify the activities first.**

Agriculture.

Be careful. Here, we only think in terms of finances. Nevertheless we cannot separate animal husbandry and agriculture because they both require physical effort.

**Ndiaye: Does it mean that the physical activity that someone invests in cattle breeding is not the same he devotes in agriculture?**

Both are linked and cannot be separated. Agriculture and …

**Ndiaye: Mrs. Ndour, you need to intervene to distinguish  agriculture from animal husbandry.**

**Mrs. Ndour: I hear you now.**

**Ndiaye: Agriculture and animal husbandry cannot be separated. What can we do?**

Therefore this is agro; this is what is called agropastoralism.

**Ndiaye: Okay, all right. After agriculture and animal husbandry, what comes next among fisheries, retirement pension and support from your children?**

Fisheries.

Children, the children.

The support from our children is more important than the income from other sectors of activity, because their financial assistance is substantial and on a monthly basis while the income from other activities is seasonal. The retirement pension comes next.

**Ndiaye: What about fisheries?**

It does not generate much income.

Fishing is not much practiced here.

**Ndiaye: There is someone who has mentioned it here. All right.**

**Ndiaye: It should be classified if it generates income.**

**Ndiaye: In the upcoming five years, do you think there would be some positive or negative changes compared to your current situation?**

Tell him that change can only come from God. If the Lord gives us much rain or excess water, then things will improve and there will necessarily be some positive changes.

**Mr. Senior Divisional Officer, I would like to insist on something: can there be changes?**

**Prefect:** Only if it rains a lot.

 This does not only depend on the rain. Presently, the population increases and available lands diminish due to the construction of building; we face land pressure. This fact may lead to the disappearance of animal husbandry and agriculture because of land unavailability. President, look at Mbane today. If you go to Mbane, farmers can no longer cultivate because of the shortage of available fields, caused by the construction of the University of Sine Saloum and the power station. You therefore see there is a huge problem.

In addition to this, there is a salt issue in Fatick that disadvantages the extension of arable land. Thus this will negatively impact agriculture and animal husbandry from one year to the other and will cause the disappearance of these two activities. We do not know when, but the risk is present; the disappearance of these two activities is predictable. If it happens, we will only be left with fishing, our retirement pensions and the support from our children as sources of income.

The climate and land use by men will promote negative changes on our lives. Moreover, the income from fishing cannot replace the losses that we will experience if these two activities are no more practised. Fishing can be profitable in communities like Foundiougne, because they are close to the sea and it is their main activity. Contrarily here in Fatick, we only know agriculture and animal husbandry.

You ask us questions. We will also interview you later.

**Ndiaye: No, no, there is no problem. All right, they are here for that. You can ask your questions later.**

**Now, what are your constraints within the dairy industry? No, I mean the obstacles in milk production, because there is no dairy industry here. So I mean “milk production”.**

**Tell us about the obstacles you face in milk production, the difficulties.**

**Before, I would like to ask you whether that situation was the same five years ago.**

**No, we are not yet there.**

Concerning milk production, we also face problems. here. We practice traditional cattle breeding that we inherited from our parents. The reason is we too want the herd to fertilize the soil. Thus, it is extra cattle breeding (out of herd?). That causes milk to lack. Now there are people who have begun cow artificial insemination like the *Norman,*the *Mobiliar,* etc…but milk is not yet abundant. We also have a milking centre and more than 75 cows have been inseminated this season. Maybe after that, milk will gradually abound. The shortage of cattle feeding has led to the scarcity of cow milk.

**Ndiaye : Today, how many cattle does the largest herd of cows in the area approximately have?**

In the village, we can see herds of 100, 70, 80, 50, 25, 20 or 17 cattle.

Note from 100 to 17 cattle.

Be careful. It should be noted here that it is possible for a herd of 17 cattle to belong to several people. This should also be pointed out here.

**Ndiaye: When we took contact with some of you here, we were notified that the Serer does not usually reveal his assets. Even if the cows belong to the head of the household and his children, we consider them as a single herd.**

Here, we number from 17 to 100.

**From 17 to 100.**

**Ndiaye: Do you think that milk production can be profitable in the future?**

Water sufficiency and cattle feeding accounted for the abundance of milk in the past. However these resources are scarce nowadays. The profusion of milk is now linked to insemination. If someone does not practice cow insemination, he will not be able to have milk in the future. It will not be a producer in the future.

**Dione!**

Dione: Yes

**Here is from 17 to 100 cattle. I would like you to tell me at what level you are represented on this line.**

Dione: The Serer is not measured. Huh.

**Just give an estimate!**

Dione: Put 70.

**The next.**

Dione: Babou, do not go below.

Babou: No, I am below Dione. I think I must be here.

Dione: Mr. Faye, you know who supervises vaccination … Mrs. Ndour, do not laugh too…. I am the one who supervises vaccination. If I tell you from 70 to 100, then I know each person’s number. That one is far ahead of me. We live in the same house.

Babou: Anyway, joking aside, I am no longer milking cows.

Dione: No, milk has nothing to do with the number of cows.

**Mrs. Ndour: That is the situation for this year. Now, how do you see your situation in the next 5 years?**

Dione: It will improve.

**Mr. Faye: These are projections over the next five years.**

**You see, they can get to this level in the next five years because I have observed it in Ndiandiaye.**

Dione, what do you say on the number of cows?

Dione: Yes, the number.

No, that is not the case because a herd has a limit. There is a high number of cows that a herd may not contain here. In the future, we will be forced to divide the herds because a significant number of animals can be a handicap for its maintenance.

**Mr. Faye: Yet it does not change; it is still a single herd.**

Dione: Mr. Faye, I say that the projection is normal for me because we used to have many cows here in Ndiandiaye years ago, thanks to the abundance of the grass and the existence of grazing lands. Drought suddenly came and the cattle population disappeared. However all warehouses are now full, thanks cooperatives and BNDE. That is why cattle feed is now available and cattle vaccination campaigns are efficient. These combined factors can boost the activity. Every breeder has his vaccine toolkit and he can directly care for his animals without the assistance of the veterinary. This promotes the reproduction of species.

Babou: As for me, I am afraid that there will no more be animals grouped in flocks in the future. With the current assets in the cattle breeding, that is to say cattle feed and vaccines, everyone strives for individualism, which is to breed animals in small groups and in isolation. People will no longer breed animals together; the person who had 10 cattle will go up to 15 cattle and not a large number of 100 cattle.

**Ndiaye: All right, it is true.**

**Ndiaye: We are going to repeat the same thing but with milk production this time. We were talking about the number of cows at the present and in the next five years. Let us move on to the present situation of milk production and in the upcoming five years.**

**Mrs. Ndour: Let us go ahead.**

**Ndiaye: What is the present milk production?**

Dione: Milk production is not consistent for the moment.

**Mrs. Ndour: Can you indicate the daily margins?**

Dione: I can milk 10 litres in this season.

**Mr. Faye: Per day?**

Dione: No, 10 litres in the morning and 5 litres in the evening, 15 litres maximum daily. I am not talking about inseminated animals. This production is for the herds.

**Mrs. Ndour: What is the smallest quantity?**

There is 0 litre. We often have 0 litre. The maximum is 15.

**Mrs. Ndour: Which quantity of milk do you expect to get in the next five years?**

Dione: You can get much milk if you practise insemination. It will progress in the upcoming five years because I have inseminated five cows. So I think I can obtain 20 litres.

**Ndiaye: Do you encourage your children to engage into cattle breeding?**

We can no longer think like that because children have the freedom to choose their future professions, which will be best for them in the future. Nevertheless if you have hectares to cultivate and herds to feed, you have to choose someone among your sons to take after you. Their mothers also have their words to say. Often they do not want their children to get into our activity because the income earned from cattle breeding and agriculture is not permanent.

**Ndiaye: Now, try to identify the obstacles in cattle breeding.**

These difficulties are:

-Shortage of available land that causes cattle grazing scarcity.

- Shortage of water due to the salinisation of groundwater and brackish water.

-Animal Health. It was noted here that despite some clear improvement, the problem still remains.

- The scarcity of manpower to care for the welfare of animals.

**Ndiaye: That is it. Now, can you classify these obstacles in order of importance?**

- Shortage of available land as well as cattle grazing scarcity.
- Water shortage.
- Unavailability of labor force.
- Animal health, as we are now conscious that animals must be vaccinated. It will be better if we can get help as well.

**Ndiaye: So what can be done about these obstacles? According to you, how can these identified difficulties be overcome?**

The State can elaborate a more effective policy that will address land scarcity and cattle feed.

**Mrs. Ndour: I do not agree with the lack of land. It is true that there is less available land. Yet you cannot tell us that you cultivate your entire land without leaving a portion for the cattle.**

Dione: We no longer have arable land.

**Faye: What was your question?**

**Ndiaye: How to overcome these obstacles?**

The best solution that I see is to practice intensive livestock breeding, immobilize the cattle and bring it food and water on site. That is the unique solution.

**Mrs. Ndour: Do you have a safe place where you can gather nearly 30 cows or more?**

We tell you that we realize that herds are gradually split or subdivided. As we no longer have space for grazing, we can use other strategies as others successfully did. For example in Louga, cattle owners build pens in front of their houses in order to tie the animals and feed them on the spot.

Anti-salt dikes can foster the expansion of grazing lands here in the next five years because here in our area, salt constitutes a real handicap. Desalination may promote the cultivation of rice too.

**Ndiaye: According to you, who may help you to develop cattle breeding?**

Dione: The State and NGOs.

Partners can help us.

**Ndiaye: What are the diseases that affect your flocks?**

Pasteurellosis massively kills our herds. Apart from that, there are:

- Lumpy skin disease (LSD) which kills inseminated cows;

-The foot-and-mouth disease;

- The sublingual inflammation which is manifested by an enlargement under the animal’s tongue.

**Ndiaye: Four types of diseases have been identified. You will rank them in order of severity.**

**Mrs. Ndour: No, do not classify them but compare them according to their severity. Let us see, if pasteurellosis and lumpy skin disease (LSD) are compared, what is the most dangerous?**

Pasteurellosis is more serious.

**Mrs. Ndour: What about lumpy skin disease (LSD) and foot-and-mouth disease?**

Lumpy skin disease (LSD)**i**s more serious.

**Mrs. Ndour: What about foot-and-mouth disease and the sublingual inflammation?**

The sublingual inflammation.

**Mrs. Ndour: What if one compares pasteurellosis and the sublingual inflammation?**

The sublingual inflammation is more dangerous because it quickly kills 4-year-old calves.

**Ndiaye: How do you manage the health of your animals?**

Here, we practice self-medication. It heals the cows ourselves because here there is animal health official. It is a major issue to get a veterinarian. We cannot carry a sick cow to the animal husbandry office. We are forced to practise self-medication.

**Ndiaye: Where do the inputs used to treat animals come from?**

 Dione: By our own efforts. If I show you my kit, there are products such as *Peni scipto, Oxytaxilline, Survidune, Penalline* and *Sevamec*. I have all the inputs. If you wait for the veterinarian, you risk losing your animal.

**Ndiaye: You are all breeders. Concerning vaccination, which vaccine do you use to prevent these diseases?**

Dione: For pasteurellosis, the cow is vaccinated every 6 months and the vaccine costs 2,500 FCFA.

**Mrs. Ndour: Is it *Ivomec* or not?**

Dione: It is the vaccine against pasteurellosis.

**Mrs. Ndour: Yes, it is every 6 months.**

Dione: Every 6 months.

**Mrs. Ndour: Excuse me. Before continuing, I would like to know if you have noticed a disease which is transmitted from man to animal or from animal to man.**

**This fact cannot be appreciated.**

**Mrs. Ndour: That is it, continue.**

**Ndiaye: What criteria do you use to decide to vaccinate and the disease against?**

There is a problem of regularity, especially for the vaccine against pasteurellosis every 6 months.

The State also organizes vaccination campaigns. When this happens, we lead the animals to the vaccination park.

**Ndiaye: Do you think that consumers will consume less milk because of its quality?**

Perhaps it will because the quality of the milk changes depending on the seasons and the environment. The nature of the milk can also change from a cow to another.

**Ndiaye: If you go to communities like Koumpentoum, the nature of the milk that their cows produce is different from the milk produced here.**

**Faye: Has it once happened that a customer comes in, notices the poor quality of your milk and decides not to buy it?**

This can happen because the quality of the milk depends on the cow nutrition.

The quality of the milk also depends on the growth of the calf. The more it grows, the more the milk of its mother improves in quality and in quantity.

**Faye: All right. Are there no other questions?**

**Mrs. Ndour: Hold on, it remains this one. Do you sell the milk if you have milked it?**

Dione: We consume milk and sell it.

**Faye: Do you also ensure that the milk is of good quality before selling it?**

 Only the dairy factory can attest to the high quality of the milk, but we ensure that it is consumable.

**Faye: What can alter the quality of the milk?**

**Faye: You mentioned earlier the quality of the cattle feed.**

It does not exclude that we can consume it. Ousmane directly consumes his milk after milking. We have not of instruments for measuring milk quality. Only the factory that buys this milk has the means to test.

**Faye: Do you think that a person may contract a disease while consuming milk?**

Yes, milk can cause diseases if it is not heated.

**Mrs. Ndour: Do you heat milk?**

No. Even here, if a person offers you milk and you heat it, he will no more give you. It is said it causes the udder of the cow to swell.

**Mrs. Ndour: Will you not be at risk if a cow is sick and you slaughter it for consumption?**

A sick cow is not slaughtered here. We only slaughter old cows or if they are fractured… but not sick ones.

**Faye: Do you think someone can contract a cow disease if he sleeps with animals?**

No.

**Ndiaye: Do you face difficulties in selling cow milk?**

Dione: No because we have a dairy factory here. They always buy, even if you have a quantity of 100 litres.

**Mrs. Ndour: Is the price the same to the factory and to a person in the community?**

Dione: No. The factory buys at 350 FCFA a litre and we sell to individuals at 500 FCFA or 600 FCFA a litre. The price is good, just that there is no milk.

Dione: You too, I would like to ask you a question. What have you kept for us?

**Faye: if there are additional questions or sections that I have skipped, you can intervene to complete. I think that I have asked all the questions.**

**Mrs. Ndour: What have you said, Mr. Dione?**

Dione: What do you presently think for us? Do your projections. Veterinarians are difficult to get in the locality.

**Mrs. Ndour: The scarcity of veterinarians is the responsibility of the State of Senegal. For instance, I am a BACC + 7 years degree holder, but I am not able to progress normally and the public service is a little difficult. That is why we experience a shortage of veterinarians.**

**Ndiaye: I would like to extend my warm thanks to everyone first. Then, those who wish to leave can do so. We apologize because we have come during some valuable time. Yet you have been motivated by your love for this activity. Therefore we pray for you, asking that what has brought us together here should be achieved, in other words, that your projects be successful. There is a disease called brucellosis which affects animals. That is what has motivated these people to come. This disease does not yet exist in Senegal.**

**So we thank everyone.**
