# Supplementary material for: “Everything in this world has been given to us from cows”, a qualitative study on farmers’ perceptions of keeping dairy cattle in Senegal and implications for disease control and healthcare delivery
Source: PLoS One. 2021 Feb 25;16(2):e0247644. doi: 10.1371/journal.pone.0247644 (PMC7906343; doi:10.1371/journal.pone.0247644)
Supplement: S1 Data — (ZIP) [file pone.0247644.s001.zip › Data/18503 FL1 English final.docx]

**18503 File FL1. WAV Man**

# Introduction in English (00 sec - 05 sec)

**We would like to discuss with you on cow and milk production.**

**How important is a cow and milk production in your life?**

**Everyone is free to give his opinion.**

We cannot quantify what a cow and cow milk represent for us. They are of paramount importance. Not only is it a pride and an honor for us, but also it can feed your entire family and even increase your life expectancy. A child born in a family which possesses cows is holier than a child born in a family without cows. Therefore, a cow is of considerable importance.

Watching your herd gives great joy and this increases life expectancy.

As said Uncle Sheikh, it is actually of great importance because having cows gives greater peace and therefore long life to our dads. It helps fight against poverty and hunger, especially as cow dung can be used as fertilizer. Thus as you just heard, a child growing in a family with cows is healthier.

A cow is of great importance for, according to wise men, the cow says that in addition to feeding its calf, it also nourishes others’ children.

**What is the importance of milk production to your household income?**

The importance of milk production is considerable. If milk is produced in much quantity, money from its sales may help to recruit a shepherd and ensure household food. This even applies in case you just get two cows.

In addition, it helps us buy cattle feed and pay our water bills along with many other expenses.

**Do you have other sources of income apart from cows?**

Yes, we practise agriculture.

As so rightly said Ousseynou, nothing is thrown, not even cowhide. Cow dung is used as fertilizer to improve our field yield and have food to eat, to sell and even for donations.

We can say that cows are our main source of income. All other activities complement cow breeding. This activity is essential to us.

For example, my brothers are travelling (abroad?), but they only assist with lunch in this house. It is my responsibility to manage the house’s other charges and provide for feeding and other household expenses.

We can nevertheless meet our daily needs without their contribution.

**Can we say that you receive support?**

Yes, you might say that.

Unfortunately I receive no support.

Let me clarify that my brothers abroad and I are one family and we pool our resources. The cows belong to our father too.

We practise animal husbandry, agriculture and tailoring.

I only practise agriculture and animal husbandry.

**Can you classify these activities in order of importance?**

These activities complement each other. Animal husbandry complements agriculture and vice versa. Agriculture cannot succeed without animal husbandry, as well as animals cannot be well bred without agriculture.

**Where can your tailoring job be classified?**

Tailoring ranks the third position, just behind agriculture and animal husbandry.

**In your opinion, can this order change in the next few years?**

No, this order will no longer change, be it in the next 20 to 30 years. Tailoring will never take the first rank.

**Can animal husbandry and agriculture change?**

No, they cannot. Whatever you earn in agriculture is used to feed livestock as well as animals are used to promote good farming. These two activities therefore complement each other.

**In your opinion, will arable land not decrease in the next 5 to 10 years with the construction of new houses?**

Indeed, arable lands have considerably decreased.

**Can this negatively impact agricultural production?**

Certainly, it is possible. However, it is not very likely to happen because our economic development mostly depends on these two activities.

People will change strategies in case land becomes scarce. People will replace extensive agriculture with intensive farming. Breed races are already getting improved. If the results are successful in the next 10 to 20 years, there will be a limited number of cows to shepherd to grazing lands instead of 200 to 300 cows. A hundred traditional cows will be replaced by 20 improved bred cows producing more milk.

**How many cows does the richest cattle owner have?**

He has 100 cows.

**How many cows does the smallest cattle owner have?**

 1 cow

**Can you indicate to us your position from 1 to 100?**

I am down.

**What about you, Modou?**

I am at 30.

**In your opinion, will the number of your cows increase in the next 5 years?**

It depends. I think that it is tiring to a certain extent to have a high number of cows.

However, care is taken to keep breeding on the rise. At the same time, older animals are sold. Therefore I am for the principle that promotes quality to quantity.

**Sir, what number of cows would you like to have in 5 years?**

I would like to reach the bar of 50 as the other person has much more resources than me.

**Yes Ousseynou, do you have something to say?**

Yes, you know we are Serer. The number is very important for us. Unlike the other person, I am involved in traditional animal husbandry meanwhile others practise modern livestock breeding.

**Please, answer my questions before we proceed. Has the number of your cows increased or not?**

Today there are many difficulties because my idea may be different from that of my children.

I can think that I have to be more active on animal husbandry. However they think that they must rely more on studies and do something else. Consequently, it will be difficult for me to achieve my objectives.

**Perhaps among your children, some will share the same philosophy with you.**

 Yes, it is possible.

I think that even if your children do not buy your ideas, you may still succeed if you work hard with a good organization.

Even if traditional breeding is still practised nowadays, we can say that habits have changed a little because if there is an urgent need, a cow is sold to meet this emergency.

**Among you, how many litres is the smallest production quantity per day?**

My lowest production is 5 litres per day.

**What is your lowest production per day?**

I produced in small quantity. It happens I do not even milk in the evening.

It is normal for him to have a low production because he practises traditional cattle breeding. So my production is 2 litres a day.

**To you involved in modern cattle breeding, what is your biggest production at this time?**

 Our production is around 10 litres a day, morning and evening.

As for me, you can indicate 13 litres per day at most and 10 litres at least.

**In case you have cow births, can you produce 15 litres?**

Once more, it depends. Even if you have births but not from cows that have been inseminated, the production will always be low.

However, you can still produce a lot of milk if you have 2 or 3 improved cows.

**During the rainy season, do you exceed 10 litres per day?**

It depends. It may happen that the calves are weaned during the rainy season. In this case, the production will be low.

**What was your milk production five years ago?**

I exceeded 15 litres, but I did not have as many animals as I have today.

As for me, Diaw, I would harvest 14 litres per day.

**Ousseynou, what was your daily production?**

I could have 10 to 13 litres per day.

**What daily production do you want to achieve in the next 5 years?**

We would like to produce the maximum possible. For example, we would like to produce 100 litres or more with 10 cows.

**In your opinion, what can help you achieve these objectives?**

We would like to have competent veterinarians and benefit from a considerable drop in the price of cattle feed which is presently expensive.

Diaw has said it all. Sometimes we may have an emergency with a cow, but we have no one to attend to you. In such case, we are forced to use our local practices to heal the animal.

If all these conditions are met, we will be more motivated to produce.

For example, one of our parents had a sick cow but he spent two days without finding a veterinarian.

**What else do you face apart from these two problems?**

No, there is nothing else.

**In your opinion, what are the solutions that can resolve these difficulties?**

Good seeds must be put at our disposal, especially for artificial insemination.

We are often offered very worst types of insemination that cause us much worries.

**What are the measures to be taken to increase milk production from 20 litres to 60 litres per day for example?**

Reduce the price of cattle feed and facilitate access to it too. If the cow is not well nourished, it cannot produce enough milk. We need access to the veterinarian as well.

**What is a priority between cattle feed and the veterinarian?**

They go hand in hand, the veterinarian and cattle food. It is useless to have cattle feed without a veterinarian. Likewise it serves no purpose to have a veterinarian without cattle feed.

**What are the most frequent cow diseases presently?**

The most frequent diseases are: pomology, spots, lumpy skin disease (LSD), "chaufé", “safa” (foot-and-mouth disease FMD), the three-day sickness (‘sibirou’ (bovine ephemeral fever?) and calcium (calcium deficiency). Pasteurellosis also has devastating effects here. There is also the PPCV.

**Do these mentioned diseases have impacts on people?**

Yes, there are some of these diseases such as tuberculosis which are transmitted to a human being. This was unknown in the past, but a veterinarian has sensitized us on tuberculosis infection.

**If one compares pasteurellosis and spots disease, which is the most severe?**

Pasteurellosis is more dangerous than spots. Yet, curing spots is very difficult.

**Which is the most dangerous between “safa” and spots?**

Spots are more dangerous than “safa”.

**What about tuberculosis and spots?**

Spots are more dangerous than tuberculosis.

**What about “safa” and pasteurellosis?**

Pasteurellosis is more dangerous than “safa”.

**What about pasteurellosis and calcium deficiency?**

Pasteurellosis is more dangerous than calcium deficiency.

If you inject a cow suffering from pasteurellosis, the animal dies. This happened to me on four occasions.

**What criteria do you rely on to compare?**

In fact, it is very difficult to heal spots. Unlike “safa” which can be healed in 4 to even 5 days, “safa” (spots are) is almost incurable. Calcium deficiency is also very dangerous because a cow does not usually fully recover from this disease.

**How do you manage cow health with regard to milk production?**

As far as I am concerned, I only buy antibiotics.

**What types of antibiotics do you use?**

I use Oxytetracycline 20 percent. I also use a vaccine, Surimidine, which is mixed in water and given to cows from the month of June.

As for me, I call for a veterinarian.

**Where do you buy the drugs that you give to cows?**

They are bought at the veterinary pharmacies and science labs.

**Have you ever purchased them from market vendors?**

Yes, they are sometimes bought at weekly markets.

**Do you consult the veterinarian prior to buying drugs?**

No, we do not have a veterinarian. There are just two private veterinarians here. One lives in France and has just opened an office here.

**What is his name?**

Adam Séne. There is another person named Ousmane Ba with no skills and nobody has need of his services.

**Are vaccinations here carried out through campaigns or individual initiatives?**

I voluntarily take upon me to vaccinate my cows.

I vaccinate cows against pasteurellosis every six months. We also buy products to kill parasites.

**Are there people controlling milk hygiene?**

No. It is true that some shepherds do not control milk quality and hygiene. Everywhere is dirty.

Listen, elders used to tell us not to worry too much about what is being done concerning milk quality. I consume milk. In addition everything I can consume, I can also sell it without problem.

He is a trader. If he was a good shepherd rather, he would act differently.

Milk quality is rather controlled in the dairy factories.

**What can make milk unfit for consumption?**

Milk? Nothing can make it unfit for consumption because milk comes from the cow and the gourd is well clean. Therefore, milk can be consumed without problem.

Everything is a matter of psychology. For example a person who prepares the meals in the kitchen, nobody knows in what conditions he cooks or whether care is taken of hygiene or not. Nevertheless this does not prevent people from consuming this food. It is the same thing.

**Can someone contract disease by eating meat or drinking milk?**

Yes, according to veterinarians. In the past, the meat of sick cows was only eaten. Yet the way people used to live is also different nowadays; we are healthier than our children.

If cooked properly, meat can be consumed without problem.

**Did veterinarians tell you about the diseases that can be caused by eating the meat of a sick cow?**

Yes, they say that if a cow suffers from tuberculosis, this disease can be transmitted by eating its meat.

**Can people contract diseases by permanently being in contact with cows such as shepherds?**

Not at all. I am constantly with the herd every day, but I rarely fall ill.

**What difficulties do you face in processing and marketing?**

We face no major difficulty because even if you are unable to sell all the same day, you can put the leftover in the fridge and resell it the next day.

No, I do not agree. Truly speaking, we face a problem selling it because there are often leftovers.

Yes, trade is as such. Milk often lacks on weekends. It is sold at 600 FCFA at home and at 350 FCFA to these people. It is occasionally even sold at 800 FCFA among us.

I have seen someone who has a farm in the area, but he approximately sells 10 litres of milk in Ndiaye Ndiaye on a daily basis.

He goes beyond this entire area to sell his milk to Ndiaye Ndiaye because people have resources there.

**We sincerely thank you because we have understood what you do.**

**There are others who are involved in health-related issues, especially in the treatment of the disease called brucellosis. They are doing a very important job.**

**It is a disease which symptoms resemble malaria; you experience flu, fever and headache.**

**At the hospital, they can administer you malaria treatment without knowing that it is brucellosis. This disease is contracted by cows which in turn contaminate human beings. I remember a year the disease was recurrent and people were saying that it originated from cow milk.  Some family members were even infected with this disease.**

How is this disease manifested?

**It lumps the joints. In addition, a cow is unable to move if contaminated by the disease.**

**However, heat may help the cow to get up during the day.**

**This happened three years ago, but since then everything is moving on well.**

**This disease also had a traditional cure.**

**Concerning the samples that have been taken, the results will be available by next June because the fridge is not available for the moment. It will be once more accessible in two weeks.**

**Do you have any questions?**

In fact, I would like to know why people have the tendency to reduce their consumption of meat. For example, my uncle loved eating much meat.  Now he has told me that he feels now better since he has only been eating couscous with milk for some time. Many people like him have considerably reduced their consumption of meat.

**Yes. Indeed, people are now paying much attention to their consumption of meat.**

**In addition, meat consumption must be reduced beyond 45 years old.**

**White meat, such as poultry, is better than red meat filled with more cholesterol, especially sheep, goat or cow.**

I have another question: what are you going to bring us back as a reward?

**Concerning the tests that have just been taken, if a cow is tested positive, we will come to cure and follow up the animal for a year.**

**Furthermore, we will prepare a report which will be submitted to the State so that the State includes the treatment of the disease in its program, because it is a disease that has devastating effects. In particular, it causes the cow to abort and reduces the capability of the spawners as well.**

**That is all we can do because we lack funding. We are carrying out this research which results shall be submitted to State authorities.**

Is it on behalf of the Ministry of Livestock or else?

**It is on behalf of the veterinary school. I am preparing my thesis and I am passionate about fieldwork because it helps you to practice the profession and provides you a better understanding of on-the-ground realities. It often happens that there is also a gap between theory and practice.**

From time to time, some veterinarians come here and implement treatments that are not successful. In such cases, we usually return to our traditions which sometimes work.

**Yes, it happens at times.**

Actually, I also experienced this same case. My cow was sick, but the treatment that the veterinarian proposed did not convince me. I tried mine and the cow recovered after three minutes.

**You are right. I had once been admitted to the emergency ward and a young doctor who had not yet defended his PhD administered me Valium. As a result, I had been unable to speak for a week and I have suffered from the consequences.**

**END OF THE TRANSCRIPT**
