# Supplementary material for: “Everything in this world has been given to us from cows”, a qualitative study on farmers’ perceptions of keeping dairy cattle in Senegal and implications for disease control and healthcare delivery
Source: PLoS One. 2021 Feb 25;16(2):e0247644. doi: 10.1371/journal.pone.0247644 (PMC7906343; doi:10.1371/journal.pone.0247644)
Supplement: S1 Data — (ZIP) [file pone.0247644.s001.zip › Data/23501 FNG1 English final.docx]

23501 FNG1

**These people with whom I am are veterinarians who care for animals.** **We will take note of everything you say here and we will take a picture after the discussion.**

**We will** **discuss with you on cow breeding practices   as well as the advantages** **and the** **constraints** **of animal husbandry** **in your** **locality.** **We will** **also** **talk** **about the diseases** **that can be observed in the livestock sector.** **This** **conversation** **will be** **recorded** **here** **and** **that person** **will take pictures of the activity** **using a camera.**

**Let us begin by asking you this: what are the benefits of fresh cow milk** **or curdled cow milk in your life?**

**Everyone can speak and give the floor to the next person to add anything.**

The consumption of cow milk is of paramount importance. It helps people to stay in good. Years ago, our forefathers who fed mainly of milk had much greater life expectancy than us. They were strong and healthy, contrary to us who have become fragile because of the low quality of our foods.

Milk is of paramount importance because it feeds the household.  Again, when it is in abundance, we use some quantity for sale in order to meet some household needs. Milk is often not enough because of the shortage of cattle feed.

**Does what you harvest as milk** **represent** **an important source of income** **for the household?**

It constitutes part of our resources, but this is not our main source of income. Fresh milk and milk are essential in our lives, but we have other activities that make our lives easier.

Milk is also important for feeding the new-born baby. If you have a wife who has just given birth and who is breastfeeding but has no milk in her breasts,  then fresh milk,  curdled milk and butter which is at the surface are important to the growth of the baby. If you pour fresh milk or curdled milk over couscous or porridge and you give it to children, they will finish it even though they did not want to eat. During hard times, when there is no money for daily expenses and if you have milk, the only expense you will spend on meals is to purchase sugar. That is why it is often said that out of two people who each have a barn of millet, the one who always has milk at his disposal will run out of millet first. This also shows the usefulness of milk for the welfare and good nutrition of the household.

**Beyond the activity related to milk production, do you have other activities?**

Yes, beyond this activity, we practice agriculture which is extremely important for us as well as breeding.

**Do you practise** **trade?**

Here, we do not really trade. Presently I have children who have just begun receiving payments for a short time; yet since our ancestors, no employee has ever resided in the family.

**Do you currently have people who support you in the expenditures of the household?**

Yes, I have sons who help me with daily expenses, even if it is not much, but not with cattle feed. Each of us has a family member who assists him in the household expenses.

**You have mentioned livestock breeding and agriculture** **here. Which activity is dominant?**

Agriculture dominates; thereafter comes livestock breeding.

**Now, here is agriculture and there is livestock breeding.  Using these stones, each of you** **will represent your situations concerning these two activities**.

**You place the majority of pebbles in the main activity,** **followed by the secondary activity and so on.**

I think agriculture is my first activity.

**Therefore you must place more stones there.** **So agriculture** **is the** **first;** **does livestock breeding come next?**

Yes.

The way he did it is valid for us. Agriculture is our main activity, followed by livestock breeding. We need to put more pebbles into agriculture.

**You come here too.**

I know that everyone here practises agriculture; it is more important to us. The same answer will be found for everyone.

**Is it the same in the rainy season or the dry season?**  **Does agriculture dominate?** **Does the situation described in relation to** **the importance of any of these two** **activities change** **from one season to another?**

If the season is not good, animal husbandry can dominate. Otherwise, it is still agriculture, it is permanent. Most of the time, agriculture dominates livestock breeding.

**Do you think this situation can change** **in** **the future?**

We think the situation will be the same because it has been the same for years. In any case, livestock farming goes hand in hand with agriculture because of the permanent need for soil fertilization, which can promote good crop yields.

**Concerning milk production, do you think that the situation can** **positively or negatively change in the future?**

It depends on a sustainable practice of livestock breeding because if the cattle expand over the years, milk will follow.

**How many cows does the largest herd in the** **locality count?**

Right here at Ngayokh, Sir, the largest herd must count 100 heads.

**Now we are going to take a scale of 1 to 100.**

**As for you, at** **which level** **are you located** **on a scale of 1 to** **100?**

**If you have 1, draw a line here. If you get 10, draw a line here. If you have 20, draw a line here and so on.**

**Where are you positioned?**

**Is it here you are?**

**Do you not have the same herd?**

No.

**So** **where are** **you?**

**You, come and draw a line.**

**Anyone can now tell where their herd has been for the last five years**.

**You, has your herd decreased or increased during the past years?**

No, it has not increased, but decreased.

**If it has decreased, you must draw a line down and if it has increased, trace it up.**

As for me, my flock has diminished.

As for me, my flock has grown. I must trace here.

Me too.

**Now, would you like your herd to increase or decrease for the next five years?**

We all want our herds to increase even before these five years.

**We ask this in relation to the way you work so that the herd increases.**

**Draw a line where you would like it to reach.**

It can even exceed here.

**Each of you come and draw a line where he would like his flock to reach.**

**Now we would like to know the amount of litres of milk you can** **harvest in the morning and in the evening when there is really milk?**

Indeed, it is pumps.

**No,** **if you take everything,** **will you have 20 to 30 litres** **for example?**

Yes, it will even exceed. At this moment, you can have at least two litres if you have two cows.

**And what if it is the rainy season?**

If it is the rainy season, you can have up to 12 litres and only after the cow has weaned the calf.

**Now if you add other cows to these two cows, thus can you have up to 30 liters?**

It will exceed, but you can put 30.

**Wait,** **I put 40 litres. It is better.**

**And you, where do you find yourself? Trace it.**

**Has your production increased or decreased during the last five years?**

**If it has increased, you draw a line up and trace it down if it has decreased.**

It has increased.

**So draw a line up.**

**Come and also draw a line.** **Everyone must draw**.

**Where would you like the production to reach in the next five years?**

**Come and draw in turn.**

**So all of you would like it to increase?**

Yes.

**Why this change?** **What is at the origin?**

These changes are due to the practice of cow insemination and the improvement of animal health thanks to the intervention of veterinarians and animal vaccination campaigns in the locality. There are a lot of diseases that have disappeared thanks to the efficiency of drugs and also the availability of cattle feed.

**Do you think that** **milk production will improve?**

Indeed, we are certain that there will be change because we now benefit from the support of the State with regard to the access to inseminated cows which produce much more milk.

**Will you encourage your children** **to** **practise livestock breeding and agriculture?**

Initially, we motivate our children to strive in education. Only if they cannot succeed in this area, then we encourage them to get involved into livestock breeding and agriculture.

Now if you have only one child and there is nobody to physically help you with animals and fields, you are obliged to get him involved in these two activities.

Someone who does not have a herd may not encourage his child to practise this activity. He can rather encourage him to get involved in other professions or areas that may lead him to become a breeder in the future.

**What** **constraints do you face in producing milk in large quantity?**

What prevents us from producing milk in large quantity is that we do not breed many animals here due to lack of space. For example, you can have 12 nursing cows; you can only keep three cows in the village and send the others in nomadism.

Lack of staff.

Lack of money for the purchase of cattle feed which is expensive.

The consumption of dry grass because this herb does not promote milk production; wet grass allows the cow to have milk.

**Can you list all these problems in order of impact?**

- Lack of money.

- Expensive cattle feed.

- Lack of wet grass (fodder).

- Lack of staff.

**What solutions do you recommend for** **solving this problem?**

Personally, we think that many problems may be solved if we benefit from the assistance of NGOs.

**We will address** **the diseases that affect the cattle.**

**What diseases are most prevalent** **among** **your cows?**

There is a disease that affects the cow during the rainy season and which is called “three-day fever”, “longhé'', a disease that paralyzes the cow , pasteurellosis*,* lumpy skin disease (LSD) and foot-and-mouth disease (“safa”), which the animal manifests by a runny nose.

**Among these diseases, what is the most common one?**

The most serious disease is LSD. Thereafter follows the three-day disease, then “safa”, ''longhé'' and finally pasteurellosis.

**What is the most serious between the LSD** **and** **the** **three-day disease?**

The lumpy skin disease is more dangerous.

**Now, what between the** **LSD** **and ''longhé''?**

The lumpy skin disease is more dangerous.

**What between the** **LSD** **disease** **and** **pasteurellosis?**

The lumpy skin disease is more serious.

We believe that pasteurellosis is much more dangerous than the lumpy skin disease.

**Now, what is more serious between the three-day disease and ''longhé''?**

The three-day disease is as dangerous as ''longhé''.

**What between the three-day disease and the foot-and-mouth disease** **or “safa”?**

The foot-and-mouth disease (FMD) or “safa” is more serious.

**What between the foot-and-mouth disease and** **pasteurellosis?**

Pasteurellosis is much more dangerous.

**Now** **among these diseases, what do you think** **a human being can contract?**

Among diseases, we believe that a human being can contract ''longhé''.

**How do you know that** **this disease is more dangerous than** **that other?**

We can understand the difference in the severity of the disease because a disease that attacks the cow and cannot be cured if not by prevention is much more dangerous than a disease that can easily be treated by the veterinarian.

**What are you doing to care for your animals?**

If an animal is sick and can walk, it is directly brought to the veterinarian. It is like a father whose son is sick. If now the animal cannot walk, you call the veterinarian who comes to treat him and you pay him. Otherwise we use the traditional method, that is, with tree leaves or by marking the animal using a hot iron.

**Do you practise** **self-medication?**

Yes, we practise self-medication by buying drugs like ''hivomec'' at 500 FCFA and other medicines. However, this is a risk because these drugs are often ineffective.

**Where are** **the medicines you use to cure animals from?**

They are from the market and pharmacies. These drugs come from Mauritania. They are cheaper, but they can damage animal health. There are breeders who only use these products to avoid spending a lot of money at the veterinarian.

**What do you do if your animal is seriously sick?**

If that happens, we try first to understand the disease in order to be able to treat him with traditional medicine, with self medication or by bringing the animal to the veterinarian.

**Do you vaccinate** **cows?**

Yes, we vaccinate our animals.

**Against which diseases?**

Against the lumpy skin disease and the three-day disease. We do not know the vaccine.

**Do these vaccines have an advantage?**

Yes, of course.

**How often do you vaccinate your cows?**

If talking of us, we vaccinate the herd before or toward the rainy season.

**Who makes decisions related** **to** **the practice** **of** **livestock breeding in the household?**

The owner of the house is the decision maker when the animals are in the area. However if they are moved to the bush, the shepherd makes decisions.

**Do consumers of your milk have** **enough** **confidence in the milk quality?**

They come to buy and leave without ever causing us problems.

**Do you have a customer** **who is** **so** **loyal** **that he** **only** **buys** **your milk?**

No, it is difficult to have a loyal customer because it can happen that the demand exceeds the supply.

**May the quality of fresh milk or sour milk cause you not to consume or** **sell it?**

After the milk is milked and put in a container, flies often come in or animals like the dog drink it. If you see it, you will no longer consume it because it is not good anymore.

**After milking it, can you just look at the milk and notice that it is no longer good for consumption?**

Of course, milk is not good for consumption if the cow suffers from mastitis. Blood may come out when milking the cow.

After milking the cow, it may even happen that the milk smells badly before the evening. You will know that it is no longer good for consumption.

Nevertheless, the calf can drink it. Nothing will happen to the animal, but not to man. This milk is usually given to dogs that keep the flock instead of throwing it away. However if there is much, you have to throw it to avoid that children consume it because it is bad for health.

Except blood, the colour is yellowish.

**If they ask you how** **we can help you to improve your herds, you must answer them.**

**END OF TRANSCRIPTION**
