# Supplementary material for: “Everything in this world has been given to us from cows”, a qualitative study on farmers’ perceptions of keeping dairy cattle in Senegal and implications for disease control and healthcare delivery
Source: PLoS One. 2021 Feb 25;16(2):e0247644. doi: 10.1371/journal.pone.0247644 (PMC7906343; doi:10.1371/journal.pone.0247644)
Supplement: S1 Data — (ZIP) [file pone.0247644.s001.zip › Data/23501.2 fng1 English final.docx]

**23501.2 FNG1**

**I would like to ask you this:** **after milking cow milk, do you sell it, offer it or give it to your women for household food?**

Once brought home, we give the milk to women for household food.

It can also happen that we offer it as a gift to a loved one.

**Do you have instruments for measuring the quality of cow milk?**

After milking the milk and if we notice a change of color, we will know that the milk is not of good quality because its color has become yellowish.

Concerning the question of the quality of the milk, one can certainly recognize it.  If you automatically pour the milk of a cow suffering from mastitis in couscous, you can get the bad quality by tasting it. The level of fermentation can also be recognized because bad fermented milk almost becomes like water; it never ferments. Moreover, this milk cannot be sold because of the clear liquid.

**How do you get to know milk of good quality?**

Milk of good quality can be recognized by its white color. It will not be difficult to get fermented.

**Can milk consumption** **make us sick**   **?**

Yes of course, because milk can worsen malaria and fever. For example, it has been more than 20 years since I no longer consume milk. I no longer dare to drink milk because it causes me digestive troubles and makes me pale. It even sometimes causes me stomach ache too.

If you consume milk being infected with malaria, it can quickly worsen your case.

**Does consuming meat of a sick animal lead to a disease?**

**Or can someone contract a disease by being permanently in contact with animals?**

A child who is always with the flock of sheep may get a cold due to the dust they release.

**Can someone contract an illness by consuming animal meat?**

We have heard that if an animal contract a disease and you eat its meat, you may get contaminated. Nevertheless, we have never witnessed such case.

**Is there a disease a person may contract and it is said that it is because** **the person** **is a breeder that he/she** **contracted** **this disease?**

No, this disease does not exist. Otherwise it is not known, because we do not know a disease that an animal can transmit to humans.

**What obstacles do you face in selling fresh milk and curdled milk?**

Here really this problem does not arise because we have no milk to sell.

**Now we have come at the end** **of the discussion.** **We** **thank** **you** **for** **your patience**. **We** **also acknowledge that you have a thorough** **knowledge** **in the field of animal husbandry.** **Samples received here will be studied by the people here present.** **If animals are found carrying** **diseases,** **they will be followed up for** **12** **months.**

**Are the results of the samples that you have just performed on milk ready?**

**No, the agents have not yet left.**

**You will be informed of everything we do. If they find diseases on animals too, they will follow them up for 12 months.**

**Do you have any other questions?**

Now if our cows fall sick and we do not have your contacts, what should we do?

**They have taken a** **sample** **for laboratory analysis. They will come back to cure anything they will find as disease.**

Can vaccination campaigns they organize for humans be also organized in animal husbandry?

**As** **far as** **we are** **concerned**, **we are carrying out our first research on diseases that may be related to cattle breeding**.

**END OF TRANSCRIPTION**
