# Supplementary material for: “Everything in this world has been given to us from cows”, a qualitative study on farmers’ perceptions of keeping dairy cattle in Senegal and implications for disease control and healthcare delivery
Source: PLoS One. 2021 Feb 25;16(2):e0247644. doi: 10.1371/journal.pone.0247644 (PMC7906343; doi:10.1371/journal.pone.0247644)
Supplement: S1 Data — (ZIP) [file pone.0247644.s001.zip › Data/23502 FNG3 English final.docx]

**23502** **File** **FNG3.wav MAN**

**We will now start the** **discussion.** **This work session** **is to know the importance that** **breeding** **has for** **you as** **well as** **the milk production you make.**

**How** **important is cow and milk production in your life and that** **of your family?**

In agriculture, cow dung serves as manure for us. If we grow peanuts or millet, we harvest a lot. Among other things also, a cow is important for ceremonies. For example, when we have a ceremony like wedding, baptisms, funerals, you can kill a cow for it. A cow may also be sold for other purposes or family expenses. So the importance of the dairy cow, really, is immeasurable. Even horns are useful. So a cow is useful without limits. Even the cowhide, horns, indeed all is useful in the cow. Cowhide can be used by shoemakers, craftsmen, and even there are others who use it for other purposes.

The horns are also significant, given that even we the Serer use them for communication. If there are a few very important things happening in the village, a horn is blown in the surroundings villages as an alert. We speak of events such as the death of an old man or an important event that has just taken place. Suddenly, the villages of which I speak to you as well as the neighbourhoods which hear it may come to attend the event. Moreover, the horns are important to traditional healers and others. Even our ancestors used to put things therein to protect the family against external and internal dangers. We the Serer and even other ethnic groups breed animals to feed and sell too.

With the sale of a cow, we can purchase inputs for agriculture as well as we can buy supplies and food. So among other things, the family can feed on just collecting milk, fresh milk or curdled milk. The reason is it can replace rice. In the past, people used to eat curdled milk or “lakh” or “thikhane”. Nowadays, one can do other expenditures with the sale of a cow too. Serer women and especially Fulani much count on that money coming from animals. It is from these that they buy their clothing for celebrations and other events. Household food also originates from there, as well as health care and many other things such as their pocket money especially as any balance from milk money belongs to them.

**Now ask them** **whether apart from cows, if they have other activities or means to get money** **or else if they have children who also contribute to the** **household expenses.**

**Do you have other** **sources** **of income?**

We have sheep, goats and poultry.

**Yes, but apart from** **breeding,** **what** **provides you income the most?**

For example, he is a breeder, a tailor, a farmer and a trader at the same time.

**In these four** **cases** **he** **just** **mentioned, which one provides you income the most?**

Tailoring and other small trades are the ones, because cows do not milk throughout the season and customers might be scarce at times. Nevertheless concerning tailoring, there are customers where we are throughout the year.

**Let us take the five** **most profitable activities for** **everyone such as animal husbandry,** **agriculture**, **trade such as** **going to** **weekly markets to buy a sheep** **or a cow and resell it** **the same day** **or a few days or** **weeks or months later.** **To be able to do** **that, agriculture** **will have to** **be** **profitable** **first**.

**We will classify the four sources of income that you have mentioned. For example, you are eight. If everyone says that it is agriculture, then it is positioned first and so on.**

Agriculture comes first because everyone here practises breeding, followed by trade and sometimes masonry.

Our children also help us because most of them are in the city, where they are working to assist the family. Besides they do several jobs in the city, they do underestimate nothing. What matters is that it should be an honest job. Even young girls are servants in several neighborhoods of Dakar, only to help us.

**Do you have a parent or someone else** **to often help** **you?**

Yes, as we have just said now.

However, some are civil servants as teachers but not everyone benefit from their help, except in exceptional cases only. Our children are in the city to work and whatever work they do, they help us.

**We will now talk about your income** **in** **different seasons** **of these** **activities?**

First of all, we are going to add market gardening in agriculture because it is a culture that is most often practised in the rainy season. It is practised in this area as well.

**During the rainy season, what is the most profitable of your activities?**

Agriculture is most profitable to the family, followed by breeding, trade, market gardening and masonry.

Let us now look at the production. You know that what you have produced in the dry season is not the same as in the rainy season, be it concerning milk or agriculture.

**I think we should ask** **in terms of** **seasons.** **Now out of the five we have just** **mentioned, what is the most profitable activity for the family during the rainy season? Is it the same in the dry season?**

**What if it is the rainy season?**

It is agriculture. And what? Breeding and commerce because although we grow plants, we keep on going to the market to and fro.

**All right**, **okay**. **What else if it is the rainy season?**

It is masonry.

**So** **do you build in the rainy season?**

Of course, there is no time to build. Construction is done even in the rainy season, unless it is raining and cement is likely to spoil.

So here 5, here 4, here 3, here 2 and here 1.

**All right.** **So** **it is agriculture, followed by the animal husbandry, masonry** **and finally market gardening.**

**For this part too, we will** **do classification** **as we just** **did.**

And if it is the dry season: trade, breeding, market gardening, masonry and agriculture.

We put here 5, here 4, here 3, here 2 and here 1.

**So if it is the dry season,** **the first activity** **is trade,** **followed by** **animal husbandry,** **market gardening,** **masonry and finally** **agriculture.**

**This is what those from Leon said.**

**Now, the other question is:**

**What are the difficulties** **you face in breeding and** **milking?**

**What are the obstacles and difficulties you encounter?**

That too, as we can say, you know, you cannot tell that person will have next year what he got last year.

You know that we cannot do that.

To know both ends, that is the smallest and the largest sizes in NGAYOKH, then the minimum is 5 and the maximum is 70. Thus, the size range is between 5 and 75.

**Today, the one with the smallest size has 5** **for example; the** **one with the largest size** **has** **75**.

**How many cattle did you possess five** **years ago?**

That one also, the only thing we can tell you is that as you cannot say that this person got last year, you cannot tell what he will have next year.

**DIOUF**, **as you have done is very good. Therefore that is how you will do for the past five years and the next five years.**

**You know, right now** **where we are.** **However you over there, you will do it for the** **next five years**.

You who are here, if you have 5 cows today, you would like to go beyond that in the next five years.

**Yes, we will come back to that, but for now** **you have been asked if this situation was the same five years ago or if this situation did not exist.**

No.

**Now we** **would** **like you to** **think** **of it as a similar situation, El Hajji.**

**At which level was the breeding** **of the one who has the smallest size?** **How many did he have?**

**And how many did the one with the largest size have?**

**Is this situation you have now similar to what you experienced** **last year?**

No, because you are talking of five years ago. Some people did not even have an ox. Nevertheless they own oxen today.

**This is the reason why I am asking you.** **In those days, how many oxen did the person who had the smallest size have?**

Five years ago, such person had just one and the one with the largest size had 40 oxen.

All right, now you know, in the situation that we are currently facing, the smallest size is 5 and the largest size is 75 at most.

And five years back, the one who had the smallest size had a single animal and the one who had the largest had 40 heads of cattle.

**Now in the 5** **years to** **come**, **how many would you like to** **have?** **For example, how many would like the one who has the smallest size today?**

Yes.

**In the next 5 years,** **how do you** **imagine** **cattle breeding may grow?**

We think he can have 10 heads of cattle.

**Have you said 10** **heads** **of cattle** **in 5 years?**

OK! He can normally have 40 or 50 head of cattle.

If 5 years back he had a single cow and has increased to five cows in 5 years, then he may reach 10 or even 15 heads of cattle, or even 25 oxen, in the next 5 years.

**Yes**, **yes**, **put 20** **or 25** **oxen.**

Concerning that, we can say the one who has 5 today can end up with 25 oxen and the one who had 70 will have 100 oxen.

I have something to add.

**Yes,** **go on.**

It is not because that one has spoken. He is my elder, so I have nothing to say.

**No, it is not like that here; we need all ideas. The way** **it happens here** **is different for** **the Wolofs because there,** **if someone says something that is not correct, other may rectify him.**

Yes, it is clear up this ambiguity that I said that the reason is that you, El HADJI and DIOUF, would like to have 150 to 200 heads of cattle today.

**Ndiaye, you may have noticed** **that I cut you off. The reason is you were answering a question I had not yet asked.** **I hope you understand why I interrupted you.**

Do not think that if Diouf has spoken, then me his younger brother, I must not speak.

**All right!** **I understand what he** **said because** **in this world, people do not have the same education.**

**We** **conduct a study**. **You may tell us things** **that are not right**, **thinking that we** **do not need them...** **whereas we do.**

**Now another thing:** **what is said** **here** **is not forcefully exact but approximate to help us conduct the study.** **You know those who own** **1,000 head of cattle and** **those who do not have among you;** **yet that is not** **important.** **We would like you to tell us things as they are and in a reasonable way. In case you have questions, you can ask them later**. **For the moment, we are the ones to ask questions.**

**It is the same** **situation** **for milk.** **How many litres would daily have the person who milks** **the smallest quantity and the one** **who milks the most nowadays?** **Yes per day**, **indicate the approximate estimation because you do not measure it.**

In this period, we can have a liter of milk per cow everyday, which means that people with more cows will have 4 to 5 litres per day. All right, I know nothing in fact.

**What do others think** **of** **that?**

Indeed, as he said, you can have a litre of milk per cow every day. However, the more the dry season arrives, the more milk becomes scarce. It means that we can only get half a litre of milk a day per cow during the dry season. It also means that no one can even get 20 litres at a time, no. Milk is very rare in this time. The minimum is a litre and the maximum is 5 litres. We must presently meet the herd where they are in the bush. You cannot even have a litre.

You see, you cannot even get half a litre, except cows are here at home and can enjoy other food as cattle feed. You know, you are told that milking is done morning and night. Perhaps there are 5 cows or less than that, that is what we say. That is what I tell you, one to five litres.

**Now, five years ago.**

Five years ago will not be easy. We do not count years, but seasons.

**Now, what if we take the** **rainy season?**

During the rainy season, the minimum is 4 and the maximum is 10 litres.

**Let me summarize:**  **the** **minimum** **is 1 and the maximum is 5 in the dry season; the** **minimum is** **5 and the maximum is 10** **litres** **per day in the rainy season.**

**What might be the cause?**

Five years ago, there was a change in milk production and cow numbers. After milking, some have much milk and others not. Some own 5 to 6 cows while others have 10 to 15 or even 40 cows.

**What is the cause?**

In my opinion, there is an increase in breeding and milking because much more importance has been given to these. If I consider my case, I previously milked one litre, but I now have 5 litres. Breeding has become my strength today because of the importance and confidence I have given to it. If you consider the answers you have been given, the reason is the cattle do not have enough food to procreate and produce milk.

The cows which produce the little milk I have might produce much more milk if I had cattle feed till satisfaction. Thus I would be able to have in the dry season what I usually produce in the rainy season.

**Who** **else?**

If they share the same ideas, it will be much faster.

**All right!**

**You know that you said this is because of** **the diet.**

**Now what have you done to solve this, so that you can have milk in large quantity in the dry season as in the rainy season?**

There are some who store enough to feed their cows in hard times. There are others who say it is a land issue, because people continue to build as you see. As a result, their farmlands decrease as well as space for breeding.

We wish we have people who can help us to have more cattle feed because we lack means. We would grow if we could benefit from “Ripasse” and other feed in sufficient quantity so as to complement what we have. Ii is still the problem of feeding.

**Do you think** **that selling milk can earn you money?**

Yes, it can earn us money. Nevertheless what we produce, we prefer to bring it home for our children before selling it.

Or do you see someone like me? God has done that I cannot have milk; I can only be offered to consume with my family.

Yet for us to sell some, we need to have enough to eat and only cattle feed can make it possible. That is why we always face the same problem of cattle feed. You already asked this and that too.

**Ok**, **it is well.**

**In fact, the problem is what I earlier asked you:** **do you think milk will increase in the next years?**

However, the problem is in the diet.

**Will** **you** **advise your children to turn to agriculture and animal husbandry nowadays?**

No, rather turn to school.

Well, it is their choice.

**All right.**

**What are the difficulties and obstacles related to milking?**

The difficulties always relate to food: first the problem of grazing lands considering that inhabitants increase from year to year, secondly cattle feed, thirdly diseases, fourthly the quality of health care and fifthly the cost of health care.

He talks about the lumpy skin disease; he says that ….

**That is it. We are going to talk about diseases.**

**We will come** **to that**. **It is just** **to tell you that** **according to these explanations, they are things very difficult to cure.**

**Anyway, it is a disease that got them much** **tired.**

Do you know why? It is because we talked twice about care. We talked about the quality of health care and of their high cost.

**Now we will talk about diseases**, **mentioning them one after the other in** **order of importance.**

**OK!** **So, let us presently consider the five**.

**Which** **disease** **is causing you problems the most?**

**No, it is necessary we do it in order because what is said** **applies to** **all** **breeders.**

**Also, if** **their worries** **are not the same** **or are not** **classified** **in the same order, everything** **will be scattered.** **I think it is better.**

Grazing lands, livestock feed, quality of health care, cost of health care and diseases at last.

**Wait,** **we will review the case.** **I** **agree with you,** **you** **brought** **your** **documents.** **However I would like to say that I** **agree with you on the shortage of grazing and the cattle problem.** **Nevertheless I disagree when you place diseases in the last position.**

The reason is before talking about illnesses, it will first be necessary to talk about drugs and their costs...

**It takes** **diseases to talk about care.**

That is what should have come first.

**Yes.**

**Costs and quality can follow after.**

**You** **can** **even do that**, qu**ality and care.**

So that we can know that the drug we are given is not good but expensive.

**So to** **recap**, **the problems are grazing areas first, cattle feed secondly, diseases thirdly, the** **quality of health care fourthly and the cost of care fifthly.**

**So, here are the difficulties and obstacles** **met** **in the production of milk.**

What can be done concerning the difficulties you have just mentioned?

For us, we need a good veterinary health officer and solve the food issue.

**To them,** **everything will be well if they have it all.**

For that, we will need people to help us by subsidizing our animal feed. For instance, a bag of “Ripasse” that costs 5,000 FCFA may finally costs us 2,500 FCFA.

**How do you plan to pay for the costs of care?**

We need all villagers to agree on one thing, that is choosing choose the people to help them so as to have our own partners who will assist us.

**You** **know that you** **said you** **would like to have a** **veterinarian and receive quality treatment**. **You would like to have goods** **drugs as well** **because** **you mostly buy from from** **street vendors.** **Concerning cattle feed, you also said that you want to have someone to subsidize it to you** **like** **so** **that** **it** **becomes affordable to you.**

**So we** **can say that we** **know** **how to solve the food issue.**

**You later said that care** **is** **expensive** **and not** **of good quality.** **I would like you to** **tell** **me** **what you think** **must be done to eradicate this.**

They said they not only want subsidies, but also quality treatment.

The reason is they buy some drugs in the neighbourhoods that are of no effect on animals when administered to them.

**Do you see it?**

Tablets or injections have no effect on animals.

It is therefore better for us to have someone who can bring us good drugs and treat our animals in case of diseases, like a veterinarian who will give us drugs knowingly.

**By the way, that is** **the problem; I have understood it.** **If one of your animals gets sick, you will buy drugs because they have told you that** **it can cure the animal.**

**You have not seen the veterinarian and that is why you do the work** **yourself.**

**So had the veterinarian** **been** **there,** **he** **would have prescribed drugs that could have cured** **the animal.**

He is the only one who can do it, but there is no pharmacy here. To have access to a pharmacy, we must go to NIAKHAR where they sell medications that are similar to those from street vendors. People selling real drugs are not there, it is clear.

The reason is I cannot see people like them. He can take a syringe and add little water to vaccinate the animal. Perhaps you; we would like to work with you.

**How to** **have a good veterinarian?**

It is necessary that people from the council help us by bringing a veterinarian who owns a pharmacy with all medications for whosoever wants to treat his animal.

**Who else apart from the council?**

I see no one else than the council.

**Of course,** **partnerships.**

Those that can help us are the rural council and a partner.

**We go to the next question**. **Have you** **mentioned** **that?**

**All right, the identified obstacles**.

**I** **have already said that; these are the solutions.** **What can cause these difficulties?**

So we must get a veterinarian, food and livestock care.

**What are the diseases that** **affect** **cows in the neighborhoods?**

Diseases that affect animals in the neighborhoods are: the lumpy skin disease which is manifested by pimples that appear on the skins of the cattle hides, wounds on the leg are bleeding, the three-day disease in the form of malaria, dermatophilosis which is also a skin disease and the swelling of the side of the animal.

**Which ones are transmissible to humans?**

The three-day fever is transmissible as well as the lumpy skin disease.

**How** **do they react** **when contracting an infectious disease?**

If contracting an infectious disease, we call for the veterinarian who heals our animals suffering from the three-day disease. Nevertheless, the same veterinarian cannot care for animals suffering from the lumpy skin disease. It is a very deadly disease that cannot be cured. It is very contagious and attacks very healthy cows.

We do nothing to protect us from these diseases. It is a very contagious disease that often contaminates herds even in the pasture or through water troughs.

**Now we are going to draw the table and classify the five diseases to assess the most dangerous among them. There are diseases which can be cured and others not.**

Those which kill animals the most are the lumpy skin disease and the three-day disease, followed by "lathio" or “dry skin”, then “léwrou” or a wound that animals keep on scratching all the time and which never get healed, and finally dermatophilosis.

**Among these five, which ones kill the most?**

The three-day disease, the lumpy skin disease, “lathio” or “dry skin”...

**It is a table.**

**They do not know a** **mode of transmission of an** **animal to** **man.**

**The severity of** **the disease** **is not only** **due to its common transmission** **from one animal to another, but also to its** **mortality rate.**

**How** **do you handle** **the health of your** **animals?**

They practise self-medication. When they identify a disease, they go in search of drugs to cure the animal by themselves. The reason is that if they call the veterinarian, he will not come or do what you expect of him. When we need him, he looks for alternative ways not to attend to the needs of the population.

**Does he refuse to come or what?**

I personally called him once.  He came and recommended me to tether the ox whereas it is an ox which cannot be tethered. Finally, we everything was done to enter the animal into the park so that it might be injected.

To move the animal, he fixes prices we cannot afford.

**Concerning vaccinations and others, how** **do you do?**

Some do it every 3 months, every 6 months or even every year.

He comes here only once a year, unless you have a sick cow to hope he comes.

**Do you have any** **methods** **of** **prevention for your animals?**

Yes, there are vaccines that are administered to our animals such that “ogometre”, “terra machine” and so on.

There are “lathio” or “dry skin”, the three-day disease, the lumpy skin disease and dermatophilosis.

These are the diseases we can prevent.

**Do these methods save** **the cattle?**

Yes, drugs are often effective.

**Who** **do you think must take care of the health of your** **animals?** **Is it you or other people?**

Yes, we sometimes take care of it or we leave it to the youngest.

Furthermore, they are often the ones to inform us of everything that happens in the herd. If an animal is sick, they tell us so that we can give instructions to follow.

**Do customers** **always** **buy**   **and come here again for milk**?

There are Fulani who come every morning to buy milk and even customers always draw closer to purchase milk.

**Do some consumers** **have specific requirements** **in relation** **to milk?**

Yes, they do because milk is often not good and only the woman processing it into curdled milk can inform us.

Once she pours milk into the buckets or even butter jar as she uses to do, she notices that there is much water at the bottom and some curdled milk on top. However, even cows developing disease symptoms produce such type of milk.

As he rightly pointed out, women often report this to us. Women take this water off because you can contract malaria when you drink it. Curdled milk can make you sick or even worse, meat can kill you if you eat it at night before going to bed. However, this water can be used for women who are about to give birth so as to ease labour. There can be water in milk without it being removed as well.

The distance to reach sales points can be so far that going to sell milk there is not even worth the fare. In addition, there may be so much milk at these sales points during the rainy season that there will be no customer, even if you sell milk at 100 FCFA.

Not to mention the lack of transportation means to sell milk.

**Do you have any questions for us?**

If we can get people who can help us overcome all our problems mostly related to cattle breeding and milk, we will be delighted.

Why have you come to meet us?

**This is conducted within the framework of a** **PhD thesis research study** **carried out by these English students.** **Their lecturer** **can even make suggestions** **to** **the Ministry of Livestock** **for something to be done in this sector. Let us take the example of impregnated mosquito nets**: **it was a PhD** **thesis that enabled him to know** **that malaria is widespread in Senegal and to do something** **about it.**

**END OF TRANSCRIPTION**
