# Supplementary material for: “Everything in this world has been given to us from cows”, a qualitative study on farmers’ perceptions of keeping dairy cattle in Senegal and implications for disease control and healthcare delivery
Source: PLoS One. 2021 Feb 25;16(2):e0247644. doi: 10.1371/journal.pone.0247644 (PMC7906343; doi:10.1371/journal.pone.0247644)
Supplement: S1 Data — (ZIP) [file pone.0247644.s001.zip › Data/23503 SND1 English final.docx]

**23503 SND1 Men**

Introduction in English (00 sec - 02 sec)

**We are going to talk about** **issues related to cattle breeding today**. **Each of you has a specific level of understanding of cattle breeding** **practice.** **Concerning the samples that will be collected today**, **if a cow disease is identified, the programme will** **provide medical care for the sick** **animals over** **a period of** **12 months.** **We have taken your personal contacts.** **In case a disease is identified, we will call you for medical monitoring.** **Everything that will be said here** **will remain confidential and** **will never go outside the framework of the study.**

**What are the benefits of your cow fresh or curdled milk?**

We do not sell milk here.

We are actually peasants. Milk is a supplement in our diet, because we already have millet that we use to cook every meal. Fresh milk can accompany couscous, curdled milk and porridge. We do not sell milk. The primary source of income for our households is agriculture, then animal husbandry.

**Beyond** **agriculture and** **animal husbandry, do** **you** **have** **other** **sources** **of income?**

After agriculture and cattle breeding come millet trade, corn, peanuts, sorrel...

**Do you have other** **sources of** **income coming from a close relative?**

We receive sources of income from the State. We will come back to it.

**Here, we would like to know if you receive financial assistance from relatives.**

No, we do not receive such support. Our children are still studying. They do not yet have paid jobs.

**Do you not have girls working as home servants in Dakar?**

No!

**Among you**, **are there** **others who receive family** **allowances?**

Yes, I do receive family allowances.

**At what level are you presently on this** **scale** **with regard to the relation between** **your** **household income** **ratio** **and** **these mentioned activities?**

**You will take the** **pebbles**. **If** **it is agriculture that dominates, put it; the same if it is breeding** **and as such if it is trade.**

We all think that it is agriculture, followed by livestock breeding and trade.

**Do you think** **that livestock breeding will be more important than** **agriculture** **in the future in terms of** **income for your household?**

Yes, we believe that animal husbandry will dominate in the future because the sale of a cow can exceed what is earned in agriculture for a season. The sale of a cow can still ensure the household daily expenses for a year.

What is now difficult is to have a good breed because of the lack of cattle feed.

**Do you think** **that agriculture will decrease at the expense of livestock breeding?**

We sincerely believe it.

**How many heads does the largest flock in the locality count?** **Do** **not** **mention a** **name.**

The largest herd has about 100 heads of cattle.

**On this scale of 0 to 100, where are you?**

**You know** **there are 1 to 100 cows.** **Where do you stand? Is it 10, 20 or more?**

**Has the herd increased or decreased during these last five years?**

It has increased.

**If** **it** **has** **increased, draw a line at the top and trace it at the bottom if it has decreased.**

As for me, mine has decreased.

**Where** **do you** **think** **it** **is going to be if it** **increases the next 5 years?**

**Is it here?**

Yes.

**And what about** **you?**

It is here.

Yes...

**If you gather** **the herds that are here and those who are in transhumance and if there is cattle feed, grass ...** **How many litres of milk** **may** **you get in a day?**

20 to 30 litres a day, but not 40.

**During the rainy season, can** **the** **one with the largest number of cows have** **40** **litres?**

No, because the entire herd is not here. Others are elsewhere in transhumance. However, the herd that is here on site may produce 20 litres or even 30.

**He continues to** **indicate and write in the** **document or** **table.**

**What caused your milk to drop off?**

It is lack of grass because if it rains, you can stay up to a month without finding grass. This is the most challenging period to get cattle feed. Here in the locality, a cow cannot eat much grass during the rainy season. Moreover, there is a shortage of grazing lands because they are all cultivated. The suitable period for much grass to grow for the cattle in the locality is between November and December. Grass is completely exhausted after this period.

**What strategy** **do** **you** **have** **to prevent** **the**s**e** **constraints?**

The only strategy I see is to go looking for grass while feeding the cattle at home.

As for me, the strategy that I find is to reduce the use of arable land at the expense of pastures. This will also negatively impact us because the crops will decrease.

Diop, I agree with him because the suitable solution for all of us is to reduce arable land to the benefit of cattle breeding. Here is an example that I have seen in Toukar and Mboyenne in the region of FATICK. They leave more space to breeding than to agriculture. However, we have not yet practised this strategy here.

In addition, there is another phenomenon. Land is running short, in function of the population and housing. In the past, the head of a family would dedicate a large piece of land to animal husbandry only. Nevertheless, with the increase of the population and the urbanization of villages, this can no longer be possible nowadays. There are some who leave their field fallow to fertilize their soil and get some grass. Field owners also refuse in case someone else brings along his herd for grazing, whereas uncultivated lands could be used by everyone in the past. It also indicates that it will be difficult to produce the same amount of milk as we used to have in the past.

**What can** **you do to increase the** **amount of milk produced** **by your** **cows?**

If we can get some assistance to have enough animal feed that throughout the year, our milk production will increase. For example, our herds grow depending on their diet. A bull which has eaten till satisfaction can normally mate; so does a cow. Otherwise, they will note mate frequently and some cows may even abort.

You earlier mentioned someone who harvests many litres of milk per day. This happens because he owns crossbred cows due to the artificial insemination he has done. Therefore we will be able to produce much milk if we can get them too. We would really like to follow his example.

**Will you encourage** **your sons to practise agriculture and cattle breeding?**

Yes, of course, because these are the two activities that make it possible for us to live. If you have three sons for example, at least one of them will take over these activities.

We know that you have come for a purpose, but we would be very happy if you can help us with insemination. Insemination is presently very expensive and extremely important as people know that it is of great advantage. It was provided free in the past, but one has to pay for it nowadays. Benefitting from insemination requires much money. That is why people in cities and villages run after that. To inseminate a cow, you need a million and one hundred thousand CFA francs.

We have so much need of these cows, but we lack means.

**Yes, it is true it was free of charge because even I** **had it.**

**If I understand correctly, you mentioned the lack** **of money and the** **lack of space for breeding**.

**Why can cows not have much milk?**

The reason is the animal feed that promotes milk production is expensive.

**And what else?** **Why has the practice of breeding become** **difficult?**

Grass is lacking, because the tops of all houses were made of herbs during harvests in the past, but that is no longer the case nowadays. The drought of these last years prevented the development of animal husbandry. I did not live the other periods, but I have found these last years that lack of grass is an obstacle to the good practice of breeding. There is no grass in the dry season too. Cows are forcefully given millet stalks and subsequently, you know that a cow which is hungry throughout the year round may not produce much milk.

**What are the** **biggest** **obstacles to good breeding practice?**

Shortage of grazing land, expensive livestock feed, animal diseases, lack of rain and lack of grass.

**What are** **the solutions?**

We count on your support to find strategies to properly feed the animals so that we can have milk.

To me, the best solution is to change the current breeding practice, that is, to start practise artificial insemination. People should continue to be educated because if the cow does not have a space to feed, it will not be able to be productive. The State or the government is often blamed meanwhile that should not be the case. One day, we met the Water and Forests Officer for permission to cut down trees to feed the herd, but he clearly told us that this was impossible. Animal husbandry should be practised differently because it is a heavy responsibility to feed a large herd of many cattle. Trees should attract the rain. A veterinarian once said to me   this: “What you Serer call breeding is not cattle breeding.  You accumulate a lot of oxen and you are unable to feed them. It is better to have a few and be able to feed them. Farming the same field for three successive seasons does not prop up the existence of grass on this area.

**Now let us** **talk about the diseases that** **affect** **herds.**

**What diseases can be observed in cattle breeding?**

Here, animals do not really contract spots. I have found that the disease that is mostly present here is trypanosomiasis. I also have noticed it. The lumps killed two cows of mine. It is manifested by the inability for the animal to walk.

I noticed in the area a disease called “sapha” which is a disease that also affects the legs of the animal. Last year, there was a disease that caused me to lose a cow. It is called   “ngaly”, which is a contagious disease.

**Does this disease still exist?**

Yes, I think so. The reason is because even while going to the veterinarian, he had administered a vaccine against this disease. Even so, the disease can attack the animal as long as he is alive. Maybe the vaccine is not working for cattle. Even concerning the spots, the veterinarian explains that it is the same; an animal can still contract the disease after being vaccinated.

There is also the three-day disease that attack animals and which is manifested as cramps. It also prevents the animal from eating and drinking. It remains motionless for 5 days before dying. This disease most often occurs during the dry season.

**What is this disease called?**

I do not know the name of the disease.

**Among the mentioned diseases, what are the most common ones?**

The lumpy skin disease, “sapha”, the three- day disease and "ngaly” and the contagious trypanosomiasis.

**What is the most serious between** **the lumpy skin disease and** **“sapha”**?

“Sapha” is more serious than the lumpy skin disease, because it is recurrent every year.

**What is the most serious between** **the lumpy skin** **disease and** **the three-day disease?**

The three-day disease is more serious.

**What is the most serious between the lumpy skin disease and “ngaly”?**

It is the lumpy skin disease because it is more common than “ngaly".

**What is the most serious between trypanosomiasis and the three-day disease?**

It is the three-day disease.

**What is the most serious between the lumpy skin disease and trypanosomiasis?**

The lumpy skin disease is the most serious.

**What is the most serious between trypanosomiasis and “ngaly”?**

Trypanosomiasis is the most serious.

**Do you** **think animals can transmit** **diseases to humans?**

In fact, my health knowledge is very limited in this area and only veterinarians will really be of great help to you.

I believe that the lumpy skin disease is transmissible to humans, because a cow isolated from the herd if it has contracted this disease.

**You earlier said that the most serious disease is “sapha”.**  **Why did you say that?**

This is because if the animal suffers from this disease, it will not live 24 hours.

It is serious because traditional healers were called to cure the disease in this past, but the animal would die before they arrived. The effectiveness or even the speed at which the sick animal dies makes us conclude that it is the most dangerous.

**How** **do you ensure** **your animals** **are** **healthy?**

In the dry season, we bring the animals to the veterinarian for vaccination or we do prevention when a serious disease is announced or heard.

**What is the frequency of these** **vaccinations?**

Animals are often vaccinated in the dry season, from January to June

Personally, my cows are vaccinated twice a year, except in case of unexpected illness only.

**When practising self-medication, where do your drugs come from?**

I do not carry out self-medication because I do not even know how to do it.

I do it, but all the drugs I use come from the pharmacy, and not from the black market. For these people do not have good drugs and may even sell you poor products.

**If you** **need advice or knowledge** **in animal health, where do you go to?**

Veterinarians do not advise us on animal health. What we do when an animal is sick is to directly bring the animal there for veterinary check-up. To sometimes prevent a disease, the veterinarian comes and goes round each owner’s pen to vaccinate the herds.

On the activity of the veterinarians, some belong to the private sector and others to the public sector. When you bring a sick animal to private structures, they will screen the animal and prescribe drugs. The animal might live or die. However the veterinarian from the public sector comes to meet us at the chief of the village with drugs that even private structures do not have. My horse got sick one day and I saw a private veterinarian; he told me that he cannot cure the animal and that I had to meet another veterinarian.

**Do you believe that veterinary involvement is useful to your herds?**

Yes, their intervention is useful because it happens that they heal our cows.

**If the animal** **is** **seriously ill,** **do you treat it yourself or do you bring it to the veterinarian?**

If you cannot go to his house, you pay for the transportation and the drug so that he may come and treat the animal on the spot.

**What disease** **can motivate you to** **vaccinate** **the entire herd?**

Diseases like “sapha” make us vaccinate the entire flock, because every minute spent can make you lose a cow.

**Who takes the responsibility to bring the cow to the veterinarian, the owner or the shepherd?**

We discuss this. The person who has more time can bring it to the veterinarian.

**Can the shepherd bring it without the** **owner's** **consent?**

No, because the owner's responsibility dominates.

**Does the consumer have some requirements concerning the milk quality?**

Yes, because the milk of a cow with mastitis is not consumable. If you milk this cow, its milk is a little reddish and inedible.

**Who handles milk at home?**

There are some who give it to their women, whereas others keep it to better manage themselves. Some offer it to their mothers if they are alive. Otherwise, they give it to the first wife.

**Can you** **recognize the milk that** **is dangerous for consumption?**

Yes, if it comes from a cow that has mastitis, then it is not good for consumption. We know nothing else apart from that.

**Can curdled milk from an animal with mastitis** **change** **color?**

It is reddish.

**How** **can you** **recognize** **consumable** **milk?**

If its colour is white.

**Con you contract a disease by consuming fresh milk or curdled milk?**

Yes, if the person is allergic to milk.

I have had an experience on that. In the rainy season, if I consume milk from a cow that has eaten fresh green grass, it can make me dizzy.

**Can we** **be** **contaminated** **if we eat the meat of a sick animal?**

I do not think it exists, because I have never seen it.

I have seen people who suffered from stomach ache and diarrhea because they ate the meat of a sick cow.

**What obstacles do you face in selling milk?**

No, we do not sell milk. All we have is intended to consumption.

To me, it has been for long that I have not seen milk. Shortage of healthy cattle feed causes our cows not to almost have milk.

**END OF TRANSCRIPTION**
