# Supplementary material for: “Everything in this world has been given to us from cows”, a qualitative study on farmers’ perceptions of keeping dairy cattle in Senegal and implications for disease control and healthcare delivery
Source: PLoS One. 2021 Feb 25;16(2):e0247644. doi: 10.1371/journal.pone.0247644 (PMC7906343; doi:10.1371/journal.pone.0247644)
Supplement: S1 Data — (ZIP) [file pone.0247644.s001.zip › Data/23504 K1 English final.docx]

**23504 K1 MEN**

Introduction in English (00 sec - 03 sec)

**What** **benefits** **has** **the milk that comes from** **your cow?**

Milk is of great advantage because if it happens there is no rice, milk can be exchanged with rice for household consumption. You can also offer milk as a gift to a loved one.

**Is this** **milk** **production** **the main source of income or are** **there** **other sources of income?**

Here, millet and groundnuts farming are our main source of income. Thereafter comes the cattle breeding activity.

**Apart from agriculture and cattle breeding, do you have another** **source of income?**

In addition to these two activities, we also practice small trade. On the weekly market day, we buy sheep, goat or cows that we can resell on the spot or wait until the next market day to resell and so on.

**Do you continuously practice these** **activities for 12 months?**

Yes, we practice it throughout the year.

**We will ask each of you to tell us his main activity** **in terms of household income.** **For these** **three aforementioned activities, we will use pebbles and put three pots.** **For example, if you find that cattle breeding provides the most** **in terms of** **income, then you place more pebbles and so on.** **And we will use** **this procedure for each of you.**

**Ibou, come and place yours.**

Ibou: Here, there is more to put. That is what exists here. Agriculture and cattle breeding dominate here.

**Wait**, **I take a picture.**

Normally, we were to start there before going to Dibocor.

**Are there no women here?** **We need to talk with them about** **milk.** **Even if there are two** **or** **three women, it is fine.**

**There are people who have** **come to** **talk to them**. **They** **are on the way.**

**You, come**. **Would you like to add pebbles on livestock breeding or on agriculture or would you like to leave it** **as such?**

Yes, I leave it as such.

**Does it happen the same in the rainy season as in the dry season?**

Yes, it happens as such.

**Can the fact that agriculture** **dominates livestock breeding change according to seasons?**

No, everything remains the same.

**Do you think that these** **activities will change in** **the future?**

No, we think that agriculture will always remain supreme, because there are a lot of difficulties in cattle breeding.

**How many cattle does the largest herd in your area have?** **The herds that you have here and** **elsewhere.**

The largest herd in the community has 80 oxen.

**Can I** **put 100 oxen?**

Yes, you can put 100 oxen.

**Let us now consider a scale**. **Where are you on a scale** **of 1** **to** **100?**

**There are people I have left at Ngyokhem**. **It is after them that she is asking.** **We** **went to Dibacord,** **the breeders' representative, but we normally had to come here first. The lady told us that as he is the representative, we had to contact him first before coming.** **She asks** **whether there are women involved in milk processing.** **If** **there are any,** **they should come.** **Even** **three are enough.** **There is someone who will come and speak with them.**

**What is the most important between** **agriculture,** **cattle breeding** **and** **trade?**

Cattle breeding is more important.

**Will this order** **be different in the dry season?**

No.

**Do you think that there will be a change in the years to come?** **Will you abandon agriculture at the expense of livestock breeding or cattle breeding at the expense of agriculture?**

We cannot do without agriculture. Trade often works and sometimes not.

**So there is no change**. **It will remain as it is.**

**Yes.** **Can you** **tell me if the largest herd reaches 100 or 200 cattle?**

**Do not say no; simply answer.**

The smallest has 80. Let me do it, 100 to round up.

**You, show me where you personally stand from 1 to 100. Are you closer to 1 or 100?**

**Draw it here.** **Yes.**

**Compared** **to these** **last** **five** **years, have you increased or decreased?** **If there is an increase,** **come below and come up if it has reduced.**

**Now, where do you think your herd will increase to in** **the** **next five years?**

**We will apply the same procedure for milk.**

**Concerning milk,** **where will you be on the scale over the upcoming next five years?**

**How many litres** **of milk does** **the largest milk producer** **in the locality have a day?**

The largest milk producer can reach the bar of 40 litres, two 20-litre cans, one in the morning and one in the evening.

And the smallest production has at least 1 litre a day.

**Now, where are you positioned in a scale of 1 to 40?** **Can you tell me where** **you stand?** **From morning to evening, you can** **get** **40 litres, 20 in the morning and 20 in the evening?** **Where do you stand on the scale** **nowadays?**

I am at 1 litre.

No, 3 litres.

**Was what you used to produce five years ago greater or lesser than 1 litre?**

**If what you have today is more, draw a line up;** **if what you have today is lesser, draw a line down.**

**In the coming years,** **if** **there is much grass and food,** **your milk production** **will increase.** **If** **you agree, draw a line** **up.** **If** **you are not, draw a line** **down.**

Yes, my production during the last five years was superior to 1 litre.

**And** **you,** **Diouf,** **how was your milk production during the last five years?**

DIOUF: My production during the last five years was superior to what I currently have.

**So for all of you,** **the amount of milk you used to produce during the last five years** **was superior to what** **you actually produce now.**

**Now, for each of you, can you show me** **on this scale your level of cow milk production of your cows in the coming years?**

**If you think** **there** **will be an** **increase,** **put it up** **for the next five years.** **If you also think** **it will drop off, position it below** **for the next five years.**

Yes, if I continue to invest in cattle breeding, the quantity will increase. However, if I invest elsewhere as in construction, the quantity of milk will decrease.

**What** **will** **cause milk to increase in the next five years?**

With the multiplication of cows, one can expect an increase in milk production in the future.

If you have two or three cows today, you can get five by the next season thanks to reproduction.

**You said that you used to produce much milk during the last five years and it has decreased now.** **What caused this production to reduce?**

This is because there is a noticeable drop in cattle feed, which has resulted in a decrease in cow mating. What promotes births within the herd is mainly if the bull feeds on millet in large quantities.

**Do you think that there** **will** **be an increase in milk production in the next five years?**

It will depend on God, because that if we have a good rainy season, this will sufficiently increase cattle feed and milk.

**Would you encourage** **your children to practice agriculture and cattle breeding?**

Yes, because the children now in charge of that are different from those who go to school and that is their choice.

**What obstacles do you** **face** **in the practice of cattle breeding and milk production?**

The difficulty we face in cattle breeding is grass shortage and the inaccessibility of cattle feed. This causes the animal to be hungry.

Animal disease is also an obstacle to milk production.

Shortage of grass in grazing lands considerably weakens animals as far as reproduction and milk production are concerned.

Cattle feed is extremely expensive.

And presently, we no longer have lands intended to cattle breeding due to lack of space; this phenomenon constitutes the major handicap to cattle breeding in the area.

**Among the** **mentioned obstacles,** **which one gets you tired the most?**

The first factor is the lack of space intended to cattle breeding.

Then come diseases and the cost of cattle feed.

**What solutions** **would you like to implement to solve these problems?**

First, we need assistance from the government and you.

**Now we are going to talk about diseases that can contaminate** **animals.**

**What are the diseases that can infect animals in** **your area?** **If you know** **the names** **of the diseases, tell me.**

There is a disease called “sapha”. It spreads during the dry season and it is manifested by the inflammation of the mouth of the animal, nasal flow and spots on the face of the animal. There is the three-day sickness that also spreads during the rainy season, which is manifested by the inability of the animal to eat or drink. There is the lumpy skin disease which shows spots all over the body of the animal.

There is another which is manifested by bloating and which kills the animal very fast.

**What is the disease that gets you tired the most**   **?**

It is the three-day sickness first because if the animal contracts the disease during the rainy season and stay in the rain, it can easily die. Then comes “sapha” which makes us tired, and lastly, bloating.

**Between** **the three-day sickness** **and “sapha”,** **what** **is the worst?**

The three-day sickness.

**Between** **the three-day sickness and** **the lumpy skin disease, what is** **the most serious?**

The three-day sickness.

**Between** **the three-day sickness** **and bloating, what is the most serious?**

The three-day sickness.

**Between** **the lumpy skin disease and bloating, what is the most** **serious?**

It is bloating because the lumpy skin disease is not very common in this area.

**In a nutshell, you have said that the three-day disease and** **“sapha” are the most** **dangerous.**

**Do you think that these** **mentioned animal diseases** **can be transmissible to humans?**

I know that “sapha” can be transmitted from one animal to another when they drink from the same container.

**No, I** **mean** **a disease that is transmitted from animals to humans**.

Actually, we just have never seen it, for even if you consume the meat of a sick animal, you will not get sick.

We no longer milk sick animals; their milk is left to calves.

**What do you refer to before saying that such disease is more serious** **than** **another?**

It is by considering the speed at which the animal dies after contracting such or another disease that we use to know the severity, as it is the example of the three-day sickness that kills animals quickly.

While, for example, an animal suffering from “sapha” can live for a week before being killed by the disease.

**Now we are going to talk about** **animal health care.**

**What do you do if an animal gets sick?**

If we find that an animal is sick, we call the veterinarian and we pay him for to check it up.

**Do you practise self-medication?**

If the disease is related to “sapha”, we use traditional medicine to cure them, using local tree leaves called    “sebe”. However, if it is the three-day sickness, we immediately call the veterinarian.

Also concerning the three-day sickness, there is a preventive vaccine that costs 100 FCFA because it is a vaccine subsidized by the State. Nevertheless, if you do not vaccine the animal until it falls sick, then you pay 1000 FCFA.

**How often are your animals treated?**

Vaccines are made annually, with the onset of the rainy season. During this period, the cows do not have enough strength because of food depletion.

**Where do the medicines you use to** **cure** **animals** **come from?**

We buy these medicines at pharmacies, but also at the market.

**If you notice an epidemic has broken out in your** **herd, where do** **you go** **to report?**

We directly go to the veterinarian.

**If you realize that a serious disease has contaminated your cow, what do you do?**

We call the veterinarian to cure the animal and we pay him.

**Do** **you** **think** **that vaccination is beneficial to animals?**

Yes, it is a very good thing because the disease we were talking about has just disappeared because of vaccination.

**What motivates you to go to the** **veterinarian?**

If our animal gets sick, we take it to the veterinarian.

**Who** **is responsible for the preservation of the herd? Is it the owner or the shepherd?**

The owner of the herd assists, even if the animals must migrate. He makes decisions.

**Do milk consumers** **have requirements concerning milk quality?**

Of course, because even me, I do everything to satisfy consumers with good milk.

**Do you have loyal customers who** **do** **not** **buy** **milk elsewhere?**

Yes, it happens. We have loyal customers who come to buy our milk with confidence.

**Can milk quality cause** **its non-consumption?**

On one condition, if the cow has mastitis. In addition, this milk is even yellowish instead of white.

**After milking the cow, do** **you sell the milk or do you give it to your wife**   **?**

No, we do not sell it. It is given to women. Nevertheless, it may also happen that a loved one is in needs it; it is then given to him.

**Do you have measuring instruments to know your cow milk quality?**

No, we do not have instruments to check the quality.

**What can make milk inedible?**

Except flies that have fallen into the milk, there is nothing else.

**You earlier said that a cow with mastitis does not have good milk.** **How** **can someone** **recognize milk of good quality?**

It can be recognized from the white color of the milk. A cow without mastitis has milk of quality.

It is also recognized when milk is covered with foam during milking.

**Can milk consumption make someone sick?**

Excessive consumption of milk can cause a disease, which is malaria.

**Can someone contract** **a disease** **by consuming** **meat from a diseased animal?** **Or can someone be contaminated by being in permanent contact with a sick animal?**

If you consume an animal, it can transmit you its disease.

**Have you ever seen that?**

Yes, we witnessed that, because after consuming this meat, they vomited and they had diarrhea.

**Is there a disease that** **only infects breeders?**

No, it does not exist among breeders. A person can get sick, but it is not because of animals. If someone is ill, his disease can be found in someone who does not live with cattle.

**What obstacles do you face in selling milk?**

No, we do not sell milk.

**We thank you for your** **trust and your presence.**
