# Supplementary material for: “Everything in this world has been given to us from cows”, a qualitative study on farmers’ perceptions of keeping dairy cattle in Senegal and implications for disease control and healthcare delivery
Source: PLoS One. 2021 Feb 25;16(2):e0247644. doi: 10.1371/journal.pone.0247644 (PMC7906343; doi:10.1371/journal.pone.0247644)
Supplement: S1 Data — (ZIP) [file pone.0247644.s001.zip › Data/23505 FK3 English final.docx]

**23505** **FK3** **MAN**

**We are in the village of** **Calome** **FK3.**

**As was done last time, we visited some houses.** **Today,** **we are going to discuss about breeding in relation to cows, milk or diseases.**  **We will now ask you questions and everyone’s contribution is greatly desired.** **Every idea is of interest to us.**

**Question 1: What is the use of a cow in your family?**

When you get sick, you can sell the cow and heal yourself.

The utility of the cow is vast, because their waste is good for fields. Milk is also good for children and the elderly. Even if the cow is dead, we eat the meat.

A cow is very important because when you are hungry, you can sell it and meet the need. You can sell it and marry a woman. Your house can also be demolished and you sell a cow to repair it. A cow is slaughtered for funerals and weddings too.

**Apart from cows, what else can help your families?**

Agriculture, however if you have a son in Dakar, he can send you something at the end of each month. If you have a problem, he assists you by sending money.

**And what else if you have someone who has a job elsewhere?**

At the end of each month, he can send us something.

There are also people who do business to help the family because if you neither cultivate, nor breed, then you will go to school and you can only do business. There is also trade.

**Apart** **from agriculture, livestock breeding and trade, is there anything else that can** **be a source of income?**

To me, that is just what I know.

**What can make the farmer progress?**

Apart from agriculture, it is livestock breeding or trade that makes the farmer progress. It's the three that I see.

**Are there masons** **among you?**

Yes, there are.

**Among these four activities that you have** **mentioned, what** **is** **the** **most important?**

It is agriculture, then livestock, trade and masonry.

**Do you think that the activities you have just mentioned will improve in** **the upcoming years, especially cows?**

We want what increases us but not what diminishes us.

**Among these aforementioned activities, what can cause the** **herd to decrease or increase?**

Agriculture can increase the herd, because when you grow plants, cows have food which allows them to multiply.

**In this village, how many cows does the smallest herd have? How many cows does the largest herd have too?**

The smallest herd can start with two cows and the larger flock can begin with at least 100 cows.

**During the past five years, how many cows did herds begin with to a** **minimum**?

The smallest herd started with five cows and the largest flock had 150 cows.

**In the next five years,** **how many cows** **do you think** **the smallest herd will begin with?**

10 cows. If you start with 10 cows and you have a purebred cow in the herd, this number can increase from 5 to 6 cows over the next five years. If it is a large herd with 100 cows, it can increase up to 150 cows over the next five years.

**In this dry season, how many litres does a cow producing less milk produce?**

It produces half a litre in the morning and half a litre in the evening.

If there is food, the cow can produce 1 litre in the morning and 1 litre in the evening.

**And if it is during the rainy season and** **there is** **food, how many litres of milk does the least productive cow produce in this case?**

In this case, the least productive cow may produce 2 litres in the morning and 2 litres in the evening. For a cow that produces much milk, the double can be obtained.

**In the dry season, if what you produce the most is 10, and what you produce the least is 2, so if in the rainy season, we will have the double** **or** **triple where we usually had 10.**

If in rainy season, where you normally had 10, you must be 20; where you had 2, then you must have 4. It makes more sense because you can only produce less when there is no food.

**They said that in the rainy season, you can get 4 litres** **in** **minimum** **quantity** **and you can have 20 litres per day at most.**

**Yes, that is it; it is like that.**

**Apart from** **cattle breeding, is there another activity you practice which can earn you an income in return?** **It may be agriculture or trade, or whatever you can and an activity that you do at the same time with cattle breeding.**

We have said it. It is agriculture, trade, masonry and breeding.

It is agriculture 1, breeding 1, trade 3 and masonry 4.

In the dry season, there is no agriculture.

**Is it** **trade that comes first?**

Yes.

**What is the following?**

It is trade that comes first during the dry season. Do you hear?

**Yes.**

Thereafter, it is breeding that follows, then masonry and lastly agriculture because it is practised in the dry season.

**So if it is in the dry season, the first activity is trade, followed by breeding, masonry and agriculture.**

**Now in the rainy season,** **agriculture comes first**, **livestock breeding in second, trade in third and masonry in fourth position.** **Okay.**

**What has caused these changes?** **The reason is if you look at the present situation** **in and the situation five years ago** **as well as your perspectives you make for the future, there** **have been changes.** **Then, what** **has** **caused** **these changes**   **in relation to** **milk** **production**?

**You started with 2 litres a day and have increased to 4 litres. You also** **said** **that you had 10** **to** **20** **litres five years ago.**

**Concerning breeding, you said from 5 to 150.**

**Now, what has caused these changes?**

It is the food that promoted these changes.

**And what else apart from food?**

There is no grazing area.

**And what other problem?** **You have** **talked** **about food. You have talked about** **grazing areas. What other problem does exist too?**

**He is talking about a storage place for millet stalks after harvest.**

**What? Is it an attic?**

**No,** **it is** **not a matter of attic,** **but of a place of storage.** **If someone** **could** **help them get it so that they could store cattle feed, it would be convenient to them.**

Three problems can be distinguished: feeding, cost and grazing area. These are the three.

**Now among these three that you have mentioned,** **which one comes first?** **Is it the cost of cattle feed, or cattle feed itself, or is it the grazing area?**

It is the cost that comes first.

**What after?**

After that come the grazing area and then, food.

But chronologically, the first issue is cattle food.

**Why the problem of food?**

The reason is they do not have where to eat. Now, even if there is to eat, the cost is challenging. That is why it must be classified as such.

**Therefore, there will be food first, the grazing area in second and the cost in third position.** **These are the changes in the past.** **So they explained the reasons too.**

**These are the** **changes in the past. Now, what are the current changes that** **have helped you to survive nowadays?** **Do you understand?** **That is, what changes have you set up and why?**

To me, if you have no solution and if you have three cows, then you sell one to feed the others.

**That is it; so it is trade.** **It is trade that we must put.**

It is from the trade that you buy food.

It is the trade which is at the top; it is from the trade that one buys food, but also drugs to cure cows.

**Consequently here, there may be a change.**

No, so far, that must be it.

**Yes, it must be that.**

**Therefore, diet and** **grazing area must be at the same level.**

However, Gomis, I think that over there, we have said that you sell to have food to feed them. Thus, trade must be in second position. Here too, when you even sell to buy food, the cost becomes very expensive.

**Yes, that is why there is trade; you sell to buy. However, the third point is the cost now.**

Yes, that is what I have told you. We say the same thing.

**Grazing areas can be put in the food.**

There is equality here.

**Anyway, these two things are related.**

**Do you think that what we milk will increase in the coming years?** **Do you think that milk production will be profitable in the future?**

Yes, of course, the production will increase.

**Do you plan getting your children involved in cattle breeding?** **Will** **you draw** **your children** **into livestock breeding?**

What?

**The question that comes before**…

Ha! Yes, yes, he thinks it is profitable in the future.

Ok, he says that the procedure requires that when you have two children, you send one to school and you let the other practise cattle breeding and agriculture.

**And if you have** **two children** **and both want to go to school what do you do?**

Consequently, you will no longer breed.

**It is cultural. Do not consider logic; it is their culture although it is not logical.**

**What are the obstacles and difficulties that you encounter in cattle breeding?**

**In livestock breeding and milk.**

**Yes,** **what are your difficulties** **there?**

We have already mentioned it here. It concerns the grazing area, because if you have a cow that eats enough food, milk production will increase.

**Now, this the question: do you face difficulties selling milk?**

We do not sell milk. We only offer it.

You come home to tell me “give me milk”; I give you and you go back. We do not sell it.

Offering milk is good.

**So you have a problem with grazing areas and food.**

Yes, that is it.

**Now as this is what you have identified as a problem,** **what** **is** **the** **solution to solve it?**

Reduce arable land that are used and to receive support for cattle feed.

**Who are the people who may initiate** **these changes and help milk production to improve?**

For that, it is up to the council to see how to subsidize it. It concerns the council and the government.

Fields and grazing areas should be delineated.

**The rural council is the only authority to do them.** **Only the rural council can help you with that.**

**Now, when it comes to diseases that affect your cattle,** **what** **diseases are most common** **are** **here?**

There are abdominal bloating, the foot-and-mouth disease, the lumpy skin disease and the three-day sickness.

**So, we have detected diseases affecting cattle productivity**. **Four diseases have been mentioned: the lumpy skin disease, the three-day sickness, the foot-and-mouth disease and abdominal bloating.** **These are the four diseases that have been mentioned by the population of** **Kalome** **3.**

**Now we will try to put them in order.**

**What** **is** **the** **most serious disease among** **these four?**

It is the three-day sickness. It is number 1.

**What comes next?**

It is the foot-and-mouth disease.

Hey, listen to me. Listen to me.

**Yes.**

The three-day sickness is more dangerous.

**Thus, it is the foot-and-mouth disease.**

The diseases that never fail are the foot-and-mouth disease and the three-day sickness. These last two rarely occur.

According to me, the three-day sickness is the first, followed by the foot-and-mouth disease and then the last two.

If you contract abdominal bloating, then you quickly die.

This is the one that is the most serious because when it affects an animal, it kills very fast.

That one, you can stay a year without seeing it.

You know, his cows do not move but our cows go till the Fouta. Consequently, if I tell you that the lumpy skin disease and bloating stomach are the most serious, then you should take it down. The three-day fever can kill, but not rapidly. However, these two kill swiftly.

**Do you** **know** **what** **we are going to** **do? We need to agree on both cases to move forward.**

So, we have to maintain both.

**Yes, we keep both.**

What is the name? “Amath”?

**No, it is “Ibou”.**

Ok, “Ibou”. Tell us.

You know, we are in the month of May. In the months of June and July, this one will come by force. This one occurs by force in January and February. When you enter the rainy season and when you finish it, these two over these appear. This is not fast but when it attacks the cow, it dies.

**But does that kill?**

Yes, the three-day sickness, it kills.

You know, the lumpy skin disease does not kill.

**Accordingly, we take** **both of them.** **There is no more comparison to make.**

**They agree on this principle, before we move on**. **They did not agree, but it took the intervention of the old man.**

**Do these diseases infect somebody?**

No, it does not contaminate.

**So the number six is ​​irrelevant.** **There is nothing.**

**Here, it is written, “If yes, explain”.**   **As it is “No”, there is** **no explanation.**

**What do you do to protect yourself or** **to cure these diseases?**

**Here too,** **there is nothing.**

**The table of comparison is here;** **we have already done it.** **The table is filled once in relation to the impact on cows and a second time in relation to the impact on humans.** **There** **is no impact on humans.**

**That is mortality because it kills.** **That is** **why you said that it is the most serious.**

**What are you doing to take care of your herd?**

They are vaccinated.

**Therefore, vaccination is carried out to ensure that the herd is healthy.  Vaccination is done.**

**When do you carry out vaccination?** **Is it once a year or every six months?**

Concerning the foot-and-mouth disease, vaccination is done in June.

**Thus, is it the only disease against which you vaccinate cows?**

Yes.

**Where** **do you vaccinate them?**

At the veterinarian.

**Where** **it is?**

In Niakhar.

**Do you ever buy tablets at the market?**

Yes. However, in case there is a disease, we call the veterinarian to come with drugs for him to vaccine the animal, because we have not been to school. You can go as such and say, “Sell me a fever vaccination” and they will sell you something else. If you need something, you have to see the veterinarian; it is safer.

**Okay. Therefore if you have an animal suffering from a serious disease,** **you will call the veterinarian.**

Yes. Yes, you take the animal to him.

**He had said that you do vaccination.** **It is not the same with the treatment; the treatment is when the cow is sick but vaccination is for prevention.**

The three-day sickness vaccine is given once a year.

**Is that vaccine good?**

Yes, it is good.

**Under which condition do you make decisions to go with the herd or not?**

We go there as part of prevention.

**Who makes decisions about the herd**, **the breeder or the shepherd?**   **Who is in charge in case of sickness?**

It is the cow breeder who is responsible for the cows.

**Do you have any customers** **to** **whom** **you** **sell** **milk?**

No, we do not sell milk. We just give to neighbours and consume it.

**Can milk look in such a way that you are unable to consume it if you take it home?**

If the cow has mastitis, milk can be milked and consumed.

**But do you sell it to someone?**

No, we do not sell it.

**Do you have ways to recognize good milk from bad milk?**

It is done after milking. If the cow has mastitis, then it is not good because there is water at the bottom.

**How can you** **recognize** **good milk?**

When it is milked, good milk has foam.

**Can someone get sick by** **consuming milk?**

No, you cannot get sick.

**Can someone get sick by consuming meat from a sick cow?**

Of course, you get sick.

**Have you ever** **seen it?**

No, never.

**Can someone caring for a sick cow get sick?**

No, we have never seen it.

**Can someone get sick by sleeping with cows?**

No.

**What difficulties do you face in selling milk?**

We do not sell it.

**Thank you for your participation.** **We will take milk** **for analysis.** **If we find a disease, we will come back.** **Thank you for your participation** **and your time**. **Your contribution has been very important to us and everyone will benefit from the results we will obtain.**  **Thank you very much.**

**END OF TRANSCRIPTION**
