# Supplementary material for: “Everything in this world has been given to us from cows”, a qualitative study on farmers’ perceptions of keeping dairy cattle in Senegal and implications for disease control and healthcare delivery
Source: PLoS One. 2021 Feb 25;16(2):e0247644. doi: 10.1371/journal.pone.0247644 (PMC7906343; doi:10.1371/journal.pone.0247644)
Supplement: S1 Data — (ZIP) [file pone.0247644.s001.zip › Data/24502 ND1 English final.docx]

**24502 ​​ND1 MEN**

**As I** **told** **you, we would like to talk about breeders' issues**.

**We have questions to ask you** **and you too** **will** **answer us on what you know.**

**They do** **not** **understand** **French too, but they will** **look** **for people who will translate it.** **He too has been** **sent** **to come and speak with you.**

**We** **do** **not** **take** **names**. **We** **only take signatures.**

**What** **are the** **benefits of fresh milk and** **curdled milk on household food?**

Milk is of vital importance in the household. It supplies food for dinner (couscous and fresh milk) and curdled milk is for meals.

Milk production has presently slightly decreased. Otherwise, milk revenue can support much household expenditure.

**What** **is** **the** **main source of income** **for your household?**

The main source of income for our household is agriculture.

**After** **agriculture, what comes next?**

It is animal husbandry that comes in second position.

**Do you have a commercial activity?**

Yes, we trade animals such as sheep, goats and others.

We also carry other activities because when I come back from transhumance, I go to Koumpentoum to trade agricultural products.

**Do you have other** **activities** **that are different from trade,** **agriculture and livestock?** **If we recap, we can say that agriculture is the first activity, livestock is the second and trade is the third.** **Beyond agriculture,** **livestock** **and trade,** **do you have other sources of income such as parents who financially assist you** **in your household?**

No, no one supports us in our households. We are the only contributor to our homes. We live out of the village for nearly 7 months, in search of other activities such as trade to secure enough to increase household resources. Sometimes, we go on transhumance for a slightly long period. It is at this moment that we sell some of our cattle to meet the daily expenses.

**For each of you, we will make a** **representation** **with stones for** **the activity** **he thinks** **is** **most** **important** **to him in** **terms** **of income.**

**For example, if you think** **it is** **agriculture** **that** **generates** **the most income in your** **household,** **you can place more stones on the** **illustration** **intended to** **agriculture.**

**If it is agriculture that is the most important, there will be more pebbles at this level.** **For example,** **you** **place** **10 pebbles** **for agriculture, 7 for livestock and 5 for trade.**

Here, it is agriculture that dominates. Even if we sell cows, those who grow plants will earn more than us. Just to tell you that you cannot do without agriculture. The reason is that it is agriculture that allows us to breed animals. Agriculture can make us traders because if you have a good yield, you can sell in large quantities. It is agriculture that has given birth to cattle breeding and livestock breeding to trade.

For me, the two are equal because livestock can be used as fertilizer for our fields, which will give us a good yield. Others will say that it is animal husbandry because they earn a lot of money. Yes, because even I would say it is breeding, for I no longer cultivate due to land shortage. Some of us practise agriculture, but I am for breeding. There is no rest in agriculture, it is routine. If it is at home, we will no longer practise agriculture thereafter. Here, it does not stop; there is market gardening at the end of the rainy season. Our soil is poor and there is no space for it.

**Is there anyone among you for whom animal husbandry is more important than agriculture?**

No, because the expenses agriculture presently meet, animal husbandry cannot do it, since if you favor livestock breeding and trade at the expense of agriculture, you may not cope as agriculture is vital.

Livestock and trade come from agriculture because it is thanks to agriculture that the two other activities are practised. However, animal husbandry also contributes to the development of agriculture through the fertilization of soils. They will complement each other.

**Do you take a break** **in agriculture?**

Of course, at the end of the year, we rest because the agricultural activities are over. This also continues till the month of June.

This break is taken because land shortage and the poor quality of the soil do not allow us to practise farming throughout the year. Concerning cattle breeding, if the herds in transhumance are back, they only have two weeks to graze all herbs in the locality.

**Presently, do you think that changes will be seen** **in the future?**

Only God knows.

**Give me your opinion.**

We believe that in the coming years, livestock breeding will dominate agriculture because the lands intended to agriculture are gradually becoming scarce.

The observation that is presently made is that cattle are growing while farmlands are reducing.

**Tell me: how many cows does** **the largest herd in the community count?**

Here in the locality, we have seen a herd that can count 150 oxen and the smallest herd can go up to a cow.

**Now,** **you have told me** **that** **the largest herd** **counts** **150** **heads.** **Each of you** **will point out to me** **the level** **at which he is.**

**From 1 to** **150, let each** **person tell me where the limit of his flock is. The person has to indicate where his limit is. In other words, you** **a draw line where you are closest. Indicate it if it is 10 and as such if it is 30. If what you possessed 5 years ago was more than what you have today, you draw a line down and** **if it was fewer than** **what you have today, you draw a line up.**

**If everything goes well in the next 5 years, where do you think** **your herd can increase to?**

I can have up to 150 oxen.

**If you have much grass** **and your herd grows to 100 cows, how many litres of milk can you have a day?**

I can have 35 litres.

**How many litres does the largest milk producer produce a day,** **including those which have migrated?**

The largest producer can reach 35 litres a day.

**Now, each of you will tell me your daily milk production status, be it presently, five** **years ago** **and in the next five years**.

No, because if they have no food, they cannot produce much milk.

**If what you have today** **is more, draw a line up** **and** **if** **this is not** **the case, you draw a line down.**

**If you consider five years ago, do you think that cattle breeding is the same today as before?**

We have noticed that there are changes in the production of cow milk.

**What** **has caused this change?**

The animals are sick and do not have enough to eat.

The main causes are: shortage of space intended to livestock and grass, animal disease, shortage of cattle feed, high price of cattle feed because a bag of “ripasse” costs 9,000 FCFA, poor and inadequate water quality, and the boreholes built in the locality have no water that is good enough for animal health care.

**Among** **the problems cited** **above, what are those that are most harmful to cow milk production?**

The first is the shortage of pasture, then water shortage and diseases.

**Categorize these** **barriers** **using these stones. Take the largest number of stones** **and** **place it on the corresponding obstacle.**

**Using these stones,** **you will classify them** **according to the severity of the obstacle.**

There is space first, then food, water and diseases.

**Who do you think can solve these obstacles?**

You, for example, through training, funding, assistance from the government, NGOs and partners.

**We will now discuss cow disease.**

**What** **are the diseases that** **seriously** **affect** **your cattle?**

Pasteurellosis, the foot-and-mouth disease (“safa”), the three-day disease, the lumpy skin disease and trypanosomiasis.

**Among these five, what is the most dangerous?**

The lumpy skin disease is the most dangerous, followed by the foot-and-mouth disease, "patrology" (pasteurellosis?) and finally the three-day sickness (bovine ephemeral fever).

**Now, are these diseases** **transmissible to man?**

We think that the lumpy skin disease can be transmitted to man, because it manifests exactly the same in humans.

However, we really do not have a profound knowledge to declare with certainty that a cow contaminates humans.

**Now** **among these** **diseases, which is the most recurrent in the area?**

**Which one occurs the most between the lumpy skin disease and the foot-and-mouth disease?**

The foot-and-mouth disease occurs the most.

**Between the lumpy skin disease and pasteurellosis?**

It is the lumpy skin disease.

**Which one occurs the most between the** **lumpy skin disease** **and the three-day sickness (BEF)?**

It is the three-day sickness.

**Which one occurs the most between the lumpy skin disease and trypanosomiasis?**

It is the lumpy skin disease.

**Which one occurs the most between the foot-and-mouth disease and the three-day sickness?**

It is the foot-and-mouth disease.

**Which one occurs the most between the foot-and-mouth disease and trypanosomiasis?**

The foot-and-mouth disease occurs the most.

**Which one occurs the most between the three-day sickness and** **pasteurellosis?**

The three-day sickness emerges the most.

**Which one occurs the most between the three-day sickness and trypanosomiasis?**

The three-day sickness occurs the most.

**Which one occurs the most between pasteurellosis and trypanosomiasis?**

Pasteurellosis occurs the most.

**Can these aforementioned diseases infect humans?**

We are not sure of that.

**You said that the** **lumpy skin disease** **is the most dangerous disease. Why do you say that?**

This is because this disease kills many cows and we believe that a killer disease is always dangerous.

**Now we will address the issue of animal health.**

**If you notice an illness on any cow of yours, what do you do?**

If our cow is sick, we automatically contact the veterinarian to explain the symptoms of the disease. If it is dangerous, we bring the cow to him for check-up and we pay him later.

**Where do the medicines that you use to care for your cows come from?**

The drugs we use, we buy them at pharmacies or markets.

**Do you think that these** **drugs are valuable to animal health?**

These drugs have advantages, as they protect cows against diseases.

Our main concern is that we do not have a park for vaccinating our cows.

We have been pleading with local authorities and they have made us a promise.

**Do you practise self-medication?**

Yes, we also do it because we cure pasteurellosis using a drug called “terra machine”. For this purpose, each breeder keeps a sample with him in case of needs.

**What do you do if you realize that a cow of yours** **is seriously ill?**

It often happens. We sell it at a low price. At times, we also let them die because there is no solution.

Furthermore, if the cow dies, a barrier is placed to avoid that it contaminates others. When it is dry, it is incinerated. There are some breeders who throw the corpses of their sick animals where other cows may be contaminated.

**Do you** **often do prevention?**

We do not know vaccines, but if a disease is announced, then we vaccinate all our cows. We vaccinate our cows against pasteurellosis, the lumpy skin disease, a disease called “nghali” in Serer and pestilence.

**Do you think that these vaccination campaigns are improving the lives of** **cows?**

Vaccines are important because we used to be afraid that our cows would migrate towards Djolof, but it is no longer the case nowadays. They are immunized.

**What can make you vaccinate your cows?**

If you observe spots on them, then they should be vaccinated.

**Who takes the responsibility of managing the herd? Is it** **the owner or the shepherd?**

It is the owner who principally makes decisions regarding cows.

**Do customers** **buying** **your milk have a preference concerning the quality?**

No, they do not have this problem. They come to buy and go back.

**Do you have loyal customers who only buy your cow milk?**

Yes, we have customers. They often call us on the phone to place orders.

**Can consumers distinguish milk in terms of quality?** **What can make milk bad?**

It is when a cow suffers from mastitis. When its milk changes color, nobody can consume it. Otherwise, it is given to dogs.

**After** **bringing milk, do** **you sell it or do you give it** **to** **your wife?**

There are people who do not trust their wives and keep it. Others rather give it to their wives.

**Do you have measurement instruments to know if the milk is good or bad?**

No, you cannot check it. Also, if you see a calf which grows up fast and which is healthy, we can recognize that this milk is of good quality.

The quality of the milk can be recognized by the foam.

**Do you think somebody can contract a disease by consuming milk?**

Malaria, laziness and drowsiness may be caused by milk consumption.

**Can somebody contract a disease by eating meat from a sick animal?**

Yes, it can cause stomach pain, vomiting or diarrhea.

**Have you seen a person who** **has contracted** **a disease by merely touching cows?**

There are cases of three-day sickness that attacks animals during the rainy season. These diseases can be transmittable.

**END OF TRANSCRIPTION**
