# Supplementary material for: “Everything in this world has been given to us from cows”, a qualitative study on farmers’ perceptions of keeping dairy cattle in Senegal and implications for disease control and healthcare delivery
Source: PLoS One. 2021 Feb 25;16(2):e0247644. doi: 10.1371/journal.pone.0247644 (PMC7906343; doi:10.1371/journal.pone.0247644)
Supplement: S1 Data — (ZIP) [file pone.0247644.s001.zip › Data/24503 ND2 English final.docx]

**24503 ND2** **MAN**

Introduction in English (00 sec - 05 sec)

**What we must discuss with you** **is everything that concerns cattle breeding, milk cow and also animal diseases.** **The people with me** **are veterinarians.** **This is** **their area of ​​expertise.** **My name is Idrissa Faye** **and** **I am here to help them translate from Serer to French.** **They,** **these are English, her name is... No name will be mentioned in the discussion.** **We need the participation of everyone because everyone’s idea is of interest to us.** **Now, we will start the discussion.** **We need the participation of everyone because everything we say here**, **they will group and analyze them to take that which is more important.** **So, everyone has to share his point of view.**

**What is the use of cow and milk in your family?**

If it is milk, the family consumes it and makes money.

**Is it milk that allows you to survive in your families?**

Agriculture is the most significant. Then come animals.

Yes, he is right. Agriculture is the most useful, and then come animals. You can sell animals and earn money.

**Apart from milk and animals,** **do you practise another activity which helps you with the expenses?**

Maybe having someone who can help or perhaps having other knowledge. You can also trade animals.

**Do you have a family member elsewhere who is helping you?**

No, not yet. Our business is to sell our animals and produce milk.

**You have said that agriculture is your main activity, followed by livestock and then trade.** **Do you just practise these** **three activities?**

Yes, these are the three activities we do.

**Out of these three activities,** what **is the** **most important?**

It is agriculture, because you cannot feed the animals when you do not grow plants. After comes animal husbandry and then trade.

**If, for example, agriculture represents 10 pebbles, how many pebbles will represent animal husbandry?**

Animal husbandry will only represent three pebbles ahead of agriculture.

If it was me, breeding would represent 6 pebbles.

**And how many does trade represent?**

Trade is 3 pebbles.

I will explain why agriculture comes first, because all we eat comes from agriculture, as well as cattle feed. That is why agriculture is a priority.

**Are you doing for the two other activities what you do for agriculture?**

No.

**Do you think that animal husbandry or trade may be ahead of agriculture** **in the upcoming years?**

Even if breeding and trade grow a lot, agriculture will always be ahead.

**What do you think you can do** **for milk production to increase** **in the years to come?**

To have that, you must have food in quantity and quality to be able to feed the livestock well.

**How many cows do you think the largest herd has?**

The largest herd here can go up to 140 cows and the smallest flock has a cow.

**Each person can come to draw a line in this table that indicates the number of cows** **he has, without saying the exact number.**

**In the next five years,** **do you think that your livestock** **will** **increase in case there is grass and much rain?**  **How far can the production increase if the cows lack nothing?**

**I am going to draw another table so that everyone can fill in.**

The herd could increase of 5 cows at most.

**If cows lack nothing, how many litres** **of milk do you think they will produce?**

If you have 150 well-fed cows, you may have two cans of 20 litres of milk per day, one in the morning and another one in the evening.

**Compared to the number of cows** **you have,** **each of you ticks** **in the table how many litres** **of milk you can have a day**.

**If what you used to produce as milk in the past is more** **important** **than what you produce today, draw a line below. If your present production exceeds your past production, draw the line up.**

**If everything you expect as cattle feed is available in quantity and quality, what will be the milk production?**

**What is causing milk production to decrease today compared to before?**

The reason is there is no food any longer. Moreover, there is no longer grazing area as before. In the past, farms were filled with grass in abundance and there was no need to fetch it home. That is what has brought difficulties.

**What can you do to make everything be as before?**

After the harvest, grass is brought home. If the animals eat all that is left in the bush, you begin to give them the stock that is at home.

We wish anyone can help us and leave a space in the forest for breeders.

**Will you encourage your child to become a breeder or farmer?**

Here, you must teach your son animal husbandry because it is our culture and that is what we know.

Our parents taught us how to cultivate and breed, but we would like our children to go to school. If you have three children, one may go to school, the other cultivates and the last one breeds.

**You have just given the reasons why cattle breeding no longer works.** **That is why you say that milk production has decreased.**

**Among the reasons why milk production has dropped off, which is the most common?**

First is the shortage of grazing lands, then high food prices and diseases because veterinarians do not come here to take care of animals.

**You said that milk production used to be** **more important than that of today. What do you think could solve this problem?**

The government and partners coming from abroad can help us.

**Do you think partners can assist all herd owners in Senegal?**

I think yes. Even if they cannot help everyone, they can at least help the maximum.

**So in summary, the government or** **partners can help you,** **but you** **cannot help yourself.**

**What** **are the diseases that are most common** **among your animals?**

Dermatitis is more common in cows. There is the “dasso” which causes the cow to lose weight, the foot-and-mouth disease that swells the cow feet, especially as it can last for two months on fat cows, and finally trypanosomiasis.

**What** **is** **the** **most serious disease** **in order?**

They are the foot-and-mouth disease and trypanosomiasis.

**Can these diseases infect** **a** **person?** **Or can one eat meat from a sick cow and get contaminated?**

No, I have never seen it. Nevertheless, cows can contaminate among each other, especially for dermatitis. I once saw a person having spots, but I do not know if cows had contaminated him. Concerning meat, I have never seen it too.

**Do you protect yourself against sick animals?**

After curing the animal, you buy kerosene to clean yourself, or you use soap or “Cresyl”.

**What disease is the most common?**

It is “sopha”, then the three-day disease and the lumpy skin disease.

**Between** “**tako”** **and** **dermatitis, which is the most common?**

It is dermatitis.

**Which is the most common between** “**niadio”** **and** “**sontos”?**

It is “niadio”.

**Why do you say that this disease is more serious than another** **disease?**

The foot-and-mouth disease is the most serious one because when it infects the herd, it kills several of them before being eradicated.

**What are you doing to take care of your herd?**

In the rainy season, they are vaccinated and given tablets.

**What do you do if** **you** **have** **animals suffering from a very serious illness?**

In this case, the veterinarian is called to come and check.

**Apart** **from tablets given to you by the veterinarian,** **what other tablets** **do you buy at the market?**

We buy “Nabote” and “Theramicin”. Then, we call the veterinarian to come and administer them.

**Who makes decisions about the herd?** **Is it the breeder or the cow owner?**

It is the cow owner who is responsible for everything that must be done on cows.

**Do you check first the milk you consume to see if there is a problem or not?**

Yes, we check before consuming.

**Do you have permanent customers** **to whom** **you sell?**

If the milk is clean, the customer will always come to you. However if it varies, the customer will change supplier. If the milk is clean, the customer trusts you. If he comes and you are not even there, he waits for you.

**Are customers are not demanding?**

Yes, there are some.

**Do those who buy from you to resell have some requirements?**

No, because they bring their bottle, you pour them milk inside and they leave it to ferment in the bottles before selling.

**Can milk have such an appearance that you do not consume it if you bring it home?**

Yes, because if the cow has mastitis, its milk is yellowish and thus cannot be eaten. Sometimes, milk has such an appearance that even the calf does not like it.

**After milking, do you** **give** **the milk** **to the woman for sale** **or do you sell it yourself?**

The woman will sell it.

**Do you have the means to distinguish good milk from bad milk?**

It is after milking the milk that you can know it.

**What can you do to make milk spoil**   **?**

Water. When you add water or salt, it does not ferment. If the container is not clean, it gets spoiled as well.

**How** **can you** **recognize good milk**   **?**

Good milk foams during milking.

**Cam someone get sick by** **consuming milk?**

Yes, of course. If you just milked milk and you immediately drink it, you will automatically have stomach ache. However I do not think you can get sick.

**Can someone get sick by eating the meat of a sick cow?**

Yes, it happens.

**Have you once seen it?**

Yes, it happened here. The cow was sick and was administered an injection. Then it was killed for consumption and those who ate it had stomach ache.

**Can someone who cares for a sick cow get sick?**

No, we have never seen it.

**Can you get sick by sleeping with cows?**

No, you might be tired.

**What difficulties do you face in selling milk?**

If you are far from the village, you cannot sell because milk will get spoiled. In addition, it is sold very cheaper if there is much.

**Thank you for your participation**. **Now we would like to take a milk sample for analysis.** **If we find a disease**, **we will come back here**. **Otherwise we will not.** **Thank you for your participation.**

**END OF TRANSCRIPTION**
