# Supplementary material for: “Everything in this world has been given to us from cows”, a qualitative study on farmers’ perceptions of keeping dairy cattle in Senegal and implications for disease control and healthcare delivery
Source: PLoS One. 2021 Feb 25;16(2):e0247644. doi: 10.1371/journal.pone.0247644 (PMC7906343; doi:10.1371/journal.pone.0247644)
Supplement: S1 File — (PDF) [file pone.0247644.s002.pdf]

Senegal Focus Groups-Male farmers

The group will include no more than 10 farmers. Those included will be farmers who keep one or more cows for milk production for either home consumption or selling. Individuals must be over 16 years of age.

Introduction and obtaining consent

Good Morning/Afternoon, I am _____________ and my assistant(s) is/are _________ (and __________). Today we are going to be discussing issues within dairy farming. We want to talk with you because we feel that that we can do a better job in promoting development of the sector if we talk to farmers like yourselves who are very knowledgeable and experienced in the area of dairy farming. Before we begin, we would like to tell you a little more about how the discussion will go and ask your permission to participate.

You are being asked to take part in a research study. It is entirely your choice and completely voluntary. The decision to join or not to talk with us today will not cause you to lose any benefits. Your participation is not paid, but we brought some refreshments.

If at any time you no longer want to participate in the discussion, you are free to leave.

Everything you say today will be kept confidential, we will summarise the things you tell us and combine it with other focus groups we are giving. We will report quotes of what is said but these will not be attributed to anyone by name. Everyone here must agree not to talk to other people about any specific person in the group or what they said during our discussion today. The information we talk about will be shared with the research team, but we will remove all names so they will not be able to tell who said what in the discussion. A study number rather than your name will be used on study records. Your name and other facts that might point to you will not appear when we tell other people what we talked about today. Government agencies and university employees overseeing proper study conduct may look at your study records. We will keep any research records we produce private to the extent we are required to do so by law and university regulations.

Our assistant, _____________, will be taking some notes, but he/she will not be able to write down every word that is said, so we would like to tape-record our conversation so we can listen to it later and make sure we understand everything you told us. The recording will be stored in a secure location and will not be accessible to anyone outside of the research team. We would like to take some photos

during the session. If you are happy to appear in photos please take and wear a sticker, if you are not wearing a sticker we will not include you in any of the pictures.

Is it alright with everyone if we use a tape-recorder and take pictures? [If yes – "Thank you for your willingness to be recorded and to allow us to take picture".] If not that individual may leave the session.

Do you have any questions about confidentiality, the study or what we are going to do?

We will pass a participation list now for you to sign, which also confirms that you agree to participate in this study. [For illiterate groups, consent will be obtained verbally.]

Focus group discussion

My job as moderator of the group is to ensure we cover all the topics that we have planned to discuss. There are no wrong answers and we would like to hear all your honest opinions and views so please feel free to speak openly during the discussion.

To start of lets introduce ourselves and say how many dairy cows you keep and how long you have been involved in dairy farming.

Importance of dairy to overall livelihood activities now and in the future

1. Firstly we will discuss how important dairy farming is to you and your family, so let's start by discussing how important dairy farming is for your household's income? What streams of income do you have in your household and which ones are the most important?

   (PROMPT: Do you rely on any money transfers from any family or friends outside of the household?)

ACTIVITY: After discussing income revenues, everyone is given 50 beans and asked to stack them on the relevant activities according to how much of their income comes from each. After this they are asked to stack the counters on the activities according to how much of their time they spend on each.

2. Do you expect this to change over the coming years, eg will you want to be doing more or less dairy farming in future or more or less of something else?

Barriers within the dairy industry

ACTIVITY: Ask participants what the largest herd size is in their area. A slopping line will be drawn to represent from 1 cow to this largest herd size. Each participant will be asked to mark where they were on this line 5 years ago, where they are now and where they want to be in 5 years time (they can go beyond the current maximum).

This will be repeated but thinking of amount of milk produced per week in peak season.

(PROMPTS: after activity, what caused the change in the past?

what changes did you make and why?

Do you see a lucrative future for dairy farming?

Would you encourage your children to undertake farming or other jobs?)

3. What are the main barriers or difficulties you face in dairy farming?

ACTIVITY: Once barriers have been identified, show pictures representing these barriers and ask people to place them in order from most important to least

(PROMPTS: what can be done about these barriers?

Who are the people who can make these changes or help the dairy industry to grow/improve?)

Views on productivity diseases

4. What diseases do cattle in this area suffer from?

5. Can any of these diseases pass to humans? If yes, which ones and how do they pass to humans?

6. What things do you or people in the area do to treat or prevent these diseases?

ACTIVITY: PAIR WISE RANKING MATRIX [facilitator lists the five most relevant diseases in a pair wise matrix and asks people to compare the top and side to say which is of most concern for each pair]

Data capture:

	Disease 1	Disease 2	Disease 3	…	…	Number of preferences
Disease 1						
Disease 2						
Disease 3						
…						
…						

The matrix is filled in once for the impact on cattle and once for the impact on humans.

(PROMPTS: why do the more important diseases concern you, what makes them worse than the other ones?)

Opinions of healthcare provisions and interventions

Lastly, we will discuss the healthcare options for dairy cattle,

7. Please describe how you manage the health of your cattle?

   (PROMPTS: what routine health activities do you carry out and when?

   Where do you go for dairy healthcare products and information?

   What do you do if you have very sick animals?

   Is this service adequate?)

8. Who uses vaccines and what are they for?

   (PROMPTS: How do they work and are they worth paying money for?

   What makes you decide to vaccinate for some things and not others?)

9. Whose responsibility is it to deal with cattle diseases?

10. Is there a demand for safe/reliable milk from consumers?

    (PROMPTS: do people buy from the same sellers once they establish trust?

    Do consumers ask for certain properties in milk?

    If for home consumption is there certain qualities to the milk which would make you not drink it?)
